# Aberrant medial entorhinal cortex dynamics link tau pathology to spatial memory impairment

**DOI:** 10.64898/2025.12.30.696887

**Published:** 2026-02-06

**Authors:** Taylor J. Malone, Kyle Cekada, Jean Tyan, Lujia Chen, Garret Wang, Yi Gu

**Affiliations:** 1 Spatial Navigation and Memory Unit, National Institute of Neurological Disorders and Stroke, National Institutes of Health, Bethesda, MD 20892, USA.; 2 Current address: Department of Psychological and Brain Sciences, University of California, Santa Barbara, Santa Barbara, CA, 93106, USA.; 3 Current address: Division of Clinical Geriatrics, Center for Alzheimer Research, Department of Neurobiology, CareSciences, and Society, Karolinska Institutet, 171 77 Stockholm, Sweden; 4 Current address: Medical Scientist Training Program, Medical College of Wisconsin, Milwaukee, WI 53226, USA.

**Keywords:** spatial learning, spatial memory, tauopathy, Alzheimer’s Disease, medial entorhinal cortex, cognitive map

## Abstract

Tau pathology in the entorhinal cortex (EC) is associated with spatial memory decline in aging and early-stage Alzheimer’s disease, but its impact on EC computations during learning is not well understood. We performed longitudinal two-photon calcium imaging of layer 2 excitatory neurons in the medial EC (MEC) of PS19 tauopathy mice over 10 days of an operant spatial learning task. Male PS19 mice showed marked learning impairments accompanied by dysregulated MEC activity and unstable spatial coding. Their activity also showed weakened representations in cue-poor relative to cue-rich regions, correlated with attenuated speed modulation. These changes suggest that impaired path integration destabilizes MEC spatial maps, leading to impaired spatial memory. In contrast, female PS19 mice exhibited only mild behavioral and neural deficits despite a comparable tau burden, suggesting sex-specific resilience. Among MEC cell types, pyramidal cells accumulated more phosphorylated tau than stellate cells and displayed the most severe functional disruption, linking cellular tau load to circuit dysfunction. Finally, general linear models of MEC activity reliably predicted learning performance, highlighted particularly strong contributions from non-grid and pyramidal cells, and accurately classified PS19 versus wild-type mice. These findings identify aberrant MEC dynamics as a key circuit mechanism underlying tau-related spatial memory deficits and point to early diagnostic and circuit-targeted therapeutic strategies.

## INTRODUCTION

Tau pathology, characterized by the aggregation of microtubule-associated protein tau (MAPT), is a defining feature of Alzheimer’s disease (AD) and other tauopathies^1,2^. In the earliest stages of AD and aging, tau pathology prominently involves medial temporal lobe structures, particularly the entorhinal cortex (EC), which is among the earliest cortical regions to show neurofibrillary tangles (NFTs) and hyperphosphorylated tau inclusions^3–5^. The EC is a central hub within the hippocampal-entorhinal circuit^6^ and is crucial for spatial representation and memory, as demonstrated by fMRI and lesion studies in humans and rodents^7–13^. Consistent with this role, early AD is characterized by deficits in encoding^14–18^, consolidating^19^, and retrieving^16–21^ spatial memories, as well as in allocentric^14,15,22–25^ and egocentric^14,22,24,25^ spatial representations that support cognitive map construction. In addition, NFT accumulation increases with age independently of AD status, becoming highly prevalent in older individuals, and correlates with spatial memory impairment during normal aging^26–28^. In contrast, amyloid-β, another hallmark of AD, shows less regional specificity for the EC in early AD and is less prevalent in the EC than tau pathology in normal aging, with many older individuals showing substantial entorhinal tau in the relative absence of amyloid-β^4,5,26,29,30^. Amyloid-β pathology is also less predictive of memory deficits^27,31^. Collectively, these findings implicate the EC as an early locus linking tau pathology to spatial memory decline in normal aging and AD.

Additional support for this hypothesis comes from studies on EC neural dynamics. In rodents, the medial entorhinal cortex (MEC) contains a diverse population of functional cell types that encode spatial and self-motion information^32–38^. Among these, grid cells are the most extensively studied and fire in a triangular lattice pattern in open arenas^32^. AD mouse models that recapitulate tau pathology, with or without amyloid-β, reveal widespread disruption of MEC activity^39–42^. Grid cells in these models exhibit hypoactivity, disrupted field periodicity, reduced spatial information, and impaired speed modulation^39,40^. The activity of non-grid cells and interneurons is also affected to various extents^39,40^. Additionally, *in vitro* electrophysiology studies report altered intrinsic firing properties of MEC cells^41,42^ with variability across age^42^, subregion^41^, and cell type^42^. Lastly, human fMRI studies reported reduced grid-cell-like activity in the EC of older adults and of young adults with genetic risk for AD^9,11^.

Despite these advances, it remains unclear how EC neural activity contributes to spatial memory deficits associated with tau pathology. Current studies suggest that spatial memory formation comprises a rapid initial encoding phase followed by several days of memory consolidation^43–46^, during which behavioral performance gradually improves^47–49^. To date, however, no studies have longitudinally tracked MEC activity across the full course of this process and related MEC activity to spatial learning performance in the context of tau pathology. Instead, most prior work has examined MEC activity at isolated navigational time points^39–42^. Furthermore, some studies have investigated MEC activity under conditions of high amyloid-β burden^50–54^ or severe neuronal loss^39^. The presence of amyloid-β can obscure tau-specific effects on neural activity, particularly in aging-related tau pathology. Severe neuronal loss is also less representative of aging and early AD, when overt atrophy is not yet present as in symptomatic AD and other tauopathies^55–57^, and may confound the specific effects of tau pathology on neuronal function at the cellular level through network-wide disruption. Together, these limitations hinder the identification of MEC neural activity features that contribute to deficits across the full trajectory of memory formation under tau pathology in aging and early AD.

To address this gap, we used two-photon imaging to longitudinally track calcium dynamics of layer 2 MEC neurons in the PS19 tauopathy mice^58^ during an operant spatial learning task across 10 days in virtual reality (VR) environments. PS19 mice express human tau bearing the P301S mutation that causes familial frontotemporal dementia in humans^1,59^ and are widely used to model tau pathology relevant to AD^60^, as they develop robust, progressive tau accumulation from ~5–6 months of age across multiple brain regions^58,61^, including the MEC^61^, followed by deficits in hippocampal- and entorhinal-dependent spatial memory at 6–8 months^60–65^. More pronounced hippocampal–entorhinal atrophy typically emerges later, around 9–12 months^58^, allowing us to examine how MEC neural dynamics contribute to spatial deficits before prominent neuronal loss, a disease stage that more closely resembles aging and early AD.

We found that compared to wild-type (WT) mice, male PS19 mice demonstrated severely impaired spatial learning. Their MEC activity showed reduced speed modulation, lower activity map stability within and between days, and failure to develop a spatial map shared by other groups. Their MEC activity was abnormally anchored to visual cues, suggesting disrupted path integration. In contrast, female PS19 mice showed limited spatial learning and MEC activity deficits. Activity deficits were strongest in pyramidal cells, which accumulated more phosphorylated tau than stellate cells, particularly in males. Finally, generalized linear modeling linked altered MEC activity—especially in non-grid and pyramidal cells—to spatial learning performance and reliably distinguished PS19 from WT males. Together, these findings highlight EC circuit dysfunction as a key contributor to tau-pathology-related spatial memory deficits in aging and early AD and identify the EC as a potential target for early diagnostic and therapeutic interventions.

## RESULTS

### Pyramidal cells in PS19 mice show increased tau pathology

**Figure 1:**
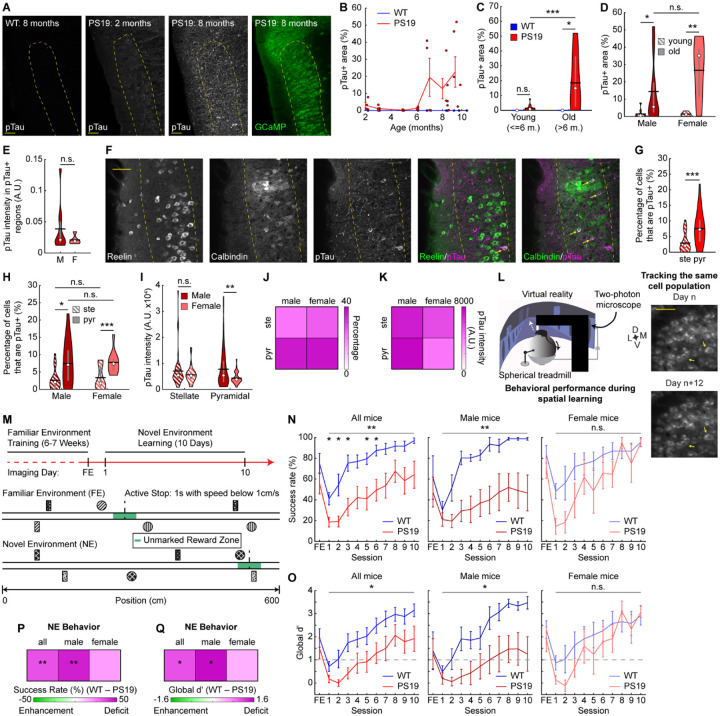
Male PS19 mice show behavioral deficits in an operant spatial learning task **A.** Left: Example images showing pTau accumulation in WT and PS19 mice of different ages. Right: GCaMP6f in an 8-month PS19 mouse. Dotted yellow line indicates MEC layer 2. Scale bar: 50μm **B.** Quantification of pTau accumulation in MEC layer 2 as a function of age. Dots represent the exact age of individual mice. Lines represent ages pooled into similarly aged clusters. **C.** pTau accumulation in MEC layer 2 in young (age<=6 months, m.) and old mice (age>6 months). Unpaired Student’s t-test. **D.** pTau accumulation in MEC layer 2 in young and old PS19 mice split by sex. Unpaired Student’s t-test. **E.** Average pTau intensity in pTau positive regions of MEC layer 2 in old male (M) and female (F) PS19 mice. Unpaired Student’s t-test. **F.** Example images showing the overlap of reelin (stellate cell marker) and calbindin (pyramidal cell marker) with pTau accumulation. Dotted yellow line indicates MEC layer 2 boundaries. Arrows indicate overlap with pTau. Scale bar: 50μm **G, H.** Percentage of stellate (ste) and pyramidal (pyr) cells overlapping with pTau+ cells in all PS19 mice (**G**) or in PS19 mice split by sex (**H**). Paired/Unpaired Student’s t-test. **I.** pTau intensity in stellate and pyramidal cells that overlap with pTau+ cells in PS19 mice split by sex. **J.** Heatmap summarizing stellate and pyramidal cells overlapping with pTau+ cells in PS19 mice by sex and cell type. Color represents mean value in **H**. **K.** Heatmap summarizing pTau intensity in stellate and pyramidal cells overlapping with pTau+ cells in PS19 mice. Color represents mean value in **I**. **L.** Experiment schematic. Left: virtual reality system, adapted from^68^. Right: Example field-of-view (FOV) of calcium imaging showing reliable cell tracking across days. Arrows point to examples of tracked cells. D: dorsal; V: ventral; M: Mediolateral; L: Lateral. Scale bar: 50μm. Full FOV is 750μm × 750μm. **M.** Task schematic. **N.** Percentage of laps that mice successfully stopped to receive water reward across learning in all mice (left), male mice (middle), and female mice (right). **O.** Global reward zone discrimination (global d’) across learning in all mice (left), male mice (middle), and female mice (right). Horizontal dotted line represents learning threshold. **N, O.** Horizontal gray bars indicate p values for the group difference (two-way repeated measures ANOVA). Individual time points are compared using Student’s t-test with Bonferroni-Holm correction. **P, Q.** Heatmaps summarizing the success rate (**P**) or global d’ (**Q**) difference between WT and PS19 mice by sex. Color represents the mean difference between WT and PS19 values averaged across days 1–10 in **N** and **O**, respectively. Unpaired Student’s t-test. *p ≤ 0.05, **p ≤ 0.01, ***p ≤ 0.001. In violin plots, horizontal line represents mean, white circle represents median, and whiskers represent interquartile range. Error bars in line plots represent mean ± sem. Statistical details can be found in Table S1. See also Figs. S1, S2.

To allow long-term monitoring of calcium activity during spatial learning, we crossed PS19 mice^58^ with Thy1-GCaMP6f transgenic mice (GP5.3) to drive stable expression of the calcium indicator GCaMP6f in excitatory neurons in MEC layer 2^66–68^, a region particularly vulnerable in AD^69,70^. F1 offspring expressing both P301S tau and GCaMP6f were designated as “PS19 mice” and littermates expressing GCaMP6f but not P301S tau were WT controls. Young PS19 (up to 6 months old) and WT mice displayed low immunoreactivity to the AT8 antibody (Figs. 1A–C), which recognizes tau phosphorylated at serine 202 and threonine 205^71^ (pTau), an early pathological tau species that is strongly enriched in NFTs^72^. In contrast, immunoreactivity strongly increased in older PS19 mice (> 6 months) (Figs. 1A–C), consistent with reported NFT emergence around 5 months of age^58^. Given the well-documented sex differences in patients with AD and tauopathy models^73–77^, we examined whether overall pTau accumulation varied between male and female mice. Although no significant differences were observed, females exhibited a trend toward broader pTau spread and but lower local pTau intensity (Figs. 1D, E).

We next examined pTau accumulation in stellate and pyramidal cells, which are the primary excitatory cell types in MEC layer 2 and differ in their circuit connectivity, contributions to spatial navigation, and susceptibility in AD animal models and human patients^42,78–84^. Quantifying the overlap of pTau with reelin (a stellate cell marker) and calbindin (a pyramidal cell marker), we found that pyramidal cells were more often pTau+ than stellate cells (Figs. 1F, G), and pTau+ cells were more likely to be pyramidal than stellate cells (Fig. S1A). This pyramidal enrichment was evident in both sexes (Figs. 1H, S1B–D), but pTau intensity within the overlapping pyramidal cells was higher in males than females (Fig. 1I), consistent with higher local pTau abundance in males (Fig.1E). These outcomes were summarized in 2-by-2 heatmaps by cell type and sex (Figs. 1J, K). Overall, these results suggest that pyramidal cells are a particular target of tau pathology in MEC layer 2 of PS19 mice, especially in males.

To investigate MEC neural dynamics underlying spatial memory of PS19 mice, we used mice at ages showing spatial memory deficits and pTau accumulation but preceding the hindlimb paralysis and neurodegeneration reported in this line^58,63^. Hindlimb paralysis could impair running behavior during navigation, whereas neurodegeneration could confound cell-autonomous effects of pTau on neural function by disrupting network connectivity. Previous studies indicate that PS19 mice develop spatial memory deficits at approximately 6–8 months of age, when mobility remains largely intact^60,62–65^. In our F1 mice, significant pTau accumulation began after 6 months (Figs. 1B–D) and the median age of paralysis onset was 11.6 months (Fig. S1E). Accordingly, all experiments were conducted in mice aged 7–10 months, prior to paralysis onset (Fig. S1F). Quantification of total number of stellate and pyramidal cells per area revealed no significant neurodegeneration at these ages overall (Fig. S1G) or by sex (Fig. S1H).

Thus, all subsequent experiments used 7–10-month-old mice. At these ages, PS19 mice show robust pTau accumulation, particularly in layer 2 pyramidal cells, without significant paralysis or neuronal loss. The robust colocalization between pyramidal cells and pTau has also been observed in human brains in early stages of AD^83^, prompting us to investigate the function of these neurons in contrast to stellate cells.

### Male PS19 mice show behavioral deficits in an operant spatial learning task

To directly investigate the effects of tauopathy on MEC activity during spatial learning, we performed cellular-resolution two-photon calcium imaging in layer 2 of the MEC in 21 head-fixed mice (10 WT, 11 PS19) during VR navigation (Fig. 1L), enabling longitudinal tracking of hundreds of neurons per genotype group across multiple days of learning (Fig. 1L). Water-restricted mice were trained to navigate a 6-meter familiar environment (FE) and stop in an unmarked 50-cm reward zone to trigger a water reward delivery (Fig. 1M). After learning the task, mice were imaged for one day in the FE and for 10 consecutive days while learning to navigate a novel 6-meter environment (NE) with new visual cues and a water reward at distinct locations compared to the FE (Fig. 1M). During the FE training, PS19 mice experienced a similar number of training days (Fig. S1I) and traversed more laps than WT mice (Fig. S1J), demonstrating at least as much opportunity to learn the task. To ensure comparable NE experience, NE sessions were limited to 20 laps per day (Figs. S1I, J).

Learning was quantified by success rate, the percentage of laps rewarded. PS19 mice exhibited significantly lower success rates than WT mice during spatial learning (Figs. 1N, P). When analyzed by sex, PS19 males showed marked learning deficits, whereas PS19 females showed a mild, nonsignificant trend toward impairment (Figs. 1N, P). WT mice also learned faster (learning rate constant, WT_*K*_ or PS19_*K*_) and reached 95% of plateau earlier (WT_95%_ or PS19_95%_) than PS19 mice (Fig. S2A). To test whether mice were obtaining reward through indiscriminate stopping, we quantified the discrimination between the reward zone and other track locations using global d’^85,86^, with global d’>1 indicating successful discrimination^85–87^. Both WT and PS19 mice eventually exceeded this threshold, but WT mice displayed higher overall discrimination (Figs. 1O, Q) and reached the threshold sooner (Fig. S2B). The discrimination deficit was significant in PS19 males but not females (Figs. 1O, Q), while both sexes showed a trend of delayed rise to the threshold (Fig. S2B). Success rate and global d’ were also highly correlated (Fig. S2C), further arguing against an indiscriminate stopping strategy.

Because hyperlocomotion (i.e., elevated locomotor activity) has been reported in PS19 and other tauopathy and amyloid-β mouse models^60,88–90^, we tested whether impaired stopping could explain their spatial learning deficits. PS19 mice made similar numbers of stops as WT mice (Fig. S2D), indicating intact stopping, and still showed fewer stop attempts in the reward zone even under lenient stopping criteria (expanded reward zone, increased speed threshold, and shortened time to define a reward-zone stop) (Fig. S2E). Finally, while PS19 mice displayed a nonsignificant trend toward faster movement (Fig. S2F), when a subset of WT and PS19 mice were matched by their velocity on a per-day basis (Fig. S2G), PS19 mice still exhibited significantly lower success rate and global d’ than WT mice, especially among males (Figs. S2H, I).

In summary, PS19 mice exhibit significant spatial learning impairments, characterized by lower success rates and weaker reward-zone discrimination. This deficit was particularly pronounced in male mice, while PS19 females showed only a non-significant trend toward impairment but reached peak success more slowly than WT females. These learning impairments were not explained by stopping deficits or hyperlocomotion, suggesting that disrupted neuronal activity likely underlies the spatial learning deficits in PS19 mice.

### MEC cells of PS19 mice show hyper- or hypoactivity depending on molecular identity and sex

**Figure 2:**
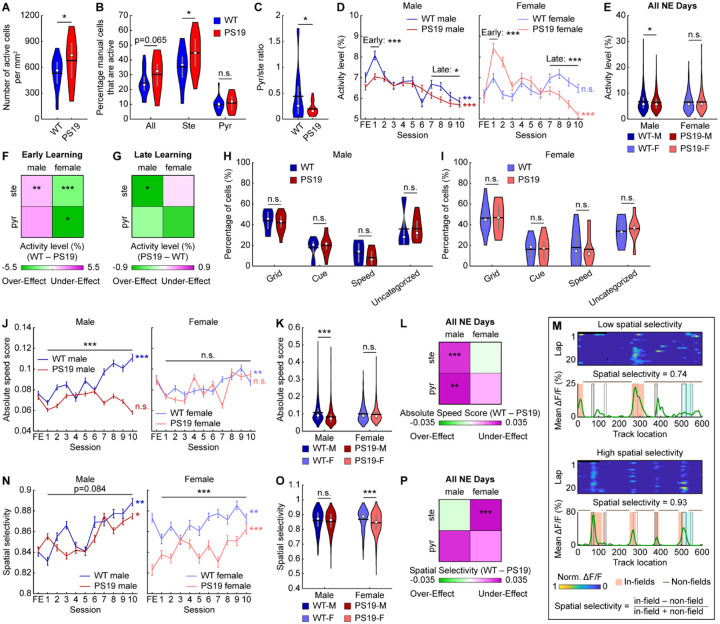
The MEC of PS19 mice exhibit altered neural activity level, speed modulation, and spatial selectivity **A.** The number of active cells per area on the FE day. **B.** The percentage of all manually outlined cells and manually outlined stellate (ste) and pyramidal (pyr) cells that are active on the FE day. **C.** The ratio between the fraction of active cells that are identified as pyramidal cells and as stellate cells. A low value indicates stellate cell enrichment. **D.** Activity level across learning in male (left) and female (right) mice. **E.** Activity level averaged across all NE days. WT-M: WT Male, PS19-M: PS19 Male, WT-F: WT Female, PS19-F: PS19 Female **F, G.** Heatmaps summarizing the activity level difference between WT and PS19 mice by sex and cell type. Color represents the mean difference between WT and PS19 values averaged across early (day 1, **F**) and late (days 7–10, **G**) learning. Unpaired Student’s t-test. **H, I.** The percentage of common cells identified as grid, cue, speed, or unclassified cells in male (**H**) and female (**I**) mice. **J.** Absolute speed score across learning in male (left) and female (right) mice. **K.** Absolute speed score averaged across all NE days. **L.** Heatmap summarizing the absolute speed score difference between WT and PS19 mice by sex and cell type. Color represents the mean difference between WT and PS19 values averaged across all learning days as in Fig. S4D. Unpaired Student’s t-test. **M.** Schematic of spatial selectivity calculation. Top: Calcium activity of example cells as a function of lap and track location with low (top) and high (bottom) spatial selectivity. Bottom: spatial selectivity calculation for the above cells based on activity level across laps as a function of track location. Black and grey rectangles represent left and right cues, respectively. Blue rectangle represents reward zone. **N.** Spatial selectivity across learning in male (left) and female (right) mice. **O.** Spatial selectivity averaged across all NE days. **P.** Heatmap summarizing the spatial selectivity difference between WT and PS19 mice by sex and cell type. Color represents the mean difference between WT and PS19 values averaged across all learning days as in Fig. S4H. Unpaired Student’s t-test. *p ≤ 0.05, **p ≤ 0.01, ***p ≤ 0.001. In violin plots, horizontal line represents mean, white circle represents median, and whiskers represent interquartile range. Violin plots use unpaired Student’s t-test. Error bars in line plots represent mean ± sem. In line plots, horizontal gray bars indicate p values for the group difference (early learning: unpaired Student’s t-test, late learning: two-way repeated measures ANOVA, all learning days: linear mixed-effects model), and p-values to the right indicate significant Pearson correlation of the mean value with respect to time. Statistical details can be found in Table S1. See also Figs. S3, S4.

We first compared the molecular identity (stellate vs. pyramidal) of active cells in MEC layer 2 of PS19 and WT mice. For the FE day, GCaMP6f+ stellate and pyramidal cells were identified by soma size inferred from GCaMP6f fluorescence^68,91^ (Figs. S3A–D). Compared to WT mice, PS19 mice had more active cells per square millimeter (Fig. 2A), a trend toward a higher fraction of active cells—particularly stellate cells— (Fig. 2B), and a lower pyramidal-to-stellate ratio among active cells (Fig. 2C). These trends were similar across sexes (Figs. S3E–H). Thus, PS19 mice showed a trend toward MEC hyperactivity driven by increased active cell numbers and stellate cell enrichment.

We further examined whether single-cell activity levels differed by calculating the daily average temporal activity (mean ΔF/F) in common cells (neurons active across all 11 imaging days: 1 FE day and 10 NE days). Because common cell activity is strongly associated with spatial learning^49^, these cells likely contribute to spatial memory formation. In most groups, activity increased sharply on NE day 1 and then gradually declined across learning days. In contrast, PS19 males showed only a modest increase on day 1 and lower activity than WT males on subsequent days, resulting in an overall reduced activity level (Figs. 2D, E). PS19 females, however, exhibited a larger activity increase in early learning (day 1) followed by lower activity on late learning days (days 7–10) (Fig. 2D), yielding comparable overall activity to WT females (Fig. 2E). These patterns were largely consistent across stellate and pyramidal cells, although cells in PS19 females showed mixed trends relative to WT during later learning (Figs. S3I, J). Stellate cells in male and female PS19 mice showed strong trends of overall hypoactivity and hyperactivity, respectively, with weaker trends in pyramidal cells (Fig. S3K).

To relate activity differences to the cell-type- and sex-specific tau accumulation pattern (Figs. 1J, K), we summarized molecular cell-type- and sex-specific activity level differences between PS19 and WT mice using 2×2 heatmaps for early and late learning (Figs. 2F, G). Green and magenta indicate over- and under-effects in PS19 mice, defined as activity exceeding or failing to reach the expected WT activity trend, respectively. Because WT activity increased on day 1 (Figs. 2D, S3I, J)^49^, lower and higher PS19 activity on day 1 relative to WT mice indicated under- and over-effects, respectively (Fig. 2F). During late learning, when WT activity declined (Figs. 2D, S3I, J), lower and higher PS19 activity indicates over- and under-effects, respectively (Fig. 2G). Importantly, both over- and under-effects could lead to cognitive deficits, as they indicate activity deviations from the WT level. Subsequent 2×2 heatmaps were generated using the same definitions.

In summary, PS19 mice tend to have more active cells in the MEC, with stellate cell enrichment relative to WT mice. Given the greater tau accumulation in pyramidal cells, stellate cell enrichment may reflect compensation for pyramidal dysfunction. However, mean activity levels differ from WT in a cell-type-, sex-, and learning-stage-dependent manner.

### MEC neurons of PS19 mice have altered speed modulation and spatial selectivity

We further investigated functional cell type composition of common cells and their activity changes with respect to speed and spatial location, key regulators of MEC neural activity^32,34,37,38^. We classified grid cells, neurons active in a triangular lattice pattern in a two-dimensional (2D) space^32^, during the navigation of our one-dimensional (1D) VR tracks^68,92,93^. We also classified cue cells, which fire around visual landmarks^36^, and speed cells, whose activity is significantly correlated with mouse velocity^37,38^. The common cell population of each functional cell type was those cells that met the classification criteria on ≥6 imaging days (more than half of the days)^49^. The remaining cells were considered unclassified. Functional cell type proportions were largely similar across genotypes in both sexes (Figs. 2H, I). The most notable difference was a trend toward fewer speed cells in PS19 males (Figs. 2H), suggesting reduced speed modulation.

To directly measure speed modulation, we calculated speed scores, the correlation between single-cell temporal activity and velocity^37,38^. In WT males, raw speed scores remained relatively stable across learning (Fig. S4A) because both positive and negative speed scores gradually increased in magnitude (Figs. S4B, C). This pattern was absent in PS19 males, which showed no magnitude increases (Figs. S4B, C) and lower raw and signed speed scores (Figs. S4A–C), particularly for positively modulated cells. In contrast, WT females showed decreasing negative speed scores (i.e., more negatively modulated activity by speed), leading to decreasing raw speed scores (Figs. S4A–C). PS19 females followed the same trends (Figs. S4A–C). Absolute speed scores, which reflect modulation strength independent of direction, tended to increase across learning in WT mice of both sexes and in PS19 females, but not in PS19 males (Fig. 2J). PS19 males also exhibited significantly lower absolute scores than WT males (Figs. 2J, K), including in stellate and pyramidal cells (Figs. 2L, S4D). To assess functional cell-type differences, we analyzed common grid cells versus non-grid cells (all other common cells) and found lower absolute speed scores in PS19 males than WT males in both cell types (Fig. S4E). Because absolute speed scores rise during learning in WT mice (Fig. 2J), reduced speed modulation in PS19 males represents an activity deficit.

Furthermore, we assessed spatial selectivity because many cells exhibited spatial fields, clusters of adjacent spatial bins with activity above chance (Fig. 2M). Spatial selectivity was quantified by in-field versus non-field activity (Fig. 2M). Male PS19 mice exhibited similar selectivity to WT mice, whereas PS19 females showed lower selectivity (Figs. 2N, O), primarily due to elevated non-field activity that outweighed their slightly higher in-field activity (Figs. S4F, G). This reduction was largely attributable to stellate rather than pyramidal cells (Figs. 2P, S4H) and was present in both grid and non-grid cells (Fig. S4I). Because spatial selectivity increased with learning (Fig. 2N), lower spatial selectivity in PS19 females likely represents an activity deficit.

Together, these results revealed activity differences between PS19 and WT mice that vary by molecular and functional cell types. The diminished speed encoding in males and the reduced spatial selectivity in female PS19 mice may underlie their impairments in spatial learning.

### Male PS19 mice have impaired MEC within-day and cross-day activity consistency

**Figure 3:**
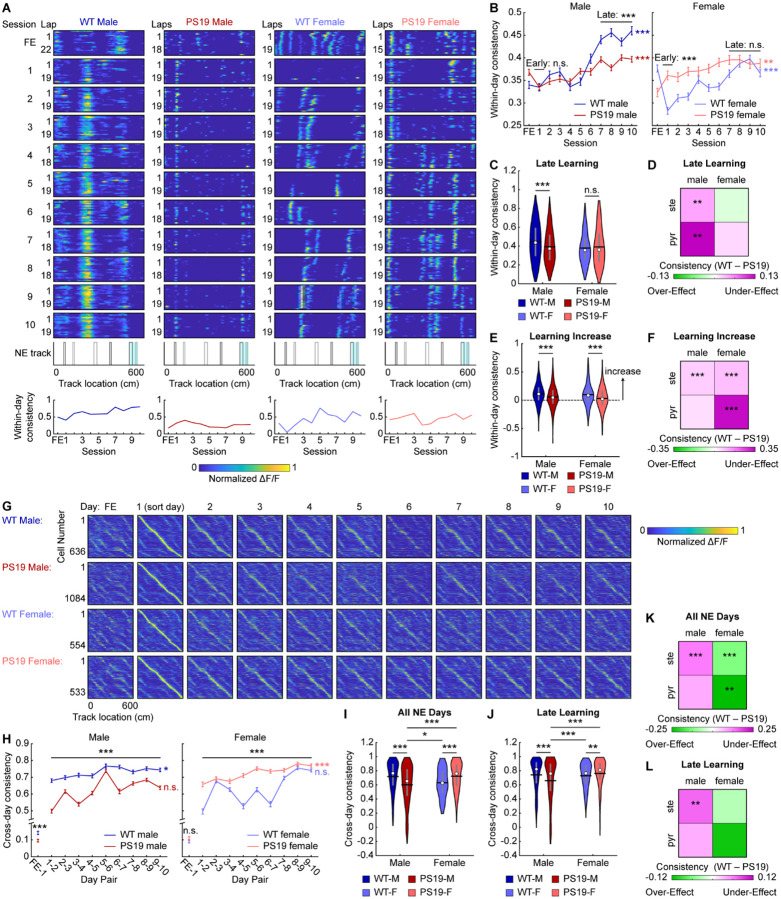
Male PS19 mice have impaired MEC within-day and cross-day activity consistency **A.** Top: Calcium activity matrices of example cells as a function of lap and track location for the FE day and all 10 NE days for each sex/genotype category (from left to right: WT male, PS19 male, WT female, PS19 female). Middle: Environment template. Black and grey rectangles represent left and right cues, respectively. Blue rectangle represents reward zone. Bottom: Within-day activity consistency for the example cells. **B.** Within-day activity consistency across learning in male (left) and female (right) mice. Horizontal gray bars indicate p values for the group difference (early learning: unpaired Student’s t-test, late learning: linear mixed-effects model). P-values to the right indicate significant Pearson correlation of the mean value with respect to time. **C.** Within-day activity consistency averaged across late learning (days 7–10). Unpaired Student’s t-test. WT-M: WT Male, PS19-M: PS19 Male, WT-F: WT Female, PS19-F: PS19 Female **D.** Heatmap summarizing the within-day activity consistency difference between WT and PS19 mice by sex and cell type. Color represents the mean difference between WT and PS19 values averaged across late learning as in Fig. S5C. Unpaired Student’s t-test. **E.** The increase in within-day activity consistency between early learning (day 1) and late learning. Unpaired Student’s t-test. **F.** Heatmap summarizing the within-day activity consistency difference between WT and PS19 mice by sex and cell type. Color represents the mean difference between WT and PS19 values for the increase between late and early learning as in Fig. S5F. Unpaired Student’s t-test. **G.** Activity matrices for the FE day and all 10 NE days for each sex/genotype category (from top to bottom: WT male, PS19 male, WT female, PS19 female). The normalized mean activity of individual cells (each row of an activity matrix) were sorted based on the peak locations on day 1. **H.** Cross-day activity consistency across learning in male (left) and female (right) mice. Horizontal gray bars indicate p values for the group difference (linear mixed-effects model). FE-1 comparison uses unpaired Student’s t-test. P-values to the right indicate significant Pearson correlation of the mean values with respect to time. **I, J.** Cross-day activity consistency averaged across all learning days (**I**) and late learning (**J**). Unpaired Student’s t-test with Bonferroni-Holm correction. **K, L.** Heatmaps summarizing the cross-day activity consistency difference between WT and PS19 mice by sex and cell type. Color represents the mean difference between WT and PS19 values averaged across all learning days (**K**) or late learning days (**L**) as in Fig. S5I and Fig. S5J, respectively. Unpaired Student’s t-test. *p ≤ 0.05, **p ≤ 0.01, ***p ≤ 0.001. In violin plots, horizontal line represents mean, white circle represents median, and whiskers represent interquartile range. Error bars in line plots represent mean ± sem. Statistical details can be found in Table S1. See also Fig. S5.

Spatially consistent MEC activity is essential for successful spatial learning^49^. To assess whether PS19 mice exhibit deficits in spatial activity consistency, we first quantified within-day consistency, the average correlation between each lap’s spatial activity and all other laps within the same day (Fig. 3A).

During early learning, male PS19 mice showed within-day consistency comparable to WT mice (Fig. 3B), but by the late learning phase, their consistency was significantly lower (Figs. 3B, C), indicating a failure to stabilize their activity spatial maps over time. While lower consistency was present in both stellate and pyramidal cells, the late-learning difference was most pronounced in pyramidal cells (Figs. 3D, S5A–C), indicating a cell-type-specific deficit. Notably, this consistency difference was similar across grid and non-grid cells (Figs. S5D, E). In contrast, PS19 female mice exhibited higher within-day consistency than WT females during early learning overall and across both stellate and pyramidal cells, with differences diminished by late learning (Figs. 3B–D, S5A–C). Interestingly, this effect was stronger in grid than non-grid cells (Figs. S5D, E). Thus, impaired late-learning within-day map stability was a specific deficit to male PS19 mice.

Previous work showed that learning-related increases in within-day consistency accompany robust behavioral improvement, highlighting the importance of flexible updating spatial maps during successful learning^49^. However, both PS19 male and female mice exhibited smaller increases in within-day consistency across learning than WT mice (Figs. 3B, E), a pattern observed in both stellate and pyramidal cells (Figs. 3F, S5A, B, F). This restricted dynamic range indicates reduced flexibility of within-day spatial representations in PS19 mice despite repeated environmental exposure.

We next examined cross-day activity consistency, defined as the correlation of each cell’s lap-averaged spatial activity between adjacent days (Fig. 3G). Consistency between FE and NE day 1 was near-zero for all groups, with PS19 males exhibiting even lower values, indicating preserved remapping ability in PS19 mice (Fig. 3H). Across learning, male PS19 mice showed lower consistency than WT males (Figs. 3H, I). In contrast, similar to the within-day consistency, PS19 females had higher early-learning cross-day consistency than WT females, which converged by late learning (Fig. 3H). By days 7–10, WT males, WT females, and PS19 females reached high cross-day consistency, whereas PS19 males remained significantly lower (Figs. 3H, J). These differences were consistent across molecular and functional cell types (Figs. 3K, L, S5G–L), indicating a global deficit in cross-day consistency of PS19 males.

Together, these results show that PS19 mice exhibit impaired spatial activity consistency during learning. Male PS19 mice failed to maintain stable spatial maps both within and across days, with the within-day deficit most pronounced in pyramidal cells. Moreover, both sexes displayed inflexibility in updating spatial representations with increased experience, which may lead to inefficient learning. The more severe deficits in male PS19 mice likely contribute to their pronounced spatial learning impairments.

### Male PS19 mice do not develop a cognitive map shared by other mouse groups

The above results revealed that male PS19 mice exhibited more severely impaired cross-day activity consistency than WT males, WT females, or PS19 females. To further investigate this deficit, we considered two possibilities. First, PS19 males may have failed to develop a common “correct” cognitive map shared by other groups. Alternatively, rather than converging on a common map, each animal might develop an individual cognitive map whose internal consistency, rather than its specific pattern, supports successful spatial learning.

**Figure 4:**
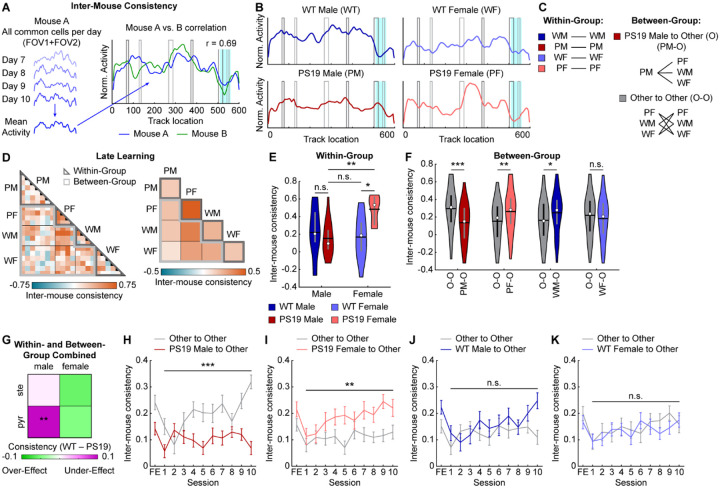
Male PS19 mice do not develop a cognitive map shared by other mouse groups **A.** Schematic of late-learning (NE days 7–10) inter-mouse consistency calculation. **B.** Group averages of late-learning spatial activity maps as calculated in **A**. **A, B.** Black and grey rectangles represent left and right cues, respectively. Blue rectangle represents reward zone. **C.** Schematic for within-group (left) and between-group (right) comparisons. **D.** Left: Pairwise inter-mouse consistency sorted by genotype/sex group. Right: Average inter-mouse consistency for each group-to-group block. **E.** Pooled inter-mouse consistency for pairwise within-group comparisons. Unpaired Student’s t-test with Bonferroni-Holm correction. **F.** Pooled inter-mouse consistency for pairwise between-group comparisons with each genotype/sex group serving as the reference group as indicated. The Unpaired Student’s t-test. **G.** Heatmap summarizing the inter-mouse consistency difference between WT and PS19 mice by sex and cell type. Color represents the mean difference between WT and PS19 values from Fig. S6B, C. All pairwise correlations between mice in a given group with all mice were pooled for calculating the difference. **H-K.** Inter-mouse consistency for between-group comparisons across learning using PS19 males (**H**), PS19 females (**I**), WT males (**J**), and WT females (**K**) as a reference group. Horizontal gray bars indicate p values for the group difference (linear mixed-effects model). *p ≤ 0.05, **p ≤ 0.01, ***p ≤ 0.001. In violin plots, horizontal line represents mean, white circle represents median, and whiskers represent interquartile range. Error bars in line plots represent mean ± sem. Statistical details can be found in Table S1. See also Fig. S6.

To distinguish these possibilities, we correlated spatial maps between individual mice (inter-mouse consistency) (Fig. 4A). For each mouse, a late-learning map representing the outcome of NE learning was generated by averaging the spatial activity of all common cells across days 7 to 10 (Figs. 4A, B, S6A). Correlations were then computed within the same sex/genotype group (within-group comparisons) or between different groups (between-group comparisons) (Figs. 4A, C). Within-group comparisons showed that PS19 males—whether using all common cells, pyramidal cells, or stellate cells—exhibited variability similar to WT groups, whereas PS19 females had more consistent maps (Figs. 4D, E, S6B–E). Thus, within-group variation does not account for the learning differences observed between PS19 males and other groups.

In contrast, PS19 male maps were less similar to those of other groups. Between-group comparisons using common cell maps revealed significantly lower PS19-male-to-Other consistency compared with Other-to-Other consistency, indicating that PS19 male maps differed from all other groups (Figs. 4D, F). This effect was specific to pyramidal but not stellate cells (Figs. S6B, C, F, G) and was absent in other sex/genotype groups (Figs. 4D, F, S6B, C, F, G). When combining all within- and between-group comparisons, only pyramidal cells in PS19 males showed significantly lower inter-mouse consistency relative to WT mice (Fig. 4G). These findings suggest that while other mice converged on a common cognitive map by the end of learning, PS19 males developed distinct maps, with deficits primarily affecting pyramidal cells.

We next asked whether the cognitive map differences in PS19 males were present at the onset of learning or emerged gradually. To address this, we tracked map development across days by calculating inter-mouse consistency between groups on each learning day. While PS19-male-to-Other consistency remained low, Other-to-Other consistency steadily increased over learning and diverged progressively from PS19-male-to-Other consistency (Fig. 4H). This pattern was not observed when substituting any other sex/genotype group for PS19 males (Figs. 4I–K). These findings suggest that although all groups began with similarly variable maps, PS19 males uniquely failed to develop the shared cognitive map that emerged across other groups during learning.

In summary, successful spatial learning requires both high internal consistency within each animal’s cognitive map and convergence toward a common, “correct” map. The failure of PS19 males to form this shared map, primarily in pyramidal cells, likely contributes to their spatial learning deficits, underscoring the critical role of proper cognitive map formation in spatial memory.

### Spatial activity of male PS19 mice is anchored to visual cues

**Figure 5:**
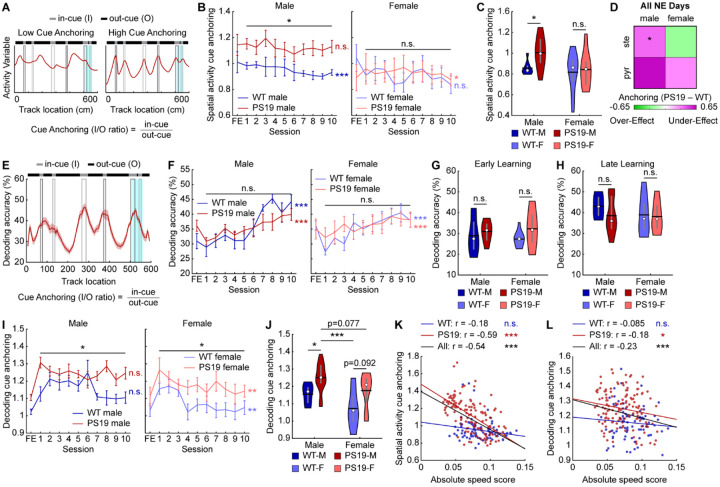
Spatial activity of male PS19 mice is anchored to visual cues **A.** Examples of spatial activity with low (left) and high (right) cue anchoring and the cue anchoring calculation. Vertical black and grey rectangles represent left and right cues, respectively. Blue rectangle represents reward zone. **B.** Spatial activity cue anchoring across learning in male (left) and female (right) mice. **C.** Spatial activity cue anchoring averaged across all learning days. WT-M: WT Male, PS19-M: PS19 Male, WT-F: WT Female, PS19-F: PS19 Female **D.** Heatmap summarizing the spatial activity cue anchoring difference between PS19 and WT mice by sex and cell type. Color represents the mean difference between PS19 and WT values averaged across all learning days as in Fig. S7F. Unpaired Student’s t-test. **E.** Example of decoding accuracy as a function of track position. From PS19 males averaged across the 10 learning days. Vertical black and grey rectangles represent left and right cues, respectively. Blue rectangle represents reward zone. **F.** Full track decoding accuracy across learning in male (left) and female (right) mice. **G, H.** Full track decoding accuracy averaged across early (day 1, **G**) and late learning (days 7–10, **H**). **I.** Decoding cue anchoring across learning in male (left) and female (right) mice. **J.** Decoding cue anchoring averaged across all learning days. Unpaired Student’s t-test with Bonferroni-Holm correction. **K, L.** Correlation between absolute speed score and spatial activity cue anchoring (**K**) or decoding cue anchoring (**L**) in male mice. Each point represents the activity from one FOV from one session. Pearson correlation. *p ≤ 0.05, **p ≤ 0.01, ***p ≤ 0.001. In violin plots, horizontal line represents mean, white circle represents median, and whiskers represent interquartile range. Violin plots use unpaired Student’s t-test. Error bars and shaded regions in line plots represent mean ± sem. In line plots, horizontal gray bars indicate p values for the group difference (linear mixed-effects model), and p-values to the right indicate significant Pearson correlation of the mean value with respect to time. Statistical details can be found in Table S1. See also Figs. S7, S8.

Following the above finding, we asked what kind of map PS19 males formed during learning and how it differed from other groups. The impaired speed modulation observed in PS19 males (Figs. 2J–L) and low spatial activity consistency (Figs. 3B–D) suggest disrupted path integration, whereby self-motion cues (e.g., running speed and acceleration) are integrated over time to estimate position independently of external landmarks^40,94–96^. Disruption of this process would bias spatial representations toward cue-rich regions and weaken representations in cue-lacking regions^54,97^ (cue anchoring; Fig. 5A). Consistent with this prediction, we observed a visual trend toward cue anchoring in PS19 males in both group-averaged maps (Fig. 4B) and individual late-learning maps (Fig. S6A).

To quantify cue anchoring, we calculated the ratio of activity in in-cue (I) versus out-cue (O) regions (spatial activity cue anchoring) (Fig. 5A). PS19 males showed a trend of higher in-cue activity but similar out-cue activity compared to WT males (Figs. S7A–C), resulting in significantly higher cue anchoring across learning (Figs. 5B, C). In contrast, PS19 females exhibited insignificant trends of elevated activity in both in-cue and out-cue regions (Figs. S7A–C), yielding comparable cue anchoring to WT females (Figs. 5B, C). Notably, cue anchoring in WT males decreased across learning (Fig. 5B), suggesting a shift toward a more global spatial representation that might support successful learning. However, cue anchoring of PS19 males failed to improve, suggesting a deficit. When split by molecular cell type, pyramidal cells of male PS19 mice showed the highest cue anchoring (Figs. S7D–F) and the largest difference from WT males (Fig. 5D). For functional cell types, both grid and non-grid cells in male PS19 mice showed a trend of stronger cue anchoring than WT males (Figs. S7G–I).

We further validated activity cue anchoring using population decoding of track positions^49,98^. A decoder built from population activity in odd laps at individual 5-cm spatial bins was used to predict positions from even-lap activity, with accuracy defined as the percentage of correctly decoded positions within ±2 bins from the original bin. Because decoding accuracy is cell-number dependent^49,98^, decoding was performed with 100 random samples of 30 cells for each field-of-view (FOV). All cells were included because many FOVs lacked sufficient numbers of common, stellate, or pyramidal cells for separate analyses.

Decoding accuracy was assessed across the full track and by location (Fig. 5E). Consistent with our previous work^68^, WT mice exhibited gradually improved accuracy during learning (Fig. 5F). PS19 males exhibited lower late-learning accuracy than WT males, while female WT and PS19 mice reached similar levels (Figs. 5F–H). Both PS19 males and females exhibited trends of higher accuracy at in-cue regions than WT mice during early learning and lower accuracy at out-cue regions during late learning (Figs. S7J–L), resulting in higher ratios of decoding accuracy at in-cue versus out-cue regions (decoding cue anchoring) throughout learning (Figs. 5E, I, J). Notably, PS19 males showed the highest anchoring among all groups (Fig. 5J). Cue anchoring was more pronounced in grid than non-grid cells (Figs. S7M–O). Across learning, both in-cue and out-cue decoding improved (Figs. S7J, K), but cue anchoring declined (Fig. 5I), supporting the idea that reduced cue anchoring favors successful spatial learning and indicating that elevated anchoring in PS19 mice reflects an activity deficit.

Together, these results demonstrate that PS19 mice, especially males, exhibited pronounced cue anchoring in MEC spatial representations. Cue anchoring was stronger in grid than non-grid cells and in pyramidal than stellate cells. The relatively weak representation of non-cue regions could stem from reduced speed cell number and activity speed modulation (Figs. 2H–L), both of which are essential for providing self-motion signal during path integration when external landmarks are unavailable^6,99^. This connection is supported by a strong negative correlation between absolute speed score and cue anchoring in PS19, but not WT, males (Figs. 5K, L), indicating that poor speed modulation is robustly associated with higher cue anchoring specifically under conditions of behavioral impairment. This relationship was observed across cell types but was strongest in pyramidal and non-grid cells (Figs. S8A–F). Thus, impaired self-motion integration in PS19 males likely drives excessive reliance on cue regions rather than the formation of a coherent, global cognitive map. These deficits are potentially manifested as path integration deficits, which have been observed in PS19 mice and are associated with pTau accumulation in the EC^61^, ultimately leading to disrupted spatial memory formation.

### Tau accumulation and activity deficits in male PS19 mice predominantly occur in pyramidal cells

**Figure 6:**
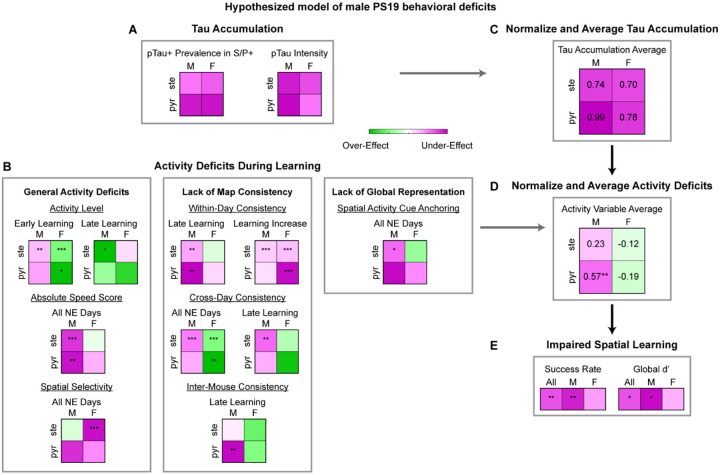
Tau accumulation and activity deficits in male PS19 mice predominantly occur in pyramidal cells **A-E.** Summary of potential mechanism explaining PS19 behavioral deficits, whereby tau accumulation, particularly in pyramidal cells, leads to changes in MEC activity, which leads to impaired spatial learning. **A, B, E.** All heatmaps summarizing the sex (male, M vs. female, F) and cell type (stellate, ste, S vs. pyramidal, pyr, P) split of tau accumulation (**A**), activity deficits (**B**), and behavioral deficits (**E**) are identical to those presented in earlier figures. **C, D.** Normalized and averaged 2×2 heatmap of tau accumulation (**C**) and activity deficits (**D**) showing overall tau accumulation and activity deficits are most prominent in pyramidal cells of male PS19 mice. Individual activity heatmaps were normalized to their maximum absolute value, so the individual heatmap values and the color range for the average heatmaps range between −1 and 1. One-sample Student’s t-test for difference from 0. Note that the average heatmaps should only be interpreted qualitatively. *p ≤ 0.05, **p ≤ 0.01, ***p ≤ 0.001. Statistical details can be found in Table S1. See also Fig. S9.

Overall, PS19 mice, particularly males, exhibited widespread deficits across multiple activity domains, including general activity features (activity level, absolute speed score, and spatial selectivity), map consistency (within-day consistency, cross-day consistency, and inter-mouse consistency), and global spatial representation (spatial activity cue anchoring and decoding cue anchoring) (Fig. S9). Given the stronger pTau accumulation in pyramidal cells than in stellate cells (Figs. 1, S1, summarized in Fig. 6A) and the differences in the activity deficits across these cell types, we examined whether the imbalance in pTau accumulation corresponds to the severity of activity deficits, a key step in linking tauopathy to spatial memory impairment.

To provide a qualitative measure of deficit severity, we summarized the 2×2 heatmaps capturing cell-type– and sex-specific differences between PS19 and WT mice across all activity measures (Figs. 2–5, S4–S7, summarized in Fig. 6B). Tau and activity heatmaps were normalized by their maximum absolute values, yielding values between −1 and 1, and averaged to generate summary heatmaps for tau accumulation and overall activity differences between WT and PS19 mice (Figs. 6C, D). PS19 males exhibited strong activity under-effects, particularly in pyramidal cells, reflecting pronounced activity deficits below WT levels (Fig. 6D), consistent with their higher pTau burden in pyramidal cells (Fig. 6C) and potentially leading to severe spatial learning impairments (Fig. 1, summarized in Fig. 6E). In contrast, PS19 females showed relatively mild over-effects, especially in pyramidal cells. This trend of exceeding of WT level may also contribute to cognitive disruption (e.g., hyperactivity^100^), albeit to a lesser degree, likely reflecting their lower pTau burden (Fig. 6C) and resulting in mildly impaired spatial learning (Figs. 6E).

Together, these findings suggest that selective tau accumulation in pyramidal cells is closely associated with activity dysfunction and spatial memory impairment, establishing a link between tauopathy and cognitive deficits.

### MEC activity changes are predictive of behavioral deficits in PS19 mice

While the above qualitative analyses suggest links between tau burden, MEC activity impairments, and spatial learning deficits, we further strengthen these relationships using quantitative modeling to identify which activity variables best predict learning performance and how cell types with different tau burdens contribute to these predictions.

**Figure 7:**
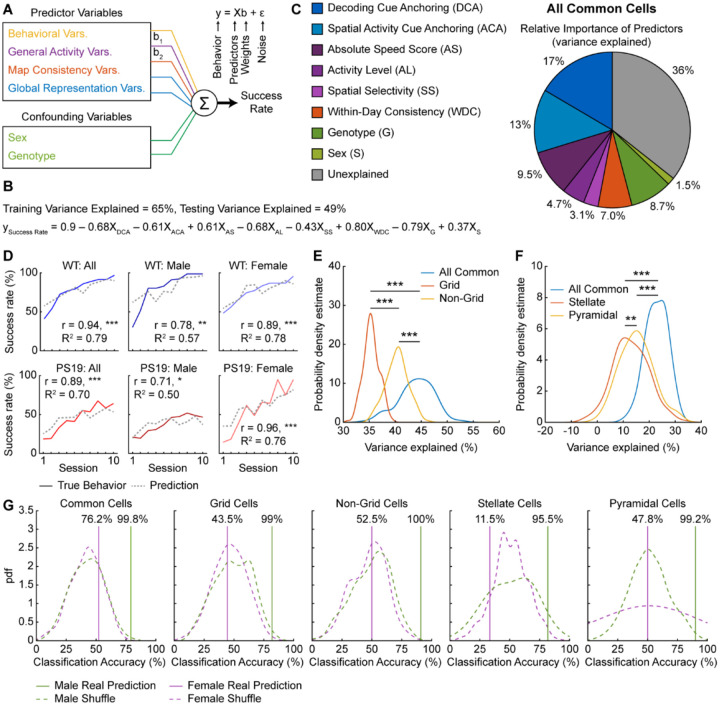
MEC activity changes are predictive of behavioral deficits in PS19 mice **A.** Schematic showing use of a general linear model (GLM) to predict behavior using behavioral, general activity, map consistency, global representation, and confounding variables. **B.** Final LOOVC model performance. Training and testing variance explained are calculated by pooling LOOCV iterations. Model equation is calculated by averaging coefficients from each iteration. Each X represents the value of a given predictor or confounding variable. **C.** Relative contribution of predictor and confounding variables to behavioral variance explained in a GLM using activity from all common cells. Colors correspond to variable categories in **A**. **D.** Comparison of mean success rate (colored lines) and model predictions (dashed grey lines) across learning for WT (top) and PS19 (bottom) in all (left), male (middle), and female (right) mice using activity from all common cells. Pooled test predictions across all LOOCV iterations were used to calculate prediction means. Pearson correlation. **E, F.** Probability distribution of variance explained for models (**E**: all-common-cell, grid cell, non-grid cell; **F**: all-common-cell, stellate cell, pyramidal cell) based on subsampled activity data. Each mouse was randomly subsampled 200 times, such that the activity variables of all three groups were based on identical numbers of cells. Unpaired Student’s t-test. **E**: common vs. grid: d (Cohen’s D) = 3.2. common vs. non-grid: d = 1.3. grid vs. non-grid: d = 2.7. **F**: common vs. stellate: d = 1.7. common vs. pyramidal: d = 1.4. stellate vs. pyramidal: d = 0.31. **G.** Classification accuracy of genotype based on modeling of mouse behavior from all common, grid, non-grid, stellate, and pyramidal cells (from left to right). Vertical line labels indicate the percentile of the real prediction classification among the shuffles. *p ≤ 0.05, **p ≤ 0.01, ***p ≤ 0.001. Statistical details can be found in Table S1. See also Figs. S10 and S11

We utilized a general linear model (GLM)^101^ to predict each mouse’s success rate in a given session based on behavior or activity variables within that session (Fig. 7A), using data from all NE days. Model predictions were evaluated by calculating the percentage of behavioral variance explained (R^2^), where 100% indicates a perfect match between predicted and true behaviors. Predictor variables included behavioral variables (mean velocity), general activity variables (activity level, raw speed score, absolute speed score, and spatial selectivity), map consistency variables (within-day consistency, cross-day consistency, and inter-mouse consistency), and global representation variables (spatial activity cue anchoring and decoding cue anchoring). Activity variables were derived from common cells, as described previously, except decoding cue anchoring, which used all cells. Sex and genotype were included as confounding variables. All predictor variables were standardized using z-score normalization and used in an initial model with the confounding variables. To identify the most informative predictors, variables that did not significantly contribute were iteratively removed while retaining confounding variables (Fig. S10A). This procedure substantially increased variance explained compared to shuffled predictions, where success rates were randomized to disrupt correlations with predictors^102^ (Figs. S10A, B).

The final model retained the six activity variables that were never removed across all iterations: activity level, absolute speed score, spatial selectivity, within-day consistency, spatial activity cue anchoring, and decoding cue anchoring (Figs. 7B, S11A). Individually removing any of these variables reduced variance explained, whereas adding back excluded variables had little effect, highlighting the importance of the final variables (Fig. S10C). Excluding sex and genotype during variable selection did not alter the final activity variables, indicating no bias from confounding variables (Fig. S10D). Correlation analyses confirmed that these activity variables were strongly associated with success rate (Figs. S10E–N), with four (absolute speed score, within-day consistency, decoding cue anchoring, spatial activity cue anchoring) significantly correlated within each genotype (Figs. S10F, J, M, N).

Dominance analysis quantified each variable’s contribution by measuring the effect of removing it^103^ (Fig. 7C). The six activity variables collectively explained 54% of the behavioral variance, while confounding variables accounted for only 10% (Fig. 7C), underscoring the strong link between MEC activity and learning. Predicted success rates closely matched the true learning curves across groups, showing significantly high correlations (r) and variance explained (R^2^) (Fig. 7D), though individual mice showed some variability (Fig. S11B).

Given that grid cells play a central role in MEC spatial coding^104–107^ and are disrupted in tauopathy, as reported previously^9,39,40^ and here, we tested whether their activity is particularly predictive of behavior by comparing grid, non-grid, and all common cells (including grid and non-grid cells together). Because using a greater number of cells may increase predictive ability^108^, we subsampled equal numbers of cells per category and repeated the prediction 200 times using the final model variables to generate distributions of variance explained. While subsampling slightly reduced variance explained, the all-common-cell model outperformed both grid and non-grid models, and non-grid cells explained more variance than grid cells (Fig. 7E). Effect sizes (Cohens d^109^) were large and only a few subsamples were needed to reach 80% power^109^ (80% probability of obtaining a significant result if a true difference exists) (Figs. 7E, S11C). Thus, both grid and non-grid cell activity contribute to predicting behavior, with non-grid activity being more informative.

Because male PS19 mice show greater activity impairment in pyramidal than stellate cells, we tested whether pyramidal activity better predicts spatial learning. Using all common cells and either stellate or pyramidal cells from the common cell population, we applied the same subsampling procedure to select equal numbers per category. The all-common-cell model explained more variance than either cell-type model, and pyramidal cells outperformed stellate cells (Fig. 7F). Effect sizes were large for common versus cell-type models and moderate for stellate versus pyramidal cells (Fig. 7F). Only few subsamples were needed to achieve 80% power for common versus cell-type comparisons, but more were required for stellate versus pyramidal cells (Fig. S11D). These results show that both cell types contribute to predicting learning, with pyramidal cells being more informative.

Finally, we tested whether these activity variables could classify mouse genotype. Using the final model variables—excluding sex and genotype as confounding variables and without subsampling—we averaged late-learning predictions for each mouse, which was further classified as WT or PS19 based on proximity of the prediction to group averages of real behaviors. Male classification exceeded chance across all cell types (all common cells, grid, non-grid, stellate, and pyramidal cells) (Fig. 7G), indicating that these variables captured PS19 behavioral deficits regardless of functional or molecular cell type. Female classification was at chance (Fig. 7G), likely reflecting minimal deficits of PS19 females.

In summary, MEC activity—including in grid, non-grid, stellate, and pyramidal cells—predicts spatial learning. Non-grid and pyramidal cells contributed to better prediction than grid and stellate cells, respectively. Altered activity patterns in PS19 mice—encompassing activity level, spatial selectivity, speed modulation, map consistency, and cue anchoring—likely underlie their spatial learning deficits, which is further supported by their ability to classify genotype.

## DISCUSSION:

The link between EC neural activity and spatial memory deficits in tau pathology in aging and early AD remains poorly understood. To investigate this, we performed two-photon calcium imaging of layer 2 excitatory neurons in the MEC of WT and PS19 mice as they learned to navigate a novel VR environment for 10 days. pTau accumulation in MEC layer 2 increased markedly after 6 months, particularly in pyramidal cells. Interestingly, despite largely comparable pTau accumulation across sexes, only PS19 males exhibited severe spatial learning deficits, whereas females were less affected. To identify underlying neural mechanisms, we quantified a broad set of behavioral and activity features and used modeling to predict learning performance and distinguish genotypes. Spatial learning deficits in PS19 mice were primarily associated with impaired speed modulation, reduced spatial map stability, and overrepresentation of cue-rich versus cue-poor regions. These observations suggest deficits in path integration, which could prevent the formation of a reliable and global cognitive map and ultimately lead to impaired spatial memory. The activity deficits were most robust in pyramidal cells, consistent with their greater pTau burden relative to stellate cells. Furthermore, learning performance was well predicted by MEC activity, with higher accuracy by non-grid than grid cells and by pyramidal than stellate cells. MEC activity also reliably distinguished male PS19 mice from WT controls. Together, these results strongly link tau pathology to MEC dysfunction and highlight specific neural activity features that likely drive spatial learning deficits. These features may inform future diagnostic biomarkers for early AD and guide therapeutic strategies for aging- and AD-related tau pathology by focusing research on EC circuit function.

### Choice of mouse models

Our study used the PS19 mouse model, which overexpresses the human 1N4R tau P301S mutant under the mouse prion promoter^58^. While this overexpression model does not fully recapitulate tau pathology in aging and early AD, it offers several advantages. First, high tau expression in MEC neurons induces robust activity changes, allowing us to examine the relationship between tau pathology and neuronal function. Second, PS19 mice develop spatial memory deficits early, before substantial neurodegeneration occurs. This temporal window allows more meaningful comparison with tau pathology in aging and early AD, and facilitates single-neuron analyses without major confounds from widespread network disruption. Third, memory impairments arise prior to paralysis, allowing mice to perform VR-based navigation tasks that require sustained locomotion. Finally, the absence of amyloid-β in this model may recapitulate aspects of human aging, as tau accumulation is almost universal at old age, even in the absence of amyloid-β^26^. Thus our study potentially offers insights into human aging not offered by standard mouse aging studies, as mice do not naturally develop tau accumulations in old age^110^. Although tau expression in P19 mice is not restricted to the MEC, we find that MEC pTau accumulation patterns strongly correlate with MEC neural activity features, which in turn predict spatial learning and reliably distinguishes PS19 from WT mice. Together, these findings establish a link between tau pathology, MEC dysfunction, and spatial memory deficits.

In contrast, other tauopathy models are less suitable for these goals. EC-tau mice^111–113^, expressing human P301L tau mutant selectively in entorhinal layer 2/3, show spatial memory deficits only at ~30 months^39,111^, with substantial neuronal loss beginning at 24 months^39,113^. These features limit VR navigation due to markedly reduced locomotion in aged mice^114^ and confound neural activity interpretation. In addition, the severe neuronal loss in this model compares poorly with the pathology observed in aging and early AD. Human MAPT knock-in (KI) mice, expressing human WT tau isoforms or mutant human tau^115–119^, avoid overexpression artifacts but often fail to develop robust tau pathology or spatial cognitive deficits even at advanced ages^116–118,120,121^. Robust phenotypes typically require combining multiple MAPT mutations^122,123^, crossing with Aβ/APP mouse models^119,124^, or seeding with human brain derived tau^125^. These manipulations complicate experiments using crosses with Thy1-GCaMP6 mice^67^, which ensure stable GCaMP6f expression for long-term monitoring of neural activity during learning but could alter the development or timing of tau-pathology-related phenotypes. Moreover, learning impairments in these models often coincide with severe synaptic loss and neurodegeneration^122,123^, again making them difficult to compare with aging and early AD and hindering efforts to isolate tau-induced changes in neural activity from the effects of synaptic or neuronal loss.

### Comparison with previous studies on the MEC and the hippocampus

Although many studies have reported a wide range of malfunctions of MEC neurons in AD and tauopathy models^39–42^, most have focused on neural activity at a single time point of navigation. Because spatial learning and memory depend on repeated exploration across days^47,48^, it has remained unclear which specific MEC activity features drive learning deficits. By longitudinally tracking MEC neural activity alongside behavior over 10 days of learning, we identify key activity features that mechanistically link tau pathology to impaired spatial memory formation.

#### Impaired path integration potentially leads to unsuccessful spatial memory in PS19 mice

Among aberrant activity features reported previously^39–42^ and in our study, a prominent predictor of learning performance was the ability of the MEC map to gradually stabilize into a coherent global representation. PS19 males showed reduced stabilization of spatial representations, consistent with our prior finding that MEC spatial consistency is necessary for successful learning^49^. This instability parallels observations in 3xTg mice, which model both amyloid-β and tau pathology^126^, in which spatial consistency of hippocampal place cells likewise fails to increase across learning^127^.

In addition to instability, MEC spatial representations of PS19 mice were biased toward cue-rich regions, with weaker encoding of non-cue areas. This bias was accompanied by reduced speed modulation and a lower proportion of speed-modulated MEC neurons, features that are critical for path integration based on self-motion signals independent of external landmarks^6,99^. Consistent with this interpretation, PS19 mice showed a strong association between impaired speed encoding and excessive cue anchoring, suggesting that disrupted path integration prevents the formation of a coherent, global spatial map. Supporting this view, impaired path integration has previously been reported in PS19 mice and was associated with pTau accumulation in the EC^61^. The degree of cue anchoring and impairment in speed modulation was more pronounced in pyramidal cells, which exhibited the strongest pTau accumulation, suggesting a cell-autonomous effect of pTau on these activity deficits. Additionally, such deficits may partly arise from dysfunction of the locus coeruleus (LC)^61,128–130^, which also exhibits robust tau burden in PS19 mice^128,129^ and sends noradrenergic projections to multiple brain regions, including the MEC, to control motor function, attention, and memory^131,132^. Consistent with our observations, reduced speed modulation has also been reported in rTg4510 tauopathy mice^40^.

Although the cellular mechanisms cannot be directly assessed in humans, impaired navigation based on self-motion cues and deficits in path integration have been consistently observed in older adults^133,134^ and in individuals in early AD^135–137^. Notably, the LC is the first site showing abnormally phosphorylated pretangle tau, preceding the onset of prion-like seeding activity of abnormal tau in the EC^3,26,138,139^, and LC neurons show progressive degeneration that correlates with cognitive decline^140^. These findings are consistent with a contributory role of LC dysfunction in the observed path integration deficits.

Together, these convergent findings support a model in which impaired self-motion computation in AD promotes overreliance on external landmarks, disrupts path integration, destabilizes spatial representations, and ultimately impairs spatial memory formation.

#### Inflexibility of MEC maps in PS19 mice

We observed reduced experience-dependent flexibility of MEC map dynamics in PS19 mice, as both male and female mice showed minimal changes in map consistency within days, whereas WT mice displayed the expected initial disruption followed by progressive stabilization. WT mice also exhibited increased speed modulation as learning proceed, but PS19 males failed to show the change and remained at a low level, reflecting an inflexibility of neural activity with respect to speed over the course of learning. Similar hippocampal inflexibility has been reported in APP mice, where neuronal firing patterns remained unchanged throughout learning, whereas WT cell activity became increasingly task-specific^141^. This neural rigidity likely impairs adaptation to novel environments and may reflect tau-pathology-related deficits in synaptic plasticity^142^.

#### Widespread entorhinal circuit disruption in PS19 mice

While previous studies focused primarily on grid cell dysfunction^39–42^ and reported inconsistent impairment of non-grid cells^39,40^, we found that activity deficits in PS19 grid cells extended to non-grid cells. Both populations contributed to predicting learning behavior, with non-grid cells performing even better than grid cells, and predictions based on the full ensemble surpassing either population alone. These findings support the view that accurate spatial representation arises from integrated activity across heterogeneous MEC neurons, rather than from a specialized functional subgroup^143^. Importantly, our results indicate that spatial memory impairments in tauopathy reflect widespread entorhinal circuit disruption.

#### Contributions of other brain regions

While MEC activity features explained over half of behavioral variance, residual variance likely stems from dysfunction in other regions, such as the LC discussed above. Another probable contributor is the hippocampus, which is also heavily involved in spatial cognition^6^ and accumulates phosphorylated tau and neurofibrillary tangles in PS19 mice^58^. Indeed, the hippocampal circuit in PS19 mice shows synaptic pathology, neural degeneration, altered glia dysfunction, and transcriptomic changes^58,144,145^. Moreover, tau pathology may propagate from MEC to hippocampal subfields, including the dentate gyrus and CA fields, further disrupting hippocampal circuits during spatial learning^112,113^.

### Difference in molecular cell types

#### Tau pathology is associated with impaired spatial encoding at the cellular level

We examined molecularly defined excitatory cell types in MEC layer 2—stellate and pyramidal cells—in PS19 mice. Tau pathology preferentially accumulated in pyramidal cells, which exhibited more severe activity impairments than stellate cells, including reduced spatial selectivity and speed modulation, lower activity stability, and stronger cue anchoring during learning. Given that pyramidal cells are highly speed-modulated and contribute to self-motion processing^146,147^, their selective vulnerability provides a mechanistic link between tau accumulation and impaired path integration, which may destabilize spatial representations and ultimately lead to spatial memory deficits. Accordingly, pyramidal cells in male PS19 mice exhibited higher AT8 immunoreactivity than females, paralleling their more severe learning deficits, and pyramidal cell activity better predicted learning performance across genotypes. Although *in vivo* labeling of intracellular tau in 7–10-month-old mice remains technically challenging, owing to the limited availability of well-characterized tau-specific fluorescent reporters and high brain autofluorescence in aging animals^148^, our results nonetheless provide compelling evidence for the association between tau accumulation and impaired spatial encoding at the cellular level.

Consistent with our observations, in humans, calbindin+ pyramidal cell clusters are most prominent in the caudal EC layer 2^149^, coinciding with robust pTau accumulation in the same region in very early Braak stages of AD^3,150^. At this stage, pTau and pyramidal cells also exhibit strong colocalization at the cellular level^83^. Tau in pyramidal cells of EC layer 2 can be further propagated to CA1, disrupting neural excitability and contextual learning^83^. While these findings together suggest that pyramidal cell dysfunction in EC layer 2 may underlie early EC–hippocampal circuit deficits and spatial memory impairments in early AD, other studies reported that layer 2 reelin+ stellate cells are preferentially susceptible to tau pathology^151^. Further research is needed to clarify the relative contributions of pyramidal and stellate cell dysfunction to early tau-related circuit impairments.

#### Tau pathology affects neural excitability in a cell-type-, sex-, and learning-dependent manner

Interestingly, although PS19 mice showed an increased fraction of active neurons—driven primarily by stellate cells—the activity of individual stellate and pyramidal neurons varied in a sex- and learning-dependent manner, indicating a complex and dynamic impact of tau pathology on MEC excitability. These findings extend prior reports of MEC hypoactivity^40^, including deficits in grid cells^39^ and stellate cells^41^ by revealing previously unrecognized modulation by sex and learning stage. Notably, intracellular pTau accumulation—more prominent in pyramidal cells— did not directly correlate with overall activity levels, suggesting that neuronal excitability is not solely driven by pTau or neurofibrillary tangles. Instead, altered excitability may be mediated by soluble tau species, consistent with *in vivo* two-photon imaging studies showing tau-induced hypoactivity independent of tangle formation^152,153^.

In contrast, stellate cell hyperexcitability has also been documented in APP-transgenic rats^51^, consistent with the well-established role of amyloid-β in promoting neural hyperactivity across brain regions^154^. Amyloid-β-induced neuronal hyperexcitability has been linked to plaques^155,156^, intraneuronal amyloid-β^157^, and soluble amyloid-β species^155,158^. Notably, in 3xTg-AD mice, stellate cells were hyperexcitable across ages, whereas pyramidal cell hyperactivity was restricted to younger mice^42^. These findings raise the possibility that the combined effects of amyloid-β and tau may differentially affect MEC network activity over time and across cell types compared to each of them alone.

Overall, our results suggest that both pyramidal and stellate cells contribute to tauopathy-related dysfunction, but likely via distinct mechanisms tied to their differential susceptibility to tau pathology and their unique roles in MEC circuit dynamics.

### Sex-specific behavioral and neural activity impairments

We observed pronounced sex-specific differences in MEC tau pathology and associated circuit dysfunction. Although males showed a trend toward a smaller overall pTau-positive area and similar proportions of pTau-positive stellate and pyramidal neurons compared with females, the local intensity of pTau within positive regions—particularly in pyramidal neurons—was higher in males, indicating a greater local tau burden. Correspondingly, male PS19 mice exhibited more severe spatial learning impairments and disrupted neural activity than females. Our observations are consistent with previous studies reporting impaired spatial memory in PS19 males, but not females^76,77^, although one study observed impairments primarily in females at more advanced ages^75^.

The generally more severe spatial learning and neural activity deficits in males could arise from at least two non–mutually exclusive mechanisms. First, local tau burden within the MEC—particularly in pyramidal neurons—rather than the overall extent of tau spread, may be a primary determinant of spatial learning impairment. This interpretation aligns with human imaging and neuropathological studies showing that local tau burden in the entorhinal-hippocampal circuit correlates with cognitive decline more strongly than measures of tau spread alone^159,160^.

Second, because sex differences in tau distribution across the MEC were modest, additional sex-dependent molecular and cellular responses to tau pathology likely contribute to the observed behavioral and neural activity differences. Previous studies showed that although PS19 males and females exhibit similar levels of pathogenic tau isoforms across the brain, males display higher tau phosphorylation, increased astrocyte activation, elevated inflammatory signaling, and more pronounced microglial transcriptomic alterations^74,75^. Additionally, hormonal factors may further modulate vulnerability. Estrogen exerts neuroprotective effects in AD models^161^ and in mouse models of other conditions, such as stress^162^ and chronic liver disease^163^, consistent with the increased risk of post-menopausal women developing AD^164,165^ and with the relative preservation of spatial learning in female PS19 mice. Human transcriptomic studies also indicate that females exhibit distinct gene expression programs associated with tau pathology^166^, which may confer greater resilience to tau-related dysfunction.

Together, our findings highlight sex as a critical biological variable shaping tau-associated circuit dysfunction and spatial memory impairment, with implications for understanding sex-specific vulnerability in AD models^167,168^ and, ultimately, in human AD^169^.

### Clinical implications of our study

Our work supports a model in which tau pathology disrupts EC circuit function during aging and early in AD, leading to impairments in spatial cognition. By establishing a mechanistic link between tau burden, EC neural dynamics, and learning behavior, our study positions EC activity as both a clinically relevant biomarker for the diagnosis of early AD and a potential therapeutic target for preserving cognitive function in aging and AD.

First, our study identifies EC neural activity as a promising functional biomarker for aging- and early AD-related tau pathology. We show that EC activity is disrupted at a network level early in disease progression, closely correlates with local pTau burden, robustly predicts spatial learning performance, and reliably distinguishes tauopathy mice from controls—all prior to overt neurodegeneration. These results are highly relevant to human aging and disease, as converging clinical studies have reported impaired grid-cell-like representations in individuals at risk for AD^9,11^, as well as abnormal functional MRI activity of medial temporal lobe (MTL), including EC, associated with elevated tau burden and age-related memory decline^170^. Moreover, the EC activity abnormalities identified here align with deficits in path integration, which is a behavioral marker correlated with tau pathology in healthy adults^27^ and sensitively predicts AD risk before the onset of clinically detectable cognitive impairment^136,171,172^. Together, these findings indicate that EC functional measures may provide an early readout of tau pathology progression that complements established molecular and structural biomarkers, such as tau PET imaging and MTL atrophy^173,174^.

Second, our results suggest that EC circuit dysfunction itself represents a clinically actionable target for the treatment of AD-related tauopathy. Beyond molecular pathology, we identify specific neural activity features—including impaired speed modulation, unstable spatial representations, strong cue anchoring, reduced spatial selectivity, and altered activity levels—that are tightly linked to memory impairment. These findings raise the possibility that therapeutic strategies aimed at restoring EC network function—such as neuromodulation^174^, targeted stimulation^175,176^, or structured behavioral interventions^177,178^—could mitigate cognitive decline even when tau pathology is already present. Such circuit-level approaches could therefore enhance or extend the efficacy of disease-modifying therapies by stabilizing neural computations underlying spatial memory. Thus, our study will motivate future strategies to preserve or enhance EC function.

## Supplementary Material

Supplement 1

1

## Data Availability

All data used for figures and analysis in the study are available upon request from the lead contact (Dr. Yi Gu, yi.gu@nih.gov).

## CODE AVAILABILITY:

Custom MATLAB scripts used for data analysis and figures are available on GitHub (https://github.com/tjmalone/Malone2026).
