# Supplementary material for: Aberrant medial entorhinal cortex dynamics link tau pathology to spatial memory impairment
Source: bioRxiv. 2026 Feb 6:2025.12.30.696887. Originally published 2025 Dec 31. Preprint. [Version 2] doi: 10.64898/2025.12.30.696887 (PMC12776407; doi:10.64898/2025.12.30.696887)
Supplement: 1 [file NIHPP2025.12.30.696887V2-supplement-1.pdf]

bioRxiv preprint doi: https://doi.org/10.64898/2025.12.30.696887; this version posted February 6, 2026. The copyright holder for this preprint (which was not certified by peer review) is the author/funder. This article is a US Government work. It is not subject to copyright under 17 USC 105 and is also made available for use under a CC0 license.



Supplementary Information for

# Aberrant medial entorhinal cortex dynamics link tau pathology to spatial memory impairment

Taylor J. Malone[1], Kyle Cekada[1,2], Jean Tyan[1,3], Lujia Chen[1], Garret Wang[1,4], Yi Gu[1,5*]

Correspondence to: yi.gu@nih.gov

bioRxiv preprint doi: https://doi.org/10.64898/2025.12.30.696887; this version posted February 6, 2026. The copyright holder for this preprint (which was not certified by peer review) is the author/funder. This article is a US Government work. It is not subject to copyright under 17 USC 105 and is also made available for use under a CC0 license.



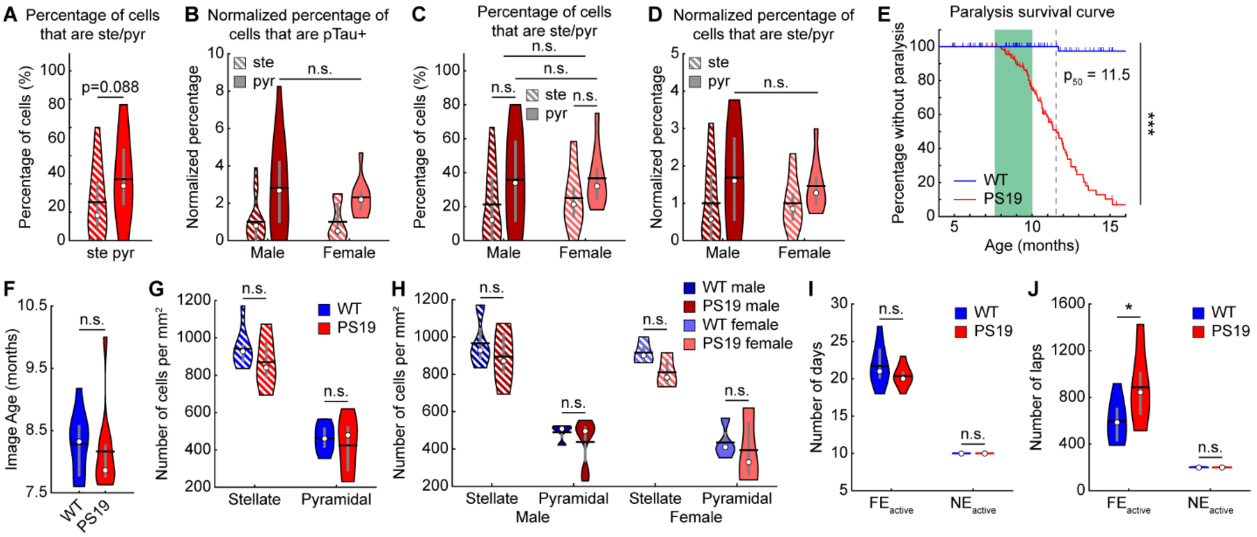


**Figure S1: Pyramidal cells in PS19 mice show increased tau pathology.** Related to **Figure 1**

**A.** Percentage of pTau+ cells overlapping with stellate (ste) and pyramidal (pyr) cells in all PS19 mice.

**B.** Percentage of stellate and pyramidal cells overlapping with pTau+ cells in PS19 mice split by sex and normalized by stellate level for each sex.

**C, D.** Percentage of pTau+ cells overlapping with stellate and pyramidal cells in PS19 mice split by sex. **C.** Raw overlap percentage. **D.** Normalized by stellate level for each sex.

**A-D.** Paired/Unpaired Student's t-test.

**E.** Survival curve representing cumulative onset of hindlimb paralysis in WT and PS19 mice. Dashed line represents median PS19 value ($p_{50}$). Green box represents age range of imaged mice. Ticks represent censored data points. Log-rank test.

**F.** Mouse age at the start of imaging.

**G, H.** Number of stellate and pyramidal cells per area in all mice (**G**) or for each sex (**H**).

**I, J.** Number of training or imaging days (**I**) or laps (**J**) experienced in the familiar enviorment during active learning (training and imaging) or in the novel enviornment during imaging.

**F-J.** Unparied Student's t-test.

*$p \leq 0.05$, **$p \leq 0.01$, ***$p \leq 0.001$. In violin plots, horizontal line represents mean, white circle represents median, and whiskers represent interquartile range. Statistical details can be found in **Table S1**.

bioRxiv preprint doi: https://doi.org/10.64898/2025.12.30.696887; this version posted February 6, 2026. The copyright holder for this preprint (which was not certified by peer review) is the author/funder. This article is a US Government work. It is not subject to copyright under 17 USC 105 and is also made available for use under a CC0 license.



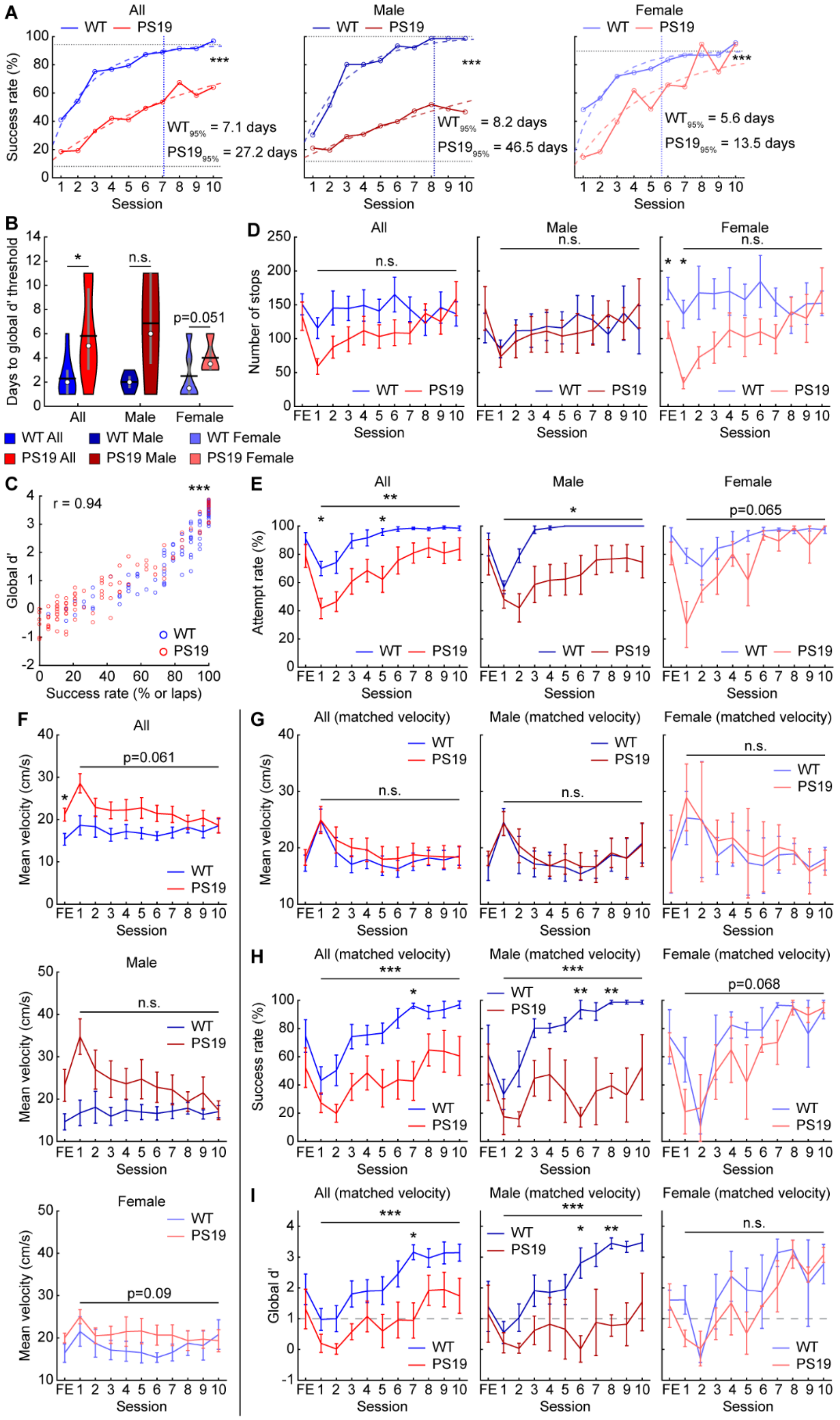


**Figure S2: Male PS19 mice show behavioral deficits in an active spatial learning task.** Related to **Figure 1**
**A.** Percentage of laps that mice successfully stopped to receive water reward across learning in all mice (left), male mice (middle), and female mice (right). $WT_{95\%}$ and $PS19_{95\%}$: number of days to reach 95% of plateau value.

bioRxiv preprint doi: https://doi.org/10.64898/2025.12.30.696887; this version posted February 6, 2026. The copyright holder for this preprint (which was not certified by peer review) is the author/funder. This article is a US Government work. It is not subject to copyright under 17 USC 105 and is also made available for use under a CC0 license.



Solid lines and circles: mean success rate of each group from **Fig. 1N**. Dashed colored curves: fit of one-phase association exponential. Horizontal dotted lines: mutual baseline and plateau values used for exponential fit. Vertical dotted line: number of days for WT mice to reach 95% of plateau value. Extra sum-of-squares F test. All mice: $WT_K = 0.42$, $PS19_K = 0.11$. Male mice: $WT_K = 0.37$, $PS19_K = 0.064$. Female mice: $WT_K = 0.53$, $PS19_K = 0.22$.

**B.** Number of days for global reward zone discrimination (global d') to reach the learning threshold of 1. Unpaired t-test.

**C.** Correlation between success rate and global d' for all mice and sessions. Pearson correlation was calculated for both genotypes combined.

**D.** Number of stops per session across learning in all mice (left), male mice (middle), and female mice (right).

**E.** Percentage of laps that mice attempted to receive water reward across learning in all mice (left), male mice (middle), and female mice (right). An attempt was defined as 0.5 seconds (decreased from 1s) with speed below 5 cm/s (increased from 1 cm/s) within a 100 cm zone centered on the true 50 cm reward zone.

**F.** Mean session velocity across learning in all mice (top), male mice (middle), and female mice (bottom).

**G-I.** Mean session velocity (**G**), success rate (**H**), and global d' (**I**) across learning in velocity matched mice for all mice (left), male mice (middle), and female mice (right). Velocity was matched by selecting pairs of mice on each day with similar velocity (within 5 cm/s).

**D-I.** Horizontal gray bars indicate p values for the group difference (**D-F:** two-way repeated measures ANOVA, **G-I:** ordinary two-way ANOVA). Individual time points are compared using Student's t-test with Bonferroni-Holm correction.

*$p \leq 0.05$, **$p \leq 0.01$, ***$p \leq 0.001$. In violin plots, horizontal line represents mean, white circle represents median, and whiskers represent interquartile range. Error bars in line plots represent mean ± sem. Statistical details can be found in **Table S1**.

bioRxiv preprint doi: https://doi.org/10.64898/2025.12.30.696887; this version posted February 6, 2026. The copyright holder for this preprint (which was not certified by peer review) is the author/funder. This article is a US Government work. It is not subject to copyright under 17 USC 105 and is also made available for use under a CC0 license.



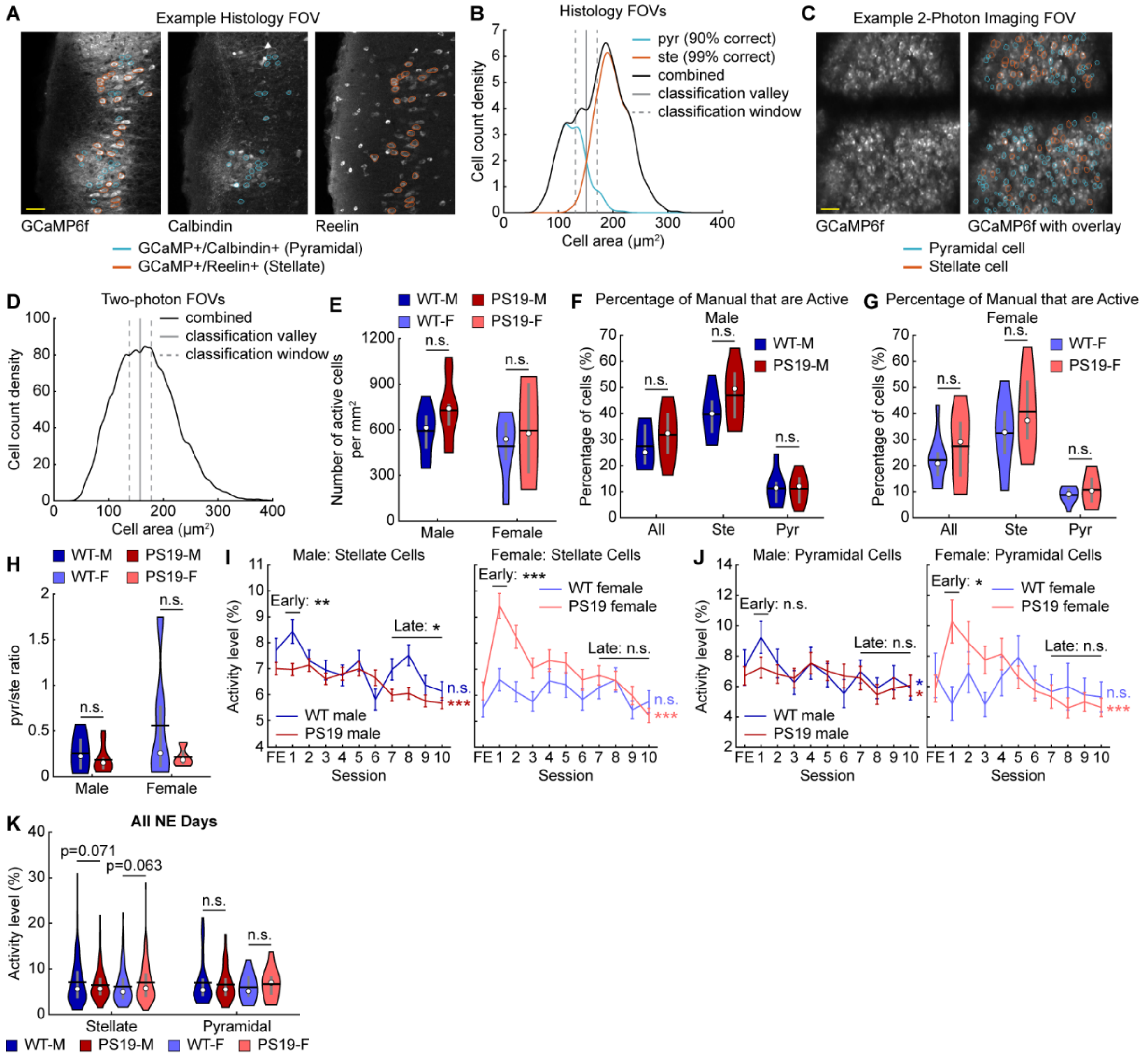


**Figure S3: MEC cells of PS19 mice show hyper- or hypoactivity depending on molecular identity and sex.** Related to **Figure 2**.

**A.** Example histology FOV showing the overlap of reelin (stellate cell marker) and calbindin (pyramidal cell marker) with GCaMP6f. Cells were manually outlined if they had both GCaMP6f and reelin or calbindin staining. Used to validate differentiation of stellate and pyramidal cells based on cell area. Scale bar: 50µm

**B.** Histology validation using area to differentiate stellate (ste) and pyramidal (pyr) cells. Colored lines indicate the true size distribution of stellate and pyramidal cells. The true positve rate of stellate and pyramidal cell idenification segementation (gray lines) using the combined area distribution (black line) is indicated.

**C.** Example calcium imaging FOV with no overlay (left) and with an overlay of manually outlined cells identified as pyramidal or stellate cells (right). Scale bar: 50µm

**D.** The size distribution of manually outlined cells from the FE day of calcium imaging with the segmentation lines marked as in **B** (158 +/- 20µm).

**E.** The number of active cells per area on the FE day split by sex. WT-M: WT Male, PS19-M: PS19 Male, WT-F: WT Female, PS19-F: PS19 Female

**F, G.** The percentage of all manually outlined cells and manually outlined stellate and pyramidal cells in male (**F**) or female (**G**) mice that are active on the FE day.

bioRxiv preprint doi: https://doi.org/10.64898/2025.12.30.696887; this version posted February 6, 2026. The copyright holder for this preprint (which was not certified by peer review) is the author/funder. This article is a US Government work. It is not subject to copyright under 17 USC 105 and is also made available for use under a CC0 license.



**H.** The ratio between the fraction of active cells that are identified as pyramidal cells and as stellate cells split by sex. A low value indicates stellate cell enrichment.

**I, J.** Activity level across learning in male (left) and female (right) mice in stellate (**I**) and pyramidal cells (**J**).

**K.** Activity level averaged across all NE days split by sex and morphological cell type.

*$p \leq 0.05$, **$p \leq 0.01$, ***$p \leq 0.001$. In violin plots, horizontal line represents mean, white circle represents median, and whiskers represent interquartile range. Violin plots use unpaired Student's t-test. Error bars in line plots represent mean ± sem. In line plots, horizontal gray bars indicate p values for the group difference (early learning: unpaired Student's t-test, late learning: two-way repeated measures ANOVA), and p-values to the right indicate significant Pearson correlation of the mean value with respect to time. Statistical details can be found in **Table S1**.

bioRxiv preprint doi: https://doi.org/10.64898/2025.12.30.696887; this version posted February 6, 2026. The copyright holder for this preprint (which was not certified by peer review) is the author/funder. This article is a US Government work. It is not subject to copyright under 17 USC 105 and is also made available for use under a CC0 license.



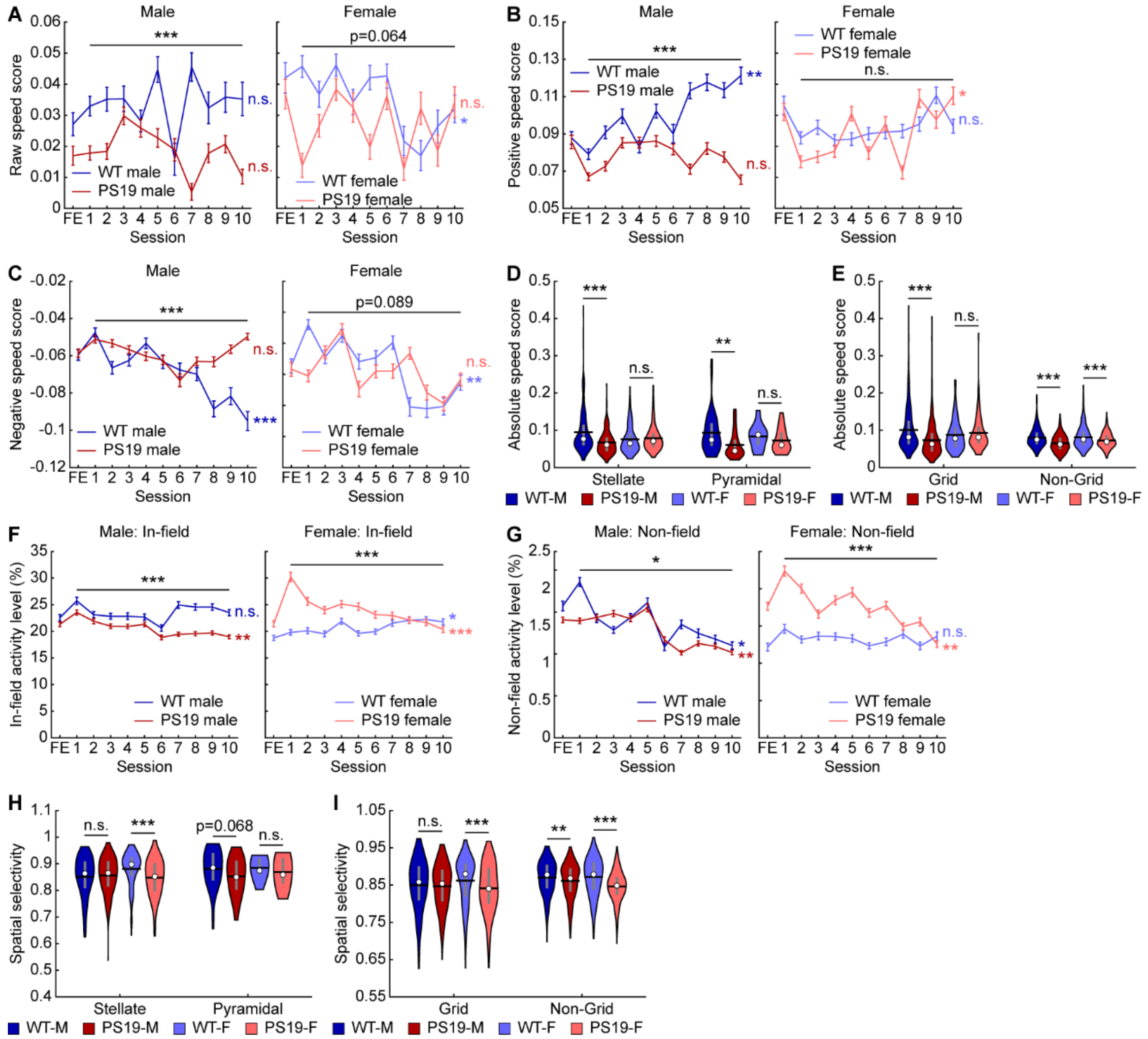


**Figure S4: PS19 mice have altered speed modulation and spatial selectivity.** Related to **Figure 2**.
**A.** Raw speed score across learning in male (left) and female (right) mice.
**B, C.** Positive (**B**) and negative (**C**) speed scores across learning in male (left) and female (right) mice.
**D, E.** Absolute speed score averaged across all NE days split by sex and morphological (**D**) and functional (**E**) cell type.
**F, G.** Activity level across learning in male (left) and female (right) mice within track regions with fields (In-field, **F**) and in other track locations (Non-field, **G**).
**H, I.** Spatial selectivity averaged across all NE days split by sex and morphological (**H**) and functional (**I**) cell type.
$*p \leq 0.05$, $**p \leq 0.01$, $***p \leq 0.001$. In violin plots, horizontal line represents mean, white circle represents median, and whiskers represent interquartile range. Violin plots use unpaired Student's t-test. Error bars in line plots represent mean ± sem. In line plots, horizontal gray bars indicate p values for the group difference (early learning: unpaired Student's t-test, all learning days: linear mixed-effects model), and p-values to the right indicate significant Pearson correlation of the mean value with respect to time. Statistical details can be found in **Table S1**.

bioRxiv preprint doi: https://doi.org/10.64898/2025.12.30.696887; this version posted February 6, 2026. The copyright holder for this preprint (which was not certified by peer review) is the author/funder. This article is a US Government work. It is not subject to copyright under 17 USC 105 and is also made available for use under a CC0 license.



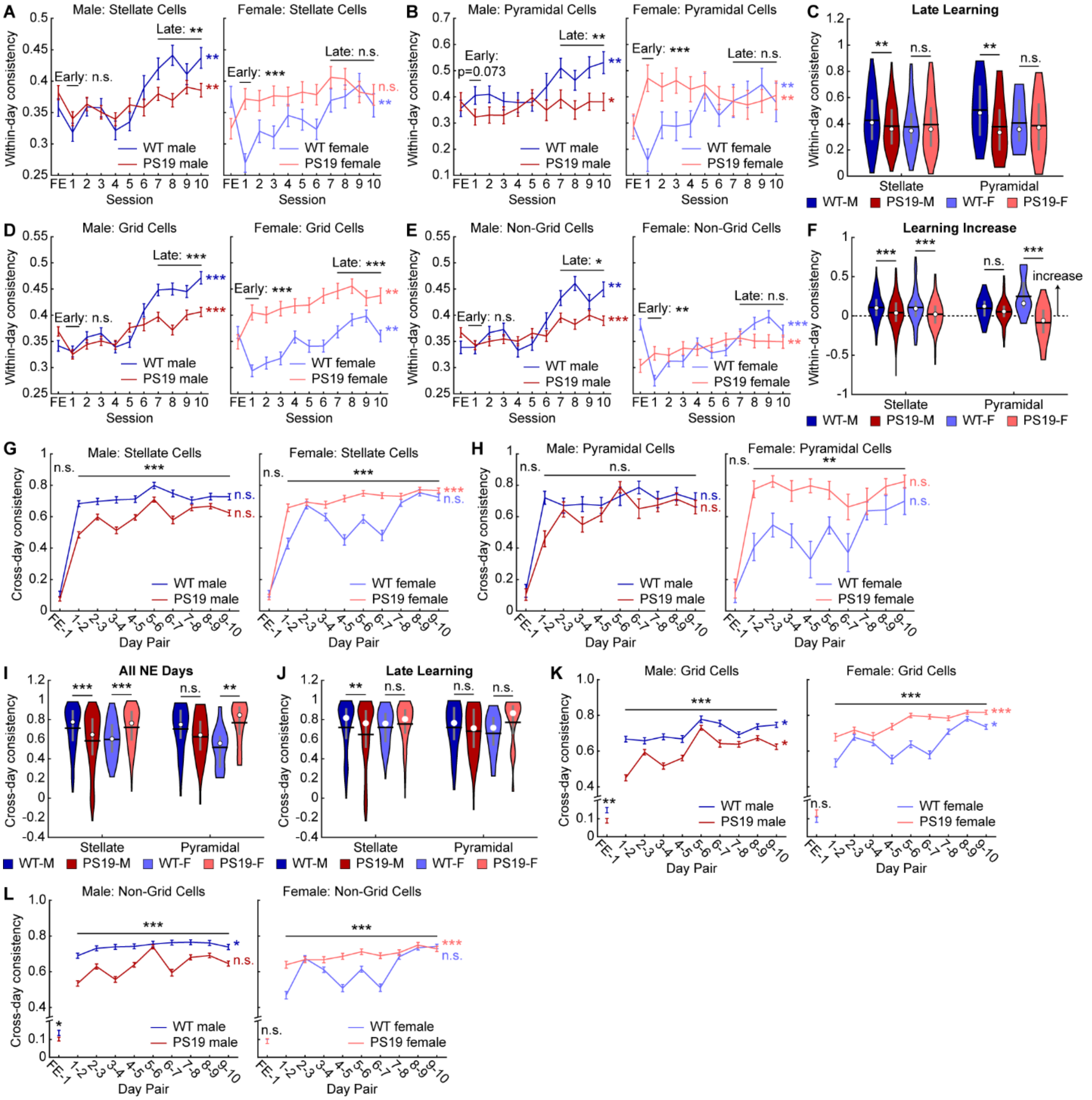


**Figure S5: Male PS19 mice have impaired MEC within-day and cross-day activity consistency.** Related to **Figure 3**.

**A, B.** Within-day activity consistency across learning in male (left) and female (right) mice in stellate (**A**) and pyramidal cells (**B**).

**C.** Within-day activity consistency averaged across late learning (days 7-10) split by sex and morphological cell type. WT-M: WT Male, PS19-M: PS19 Male, WT-F: WT Female, PS19-F: PS19 Female

**D, E.** Within-day activity consistency across learning in male (left) and female (right) mice in grid (**D**) and non-grid cells (**E**).

**F.** The increase in within-day activity consistency between early learning (day 1) and late learning split by sex and morphological cell type.

**G, H.** Cross-day activity consistency across learning in male (left) and female (right) mice in stellate (**G**) and pyramidal cells (**H**).

bioRxiv preprint doi: https://doi.org/10.64898/2025.12.30.696887; this version posted February 6, 2026. The copyright holder for this preprint (which was not certified by peer review) is the author/funder. This article is a US Government work. It is not subject to copyright under 17 USC 105 and is also made available for use under a CC0 license.



**I, J.** Cross-day activity consistency averaged across all learning days (**I**) and late learning (**J**) split by sex and morphological cell type.

**K, L.** Cross-day activity consistency across learning in male (left) and female (right) mice in grid (**K**) and non-grid cells (**L**).

**p* ≤ 0.05, ***p* ≤ 0.01, ****p* ≤ 0.001. In violin plots, horizontal line represents mean, white circle represents median, and whiskers represent interquartile range. Violin plots use unpaired Student's t-test. Error bars in line plots represent mean ± sem. In line plots, horizontal gray bars indicate p values for the group difference (early learning/ FE-1: unpaired Student's t-test, late learning/all learning days: two-way repeated measures ANOVA or linear mixed-effects model), and p-values to the right indicate significant Pearson correlation of the mean value with respect to time. Statistical details can be found in **Table S1**.

bioRxiv preprint doi: https://doi.org/10.64898/2025.12.30.696887; this version posted February 6, 2026. The copyright holder for this preprint (which was not certified by peer review) is the author/funder. This article is a US Government work. It is not subject to copyright under 17 USC 105 and is also made available for use under a CC0 license.



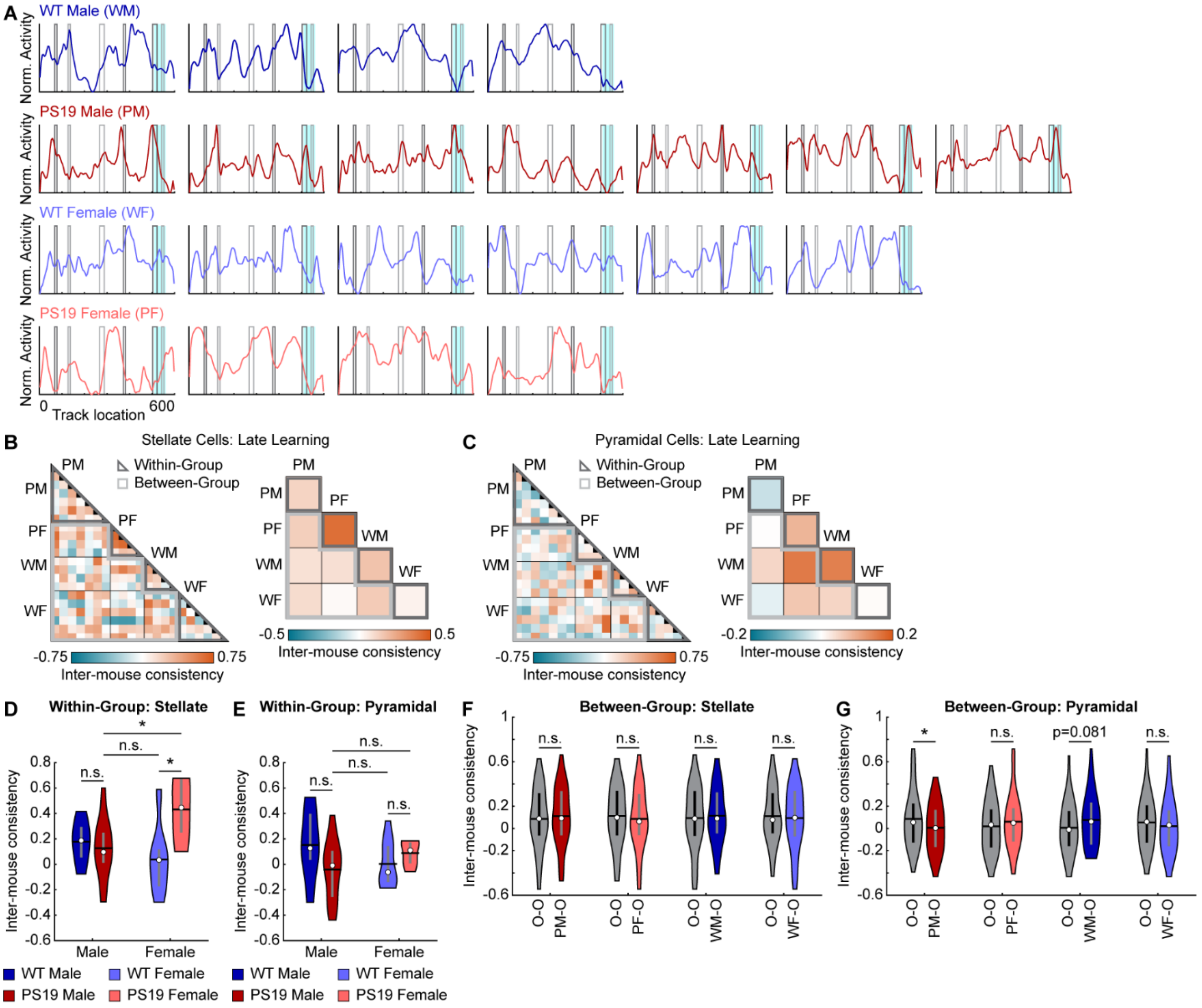


**Figure S6: Male PS19 mice do not develop a cognitive map shared by other mouse groups.** Related to **Figure 4**.
**A.** Late-learning spatial activity maps as calculated in **Fig. 4A** for individual mice. From top to bottom: WT male, PS19 male, WT female, PS19 female. Black and grey rectangles represent left and right cues, respectively. Blue rectangle represents reward zone.
**B, C.** Inter-mouse consistency for stellate (**B**) and pyramidal cells (**C**). Left: Pairwise inter-mouse consistency sorted by genotype/sex group. Right: Average inter-mouse consistency for each group-to-group block.
**D, E.** Pooled inter-mouse consistency for pairwise within-group comparisons for stellate (**D**) and pyramidal (**E**) cells. Unpaired Student's t-test with Bonferroni-Holm correction.
**F, G.** Pooled inter-mouse consistency for pairwise between-group comparisons for stellate (**F**) and pyramidal (**G**) cells with each genotype/sex group serving as the reference group compared to the other genotype/sex groups (O) as indicated. Unpaired Student's t-test.
*$p \leq 0.05$, **$p \leq 0.01$, ***$p \leq 0.001$. In violin plots, horizontal line represents mean, white circle represents median, and whiskers represent interquartile range. Statistical details can be found in **Table S1**.

bioRxiv preprint doi: https://doi.org/10.64898/2025.12.30.696887; this version posted February 6, 2026. The copyright holder for this preprint (which was not certified by peer review) is the author/funder. This article is a US Government work. It is not subject to copyright under 17 USC 105 and is also made available for use under a CC0 license.



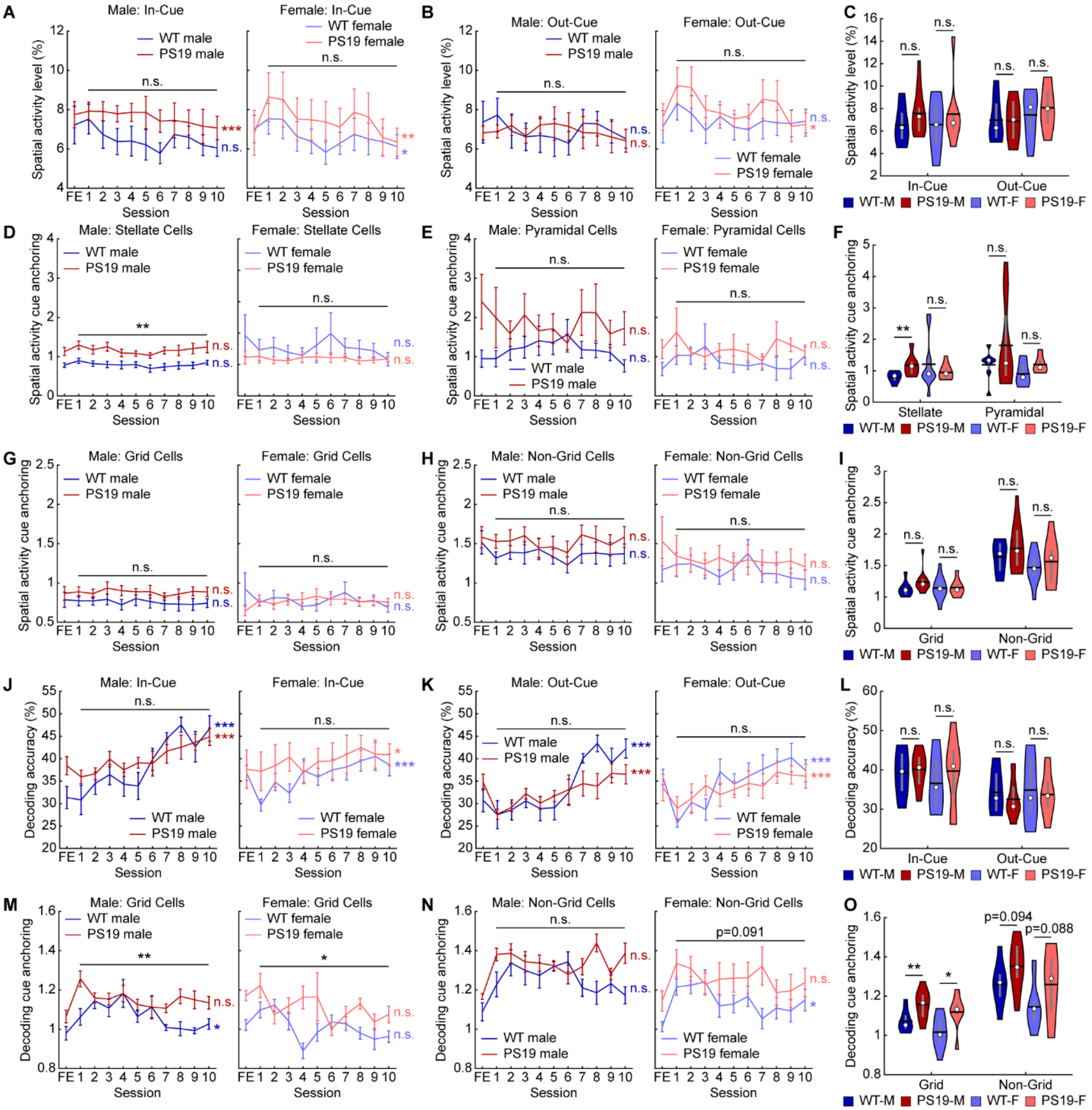


**Figure S7: Spatial activity of male PS19 mice is anchored to visual cues.** Related to **Figure 5**.

**A, B.** Spatial activity level within in-cue (**A**) and out-cue (**B**) regions of the track across learning in male (left) and female (right) mice.

**C.** Spatial activity level within in-cue and out-cue regions of the track averaged across all learning days. WT-M: WT Male, PS19-M: PS19 Male, WT-F: WT Female, PS19-F: PS19 Female

**D, E, G, H.** Spatial activity cue anchoring across learning in male (left) and female (right) mice in stellate (**D**), pyramidal (**E**), grid (**G**), and non-grid (**H**) cells.

**F, I.** Spatial activity cue anchoring averaged across all learning days split by morphological (**F**) and functional (**I**) cell type.

**J, K.** Decoding accuracy within in-cue (**J**) and out-cue (**K**) regions of the track across learning in male (left) and female (right) mice.

bioRxiv preprint doi: https://doi.org/10.64898/2025.12.30.696887; this version posted February 6, 2026. The copyright holder for this preprint (which was not certified by peer review) is the author/funder. This article is a US Government work. It is not subject to copyright under 17 USC 105 and is also made available for use under a CC0 license.



**L.** Decoding accuracy within in-cue and out-cue regions of the track averaged across all learning days.

**M, N.** Decoding cue anchoring across learning in male (left) and female (right) mice in grid (**M**) and non-grid (**N**) cells.

**O.** Decoding cue anchoring averaged across all learning days split by functional cell type.

*$p \leq 0.05$, **$p \leq 0.01$, ***$p \leq 0.001$. In violin plots, horizontal line represents mean, white circle represents median, and whiskers represent interquartile range. Violin plots use unpaired Student's t-test. Error bars in line plots represent mean ± sem. In line plots, horizontal gray bars indicate p values for the group difference (linear mixed-effects model), and p-values to the right indicate significant Pearson correlation of the mean value with respect to time. Statistical details can be found in **Table S1**.

bioRxiv preprint doi: https://doi.org/10.64898/2025.12.30.696887; this version posted February 6, 2026. The copyright holder for this preprint (which was not certified by peer review) is the author/funder. This article is a US Government work. It is not subject to copyright under 17 USC 105 and is also made available for use under a CC0 license.



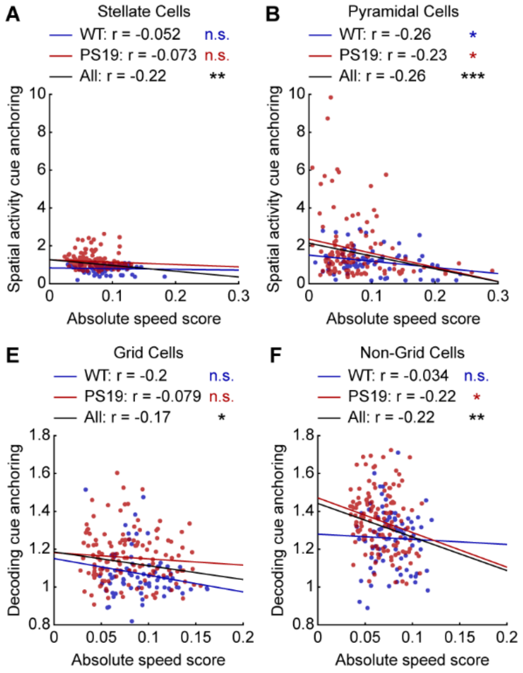


**Figure S8: Speed modulation is correlated with cue anchoring in male PS19 mice.** Related to **Figure 5**.
**A-F.** Correlation between absolute speed score and spatial activity cue anchoring (**A-D**) or decoding cue anchoring (**E, F**) in stellate (**A**), pyramidal (**B**), grid (**C, E**), and non-grid (**D, F**) cells in male mice. Each point represents the activity from one FOV from one session. Pearson correlation.
*$p \leq 0.05$, **$p \leq 0.01$, ***$p \leq 0.001$. Statistical details can be found in **Table S1**.

bioRxiv preprint doi: https://doi.org/10.64898/2025.12.30.696887; this version posted February 6, 2026. The copyright holder for this preprint (which was not certified by peer review) is the author/funder. This article is a US Government work. It is not subject to copyright under 17 USC 105 and is also made available for use under a CC0 license.



| Activity Category | Activity Features | Learning Period | Male PS19 Compared to Male WT | Female PS19 Compared to Female WT | Figure |
|---|---|---|---|---|---|
| General Activity Deficits | Activity Level | Early Learning | Low | High | 2D |
| | | Late Learning | Low | Low | 2D |
| | Absolute Speed Score | All NE Days | Low | No difference | 2K |
| | Spatial Selectivity | All NE Days | No difference | Low | 2O |
| Map Consistency | Within-Day Consistency | Late Learning | Low | No difference | 3C |
| | | Learning Increase | Low | Low | 3E |
| | Cross-Day Consistency | All NE Days | Low | High | 3I |
| | | Late Learning | Low | High | 3J |
| | Inter-Mouse Consistency | All NE Days | Low (compared to others) | High (compared to others) | 4H, I |
| Global Representation | Spatial Activity Cue Anchoring | All NE Days | High | No difference | 5C |
| | Decoding Cue Anchoring | All NE Days | High | No difference | 5J |

Under-Effect

Over-Effect

**Figure S9: Summary table of activity under-effects and over-effects in PS19 mice relative to sex-matched WT mice.**

bioRxiv preprint doi: https://doi.org/10.64898/2025.12.30.696887; this version posted February 6, 2026. The copyright holder for this preprint (which was not certified by peer review) is the author/funder. This article is a US Government work. It is not subject to copyright under 17 USC 105 and is also made available for use under a CC0 license.



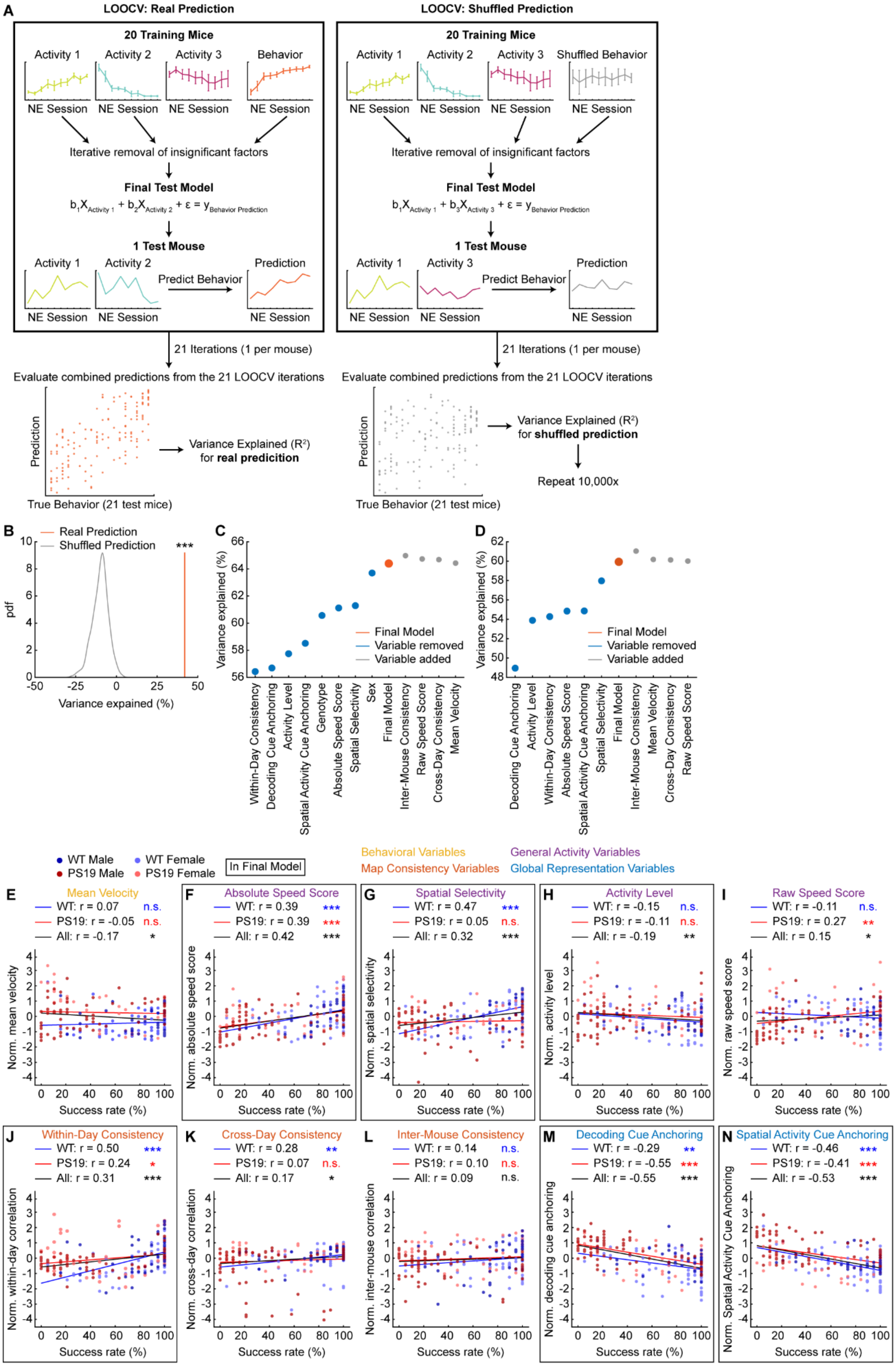

bioRxiv preprint doi: https://doi.org/10.64898/2025.12.30.696887; this version posted February 6, 2026. The copyright holder for this preprint (which was not certified by peer review) is the author/funder. This article is a US Government work. It is not subject to copyright under 17 USC 105 and is also made available for use under a CC0 license.



**Figure S10: GLM model selection to predict mouse behavior using MEC activity.** Related to **Figure 7**.
**A.** Schematic of final model selection method.
**B.** Evaluation of model selection method by comparing model predictions based on true behavior with shuffled behavior models.
**C.** Effect of removing variables from final model or adding excluded variables back to final model on percentage of behavioral variance explained ($R^2$).
**D.** Similar to **C** but excluding sex and genotype from all models.
**E-N.** Correlation between normalized predictor variables and success rate. Each point represents one session from one mouse. Dot colors indicate sex and genotype. Final model variables are outlined in black. Pearson correlations were calculated for all mice, all WT mice, and all PS19 mice. **E**: Mean velocity. **F**: Absolute speed score **G**: Spatial selectivity. **H**: Activity level. **I**: Raw speed score. **J**: Within-day consistency. **K**: Cross-day consistency. **L**: Inter-mouse consistency. **M**: Decoding cue anchoring. **N**: Spatial activity cue anchoring.
*$p \leq 0.05$, **$p \leq 0.01$, ***$p \leq 0.001$. Statistical details can be found in **Table S1**.

bioRxiv preprint doi: https://doi.org/10.64898/2025.12.30.696887; this version posted February 6, 2026. The copyright holder for this preprint (which was not certified by peer review) is the author/funder. This article is a US Government work. It is not subject to copyright under 17 USC 105 and is also made available for use under a CC0 license.



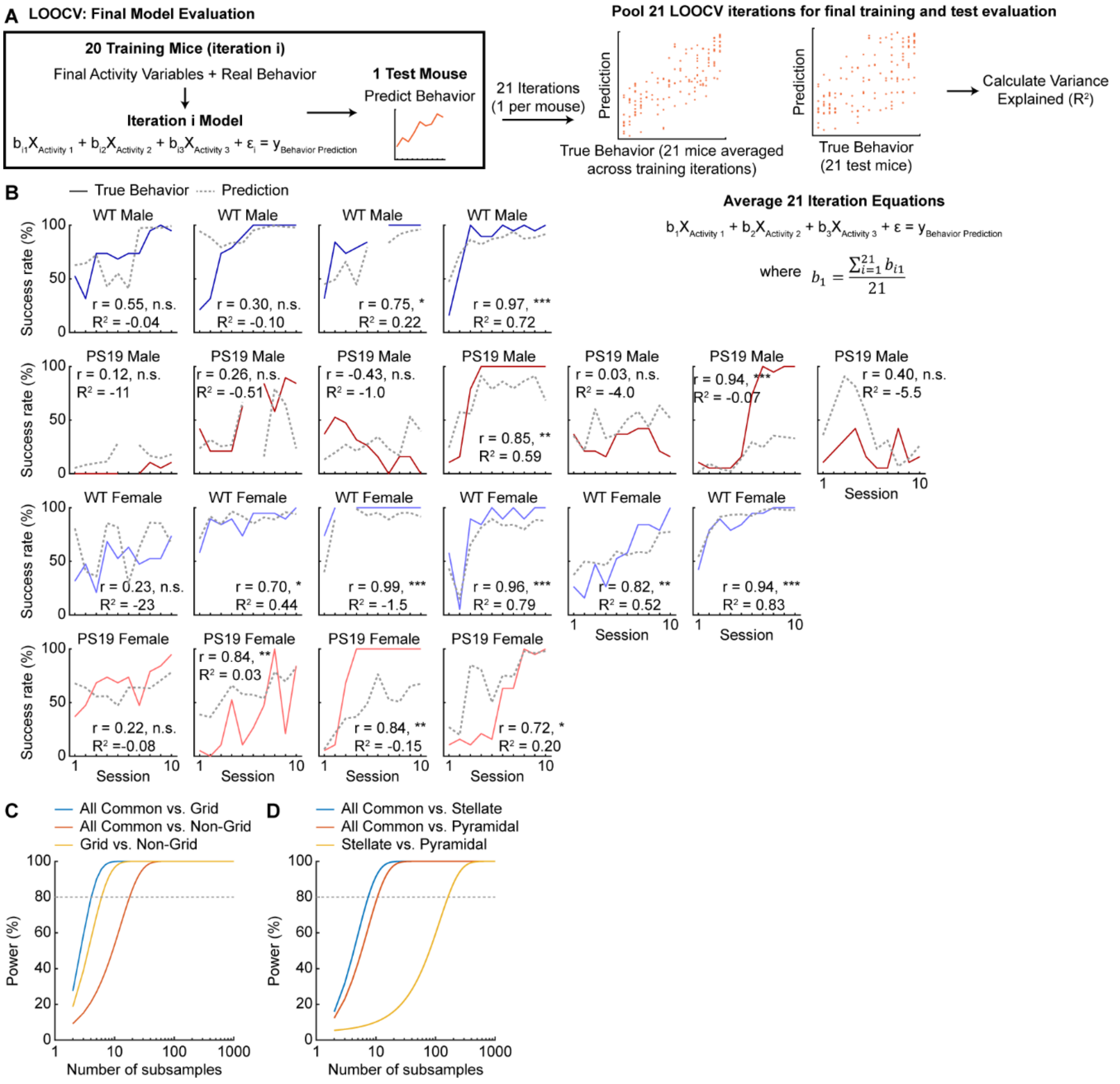


**Figure S11: MEC activity changes are predictive of behavioral deficits in PS19 mice.** Related to **Figure 7**.
**A.** Schematic of final model evaluation method.
**B.** Comparison of mean success rate (colored lines) and model predictions (dashed grey lines) across learning for individual mice (from top to bottom: WT male, PS19 male, WT female, PS19 female). Test predictions from single LOOCV iterations using the left-out mouse were used to calculate prediction.
**C.** Power analysis comparing the distributions of behavioral variance explained of the subsampled all-common-cell, grid cell, and non-grid cell activity data to determine the probability of obtaining a significant result if a true difference exists for different numbers of subsamples. Dashed line corresponds to 80% power (common vs. grid = 5 subsamples, common vs. non-grid = 18 subsamples, grid vs. non-grid = 7 subsamples).
**D.** Similar to **C**, but for all-common-cell, stellate cell, and pyramidal cell activity data (common vs. stellate = 8 subsamples, common vs. pyramidal = 11 subsamples, stellate vs. pyramidal = 162 subsamples).
$*p \leq 0.05$, $**p \leq 0.01$, $***p \leq 0.001$. Statistical details can be found in **Table S1**.

bioRxiv preprint doi: https://doi.org/10.64898/2025.12.30.696887; this version posted February 6, 2026. The copyright holder for this preprint (which was not certified by peer review) is the author/funder. This article is a US Government work. It is not subject to copyright under 17 USC 105 and is also made available for use under a CC0 license.



# METHODS

## Animals

All animal procedures were performed in accordance with animal protocol 1524 approved by the Institutional Animal Care and Use Committee (IACUC) at NIH/NINDS. PS19 mice[58] (B6;C3-Tg(Prnp-MAPT*P301S)PS19Vle/J, JAX stock #008169) were crossed with GP5.3 mice[67] (C57BL/6J-Tg(Thy1-GCaMP6f)GP5.3Dkim/J, JAX stock #028280). The F1 generation of this cross was used for all experiments. Mice positive for GCaMP and human P301S will be referred to as PS19 and mice positive for GCaMP but negative for human P301S will be referred to as wild-type (WT). The two-photon imaging experiments for environment learning used 11 PS19 mice (7 males and 4 females) and 10 WT mice (4 males and 6 females). These imaging mice ranged from 7.6-10.0 months old at the start of imaging. Mice for immunohistochemistry experiments included a subset of the imaging mice and additional mice. The additional histology mice did not necessarily express GCaMP. Detailed sex and age information can be found below for each histology analysis. Mice were maintained on a reverse 12-hr on/off light schedule with all experiments being performed in the light off period. Animals were house at a temperature of 70–74°F and 40–65% humidity.

<u>Paralysis Onset</u>

The age at which paralysis onset first occurred as determined by onsite veterinary staff was recorded for all F1 mice in the colony, after which mice were humanely euthanized. The age of death was recorded for all mice 5 months or older regardless of the cause of death. Living mice were also included in the analysis. Ages for deaths other than after hindlimb paralysis and for living mice were used as censored events. In total, 96 WT mice (44 females and 52 males) and 208 PS19 mice (103 females and 105 males) were included in this analysis.

## Rodent Surgeries

<u>Microprism Construction</u>

Microprism construction procedures were similar to those described previously[68,179]. A canula (MicroGroup, 304H11XX) was attached to a circular cover glass (3mm, Warner Instruments, 64-0720). A right angle microprism coated with aluminum on the hypotenuse (1.5mm, OptoSigma, RBP3-1.5-8-550) was then attached to the opposite cover glass side. All attachments were performed using UV-curing optical adhesive (ThorLabs, NOA81).

<u>Microprism Implantation Surgery</u>

Microprism implantation procedures were similar to those described previously[68,179]. Mice were anesthetized using a tabletop laboratory animal anesthesia system (induction: 3% isoflurane, 1 L/min oxygen, maintenance: 0.5–1.5% isoflurane, 0.7 L/ min oxygen, VetEquip, 901806) and surgery was performed on a stereotaxic alignment system (Kopf Instruments, 1900). A

bioRxiv preprint doi: https://doi.org/10.64898/2025.12.30.696887; this version posted February 6, 2026. The copyright holder for this preprint (which was not certified by peer review) is the author/funder. This article is a US Government work. It is not subject to copyright under 17 USC 105 and is also made available for use under a CC0 license.



homeothermic pad and monitoring system (Harvard Apparatus, 50-7220 F) was used to maintain a body temperature of 37 °C. After anesthesia induction, dexamethasone (2 mg/kg, VetOne, 13985-533-03) and saline (500 µL, 0.9% NaCl, McKesson, 0409-4888-50) were administered by intraperitoneal (IP) injection, and slow-release buprenorphine (1 mg/kg, ZooPharm, Buprenorphine SR-LAB) was administered subcutaneously. Enroflox 100 (10 mg/mL, VetOne, 13985-948-10) was used as an anti-microbial wash just after the skull was exposed and just prior to sealing the skull. All insertions were performed on the left hemisphere, aligning with previous observations of more favorable vasculature[179]. A 3 mm craniotomy was performed centered at 3.4 mm lateral to the midline and 0.75 mm posterior to the center of the transverse sinus (approximately 5.4 mm posterior to the bregma). A durotomy was then performed over the cerebellum. Mannitol (3 g/kg, Millipore Sigma, 63559) was administered by IP prior to the durotomy. The microprism was inserted into the transverse sinus and sealed to the skull with n-butyl cyanoacrylate tissue adhesive (Vetbond, 3 M, 1469SB). The exposed skull was also coated with Vetbond. A single-sided steel head plate for head fixation was mounted to the right side of the skull opposite the craniotomy. Finally, the prism and head plate were adhered to the skull with dental cement (Metabond, Parkell, S396).

**Virtual Reality Setup**

For all behavioral experiments, a customized virtual reality (VR) setup was used, which projects a one-dimensional (1D) virtual environment based on the running of a mouse, similar to that described previously[49,179]. Mice were head-fixed onto an air-supported polystyrene ball (8" diameter, Smoothfoam) using the mounted head plate. The ball rotated on an axle, allowing only forward and backward rotation. The virtual environment was projected onto a hemispherical dome filling the visual field of mice (270° projection). An optical flow sensor (Paialu, paiModule_10210) with infrared LEDs (DigiKey, 365-1056-ND) was used to measure the rotation of the ball and thereby control the motion of the virtual environment. The optical flow sensor output to an Arduino board (Newark, A000062), which transduced the motion signal to the computer controlling the VR. An approximately 4 µl water reward was provided via a lick tube using a solenoid. The solenoid was controlled using a Multifunction I/O DAQ (National Instruments, PCI-6229). The virtual environments were generated and projected using ViRMEn software (Princeton, version 2016-02-12)[180]. Imaging and behavior data were synchronized by recording a voltage signal of behavioral parameters from the VR system using the DAQ. ViRMEn environments were updated at 60 Hz. The DAQ input/output rate was 1 kHz. The synchronization voltage signal was updated at 20 kHz. Final behavioral outputs were matched to the imaging frame rate (15 Hz, see below) for synchronization.

Environments were colored blue and projected through a blue wratten filter (Kodak, 53-700) to reduce contamination of the imaging path with projected light. Virtual environments were 600 cm 1D linear tracks with patterned walls and patterned visual cues at fixed locations (**Fig. 1M**). At the end of the track, mice were immediately teleported to the start of the track.

bioRxiv preprint doi: https://doi.org/10.64898/2025.12.30.696887; this version posted February 6, 2026. The copyright holder for this preprint (which was not certified by peer review) is the author/funder. This article is a US Government work. It is not subject to copyright under 17 USC 105 and is also made available for use under a CC0 license.



## Immunohistochemistry

Mice were anesthetized with a ketamine (200mg/kg, VetOne, 13985-584-10) and xylazine (20mg/kg, VetOne. 13985-612-50) cocktail and were transcardially perfused with 4% paraformaldehyde (PFA, Electron Microscopy Sciences,15713) in phosphate buffer solution (PBS, Corning, 46-013-CM). Brain tissues were dissected and fixed in 4% PFA in PBS overnight at 4°C. Sagittal slices (40µm thick) were prepared using a VT1200S vibratome (Leica Biosystems). Slices were washed in PBS (4x 10 minutes) and then blocked with 10% Bovine serum albumin (BSA, Millipore Sigma, A3294), 0.5% triton X-100 (Millipore Sigma, T9284), and PBS for 1 hour at room temperature. Primary and secondary antibodies were diluted in 10% BSA, 0.5% triton, and PBS. Slices were incubated in diluted primary antibodies for 48 hours at 4°C, then washed in PBS (4x 10 minutes). Slices that required anti-goat secondary antibodies were incubated in diluted anti-goat secondary antibodies (1:500 dilution) for 1.5-2 hours at room temperature, washed in PBS (3x 10 minutes), incubated in other secondary antibodies (1:500 dilution) for 1.5-2 hours at room temperature, and washed in PBS (3x 10 minutes). Slices that did not require anti-goat secondary antibodies were incubated in the diluted secondary antibodies (1:500 dilution) for 1.5-2 hours at room temperature, then washed in PBS (3x 10 minutes). Slices were mounted with mounting medium (Vector Laboratories, H-1000-10). Primary antibodies used include goat anti-reelin (1:400, R&D Systems, AF3820), rabbit anti-Calbindin (1:500, Abcam, AB108404), and mouse monoclonal IgG1 anti-Phospho-Tau (1:1000, ThermoFisher Scientific, MN1020). Secondary antibodies included Alexa 405 conjugated donkey anti-goat antibody (Invitrogen, A48259), 568 conjugated goat anti-rabbit antibody (Invitrogen, A-11036), and Alexa 647 conjugated goat anti-mouse IgG1 antibody (Invitrogen, A-21240).

### Imaging

Histology slices were imaged using a Leica M165 FC microscope for the phospho-tau (pTau) accumulation with age quantification and using a Zeiss 880 spectral confocal for all other histology analyses. 3 to 4 image stacks were taken on the confocal microscope of the medial entorhinal cortex (MEC) along the dorsal-ventral axis and were stitched together using the "Pairwise stitching" plugin in ImageJ (Fiji, version 1.53q). 3-10 focal planes (Z-planes) were imaged for each image stack.

### Quantification of pTau Accumulation with Age

6 WT Males, 21 PS19 males, 6 WT females, and 11 PS19 females ages 2 to 10 months were stained in 3 staining batches. 16 of these mice were in the 2-photon imaging experiments. 1 Z-plane of the image stack was analyzed per slice. The dorsal 1.5mm region of MEC layer 2 was selected manually and the intensity of pTau immunostaining of the MEC layer 2 region was measured in ImageJ. A cutoff for pTau+ pixels in layer 2 was calculated for each of the three staining batches. The 99$^{th}$ percentile value of all the pixels in layer 2 was measured for each 2- and 3-month PS19 mouse in each of the staining batches. The cutoff for each batch was the average of these intensity values. The percentage area that was pTau+ was the number of pTau+ pixels above

bioRxiv preprint doi: https://doi.org/10.64898/2025.12.30.696887; this version posted February 6, 2026. The copyright holder for this preprint (which was not certified by peer review) is the author/funder. This article is a US Government work. It is not subject to copyright under 17 USC 105 and is also made available for use under a CC0 license.



the intensity cutoff divided by the total number of pixels in the MEC layer 2 region. The pTau intensity in the pTau+ region was the average pixel value in MEC layer 2 after subtracting the cutoff intensity and excluding negative pixel values.

Quantification of pTau Overlap with Reelin and Calbindin Cells

9 PS19 males and 5 PS19 females ages 8 to 10 months were stained in 4 staining batches. 10 of these mice were in the 2-photon imaging experiments. 2 Z-planes of the image stack were analyzed per slice. The dorsal 1.5mm region of MEC layer 2 and reelin+, calbindin+, and pTau+ cells inside this region were selected manually in ImageJ. Reelin and calbindin selections were overlaid with pTau+ cell selections. Reelin and calbindin selections were manually determined to be pTau+ by counting overlapping regions of interest (ROIs). pTau+ cells that overlapped with both reelin and calbindin selections were excluded when calculating the percentages of reelin+ and calbindin+ cells that are pTau+ and pTau intensity. The pTau intensity of pTau+ cells that overlapped with reelin+ or calbindin+ cells was calculated as the average pixel value after background subtraction within each Z-plane.

Neurodegeneration Quantification

4 WT Males, 7 PS19 males, 4 WT females, and 3 PS19 females, ages 8 to 10 months were stained in 4 staining batches. 17 of these mice were in the 2-photon imaging experiments. 1 Z-plane of the image stack was analyzed per slice. The dorsal 1.5mm region of MEC layer 2 and reelin+ and calbindin+ cells inside this region were selected manually in ImageJ. Cells with less than 90% of their area inside the layer 2 ROI were removed. Neurodegeneration was quantified by counting the number of reelin+ and calbindin+ cells and dividing by the area of the layer 2 region to get the number of cells per area.

**Behavior**

Training

Water-restricted mice were first trained in a 1 m environment with a single reward at a fixed track location during acclimation to VR to encourage running. Once mice began to run (typically 50-100 laps/hour for two days), they were transitioned to a 6m environment with a single water reward at a fixed track location (**Fig. 1M**, passive FE). Mice were trained in the passive FE until they started to develop reward-predictive behavior as measured by predictive licking and predictive slowing (roughly 50% predictive licking and 60% predictive slowing, see “Predictive Licking” and “Predictive Slowing” for quantification), which has been shown previously to demonstrate familiarity with an environment[49,181]. Both WT and PS19 mice were able to reach these criteria. Mice then began training in an active task (active FE). The active FE was visually identical to the passive NE, but mice only received water reward if they stopped in an unmarked 50 cm reward zone centered at the reward location of the passive FE. A stop was defined as a velocity below 1 cm/s for at least 1 s. Mice could only receive one reward per lap. For the first 3-

bioRxiv preprint doi: https://doi.org/10.64898/2025.12.30.696887; this version posted February 6, 2026. The copyright holder for this preprint (which was not certified by peer review) is the author/funder. This article is a US Government work. It is not subject to copyright under 17 USC 105 and is also made available for use under a CC0 license.



4 days, mice underwent manual training, consisting of manually stopping the mouse in the reward zone. When a mouse could stop on its own, it was further trained without manual stopping with a shortened stop period (0.5s). Once a mouse stopped on its own with the shortened stop period, the full 1s stop period was applied. Additional manual training was performed periodically as necessary to reinforce correct behavior.

Mice were trained in the active FE until they learned the active task (success rate >~75%, see "Success/Attempt Rate" for quantification) and ran well in the active FE environment (>~20 laps per hour). Because there was no a-priori assumption that PS19 mice would be able learn the active spatial task, mice (WT or PS19) that did not show learning of the active task were transitioned when they had been trained in the active task for a similar number of days to the average WT mouse (>~17 days). Mice then underwent several days of baseline imaging in the active FE environment, until their behavior recovered to its pre-imaging level. Further behavioral analysis and analysis of neuronal activity used the FE imaging day with the highest success rate in order to ensure a baseline day representing neuronal activity in a well-learned environment. Mice were then imaged for 10 consecutive days in the novel environment (NE), which had a different arrangement of visual cues with different patterns and a 50cm reward zone in a different location (**Fig. 1M**). Mice were restricted to 20 laps per day in the NE. Because imaging could not be performed for the entirety of the first lap, the first lap was ignored for behavioral analysis as well.

Success/Attempt Rate

Success rate was measured as the percentage of laps that a mouse stopped in the reward zone. The success rate criteria used for analysis were identical to those used to determine when the mouse received reward (1 s with velocity less than 1 cm/s). To measure attempts to receive reward, we conducted a post-hoc analysis in which we relaxed the criteria for a successful stop in reward zone. Specifically, we simultaneously expanded the reward zone (from 50 cm to 100cm), increased the speed threshold defining a stop (from 1 cm/s to 5 cm/s), and reduced the amount of time required for a stop (from 1 s to 0.5 s).

Predictive Licking

Predictive licking (PL) was measured as the percentage of licks that occurred within a specific distance threshold (20 cm) prior to the reward delivery relative to all other locations (excluding 30 cm after reward). PL was only calculated for the passive FE to determine the transition to active learning.

Predictive Slowing

To calculate predictive slowing (PS), for each lap, the velocity changes between each data point were calculated along the track. The pre-reward acceleration value was calculated as the mean velocity changes ($\Delta v$) within a 90 cm window before the reward (for example 175 cm to 265 cm for a reward location at 265 cm). The rest of the track was analyzed using a rolling average of $\Delta v$ for the same window size at 1 cm intervals, generating a series of comparison acceleration

bioRxiv preprint doi: https://doi.org/10.64898/2025.12.30.696887; this version posted February 6, 2026. The copyright holder for this preprint (which was not certified by peer review) is the author/funder. This article is a US Government work. It is not subject to copyright under 17 USC 105 and is also made available for use under a CC0 license.



values. The slowdown percentile for a given lap was the percentile of the pre-reward acceleration within the comparison acceleration values from the rest of the track, such that a higher percentile signified a more negative acceleration (i.e., deceleration) and thus better PS. The rolling averages calculated for the comparison values excluded any window intersecting the edge of the track or areas close to the reward (from 90 cm before to 30 cm after reward delivery) to avoid edge effects and reward related behavior, respectively. Thus, the comparison windows ranged from 0–90 cm to 85–175 cm and 295-485 to 510-600 on the passive FE. The PS of a session was computed as the average slowdown percentile of all the laps within the session. PS was only calculated for the passive FE to determine the transition to active learning.

Global Discrimination Index

A global discrimination index (global d') was calculated to measure the ability of a mouse to discriminate between the reward zone and other locations. The success rate was compared to the percentage of laps with stops in a rolling 50 cm window of track positions (the same size as the reward zone). For each comparison window an individual local d' value was calculated as d' = norminv(HR)-norminv(FA); where HR=Hit Rate or success rate in the reward zone, and FA=False Alarm Rate or the percentage of laps with a stop in a given comparison zone[85,86], and norminv() is the inverse function of the cumulative Gaussian distribution. HR and FA were constrained between 1/2N and 1-1/2N, where N is the number of laps, because norminv is undefined for 0 or 1. Global d' is the average d' for all comparison windows. A threshold of d' >1 was defined as the threshold for successful learning[85-87]. If mice never reached a global d' of 1, the number of days to reach the learning threshold was considered to be 11 (total number of NE days + 1) as a penalty.

Mean Velocity

The mean velocity of a mouse for a given session was defined as the average velocity of all time points where the mouse was moving faster than 1 cm/s. This value was used for both analyzing mouse behavior and as a potential predictor variable for the general linear models predicting behavior.

Number of Stops per Session

The number of stops in a given session was calculated as the number of distinct instances when the velocity of a mouse went below 1 cm/s for at least 1 s. A given stop could be arbitrarily long and continued until the velocity rose above 1 cm/s again. Therefore, two stops could be close together in space and time but were required to have at least some period of higher velocity separating them.

Velocity Matching

To perform velocity matching, pairs of mice with similar velocity were matched on each day. For each sex group (all, male, female), the optimal set of cross-genotype mouse pairs was determined using the MATLAB (Mathworks, v2023b) matchpairs function, which finds the

bioRxiv preprint doi: https://doi.org/10.64898/2025.12.30.696887; this version posted February 6, 2026. The copyright holder for this preprint (which was not certified by peer review) is the author/funder. This article is a US Government work. It is not subject to copyright under 17 USC 105 and is also made available for use under a CC0 license.



combination of pairs with the minimum total cost (velocity difference). All possible pairs of velocity difference between a WT and PS19 mice were initially calculated, but the cost of any pairs with a velocity difference greater than 5 cm/s was set to infinity. In addition, the cost of unpaired mice was set to $10^6$ (much higher than the velocity values). This method finds the maximum possible number of mouse pairs with a velocity difference less than 5 cm/s. The velocity-matched velocity, success rate, and global d' were then calculated using only the mice that were part of a valid pair on each day.

Behavior Fitting

To calculate the rate of spatial learning, the mean success rate of each group was fitted with a one-phase association exponential function (Prism 9.3.1, GraphPad), where y is the success rate and x is day (NE day 1 to 10). For each sex group (all, male, female), we first determined if both WT and PS19 mice had the same baseline ($Y_0$) and plateau ($P$) success rate using an extra sum-of-squares F test. This analysis found that for all sex groups, fitting the two genotypes with the same baseline and plateau values was preferred to fitting the genotypes separately (Extra sum-of-squares F test. All mice: $Y_0$=8.0, $P$=94, p=0.63. Male mice: $Y_0$=12, $P$=100, p=0.13. Female mice: $Y_0$=0.0, $P$=90, p=1). We then re-fit each genotype with the baseline and plateau levels set at the previously determined mutual values and let K, the learning rate constant, vary. We again used an extra sum-of-squares F test to determine whether the genotypes were better fit with the same or different values of K. If the F test determined that the values of K are different, we could conclude that the genotype with the higher rate constant learned faster. Once the rate constant was derived, we calculated the number of days required to achieve 95% of the plateau ($T_{95\%}$) using the equation $T_{95\%} = \ln(20/K)$. The $T_{95\%}$ directly corresponds to K but is more interpretable.

**Two-Photon Imaging**

Imaging was performed using an Ultima 2Pplus microscope (Bruker) configured with the above VR setup. A tunable laser (Coherent, Chameleon Discovery NX) set to a 920 nm excitation wavelength was used. Laser scanning was performed using a resonant-galvo scanner (Cambridge Technology, CRS8K). GCaMP fluorescence was isolated using a bandpass emission filter (525/25 nm) and detected using GaAsP photomultiplier tubes (Hamamatsu, H10770PB). A 16x water-immersion objective (Nikon, MRP07220) was used with ultrasound transmission gel (Sonigel, refractive index: 1.3359[182]; Mettler Electronics, 1844) as the immersion media.

The anterior-posterior (AP) and the medial-lateral (ML) angle of the prism (i.e., the angle of the surface of the prism along to the AP or ML direction of the mouse) relative to the head-fixed position of the mouse were measured prior to the first imaging session. The head plate holder and rotatable objective angles were set daily to align the objective with the prism in the AP and ML direction, respectively, such that the objective was parallel to the prism surface. Black rubber tubing was wrapped around the objective and imaging window to prevent light leakage into the objective.

bioRxiv preprint doi: https://doi.org/10.64898/2025.12.30.696887; this version posted February 6, 2026. The copyright holder for this preprint (which was not certified by peer review) is the author/funder. This article is a US Government work. It is not subject to copyright under 17 USC 105 and is also made available for use under a CC0 license.



Microscope control and image acquisition were performed using Prairie View software (Bruker, version 5.7). Raw data was converted to images using the Bruker Image-Block Ripping Utility. Imaging was performed simultaneously on two fields-of-view (FOV) using an electrotunable lens (ETL, Bruker). FOV were 750 µm x 750 µm and were separated by 35-43 µm in the z-plane. Each FOV was imaged at 15 Hz with 512 × 512 resolution. Average beam power at the front of the objective was typically 100–120mW. Imaging and behavior data were synchronized as described above. Only the activity on laps where imaging was acquired for the full lap was used for analysis. Due to technical issues, four mice were not successfully imaged for one NE day. These four sessions are included in the behavioral analysis but are excluded from the imaging and modeling analysis. To ensure comparable common cell identification (see "Cell Alignment"), an additional NE imaging day was used for the alignment of these mice, such that all mice were aligned with 11 imaging days.

**Image Processing:**

Imaging data underwent a consecutive 2-frame rolling average and was processed as previously described using published MATLAB scripts[68]. Motion correction was performed using cross-correlation based, rigid motion correction. Identification of regions of interest (ROIs, active cells) with correlated fluorescence changes and extraction of the fluorescence time course of individual ROIs was performed using Suite2p (v0.11.1)[183]. The fractional change in fluorescence with respect to baseline (ΔF/F, referred to as activity level) was calculated as (F(t) – F0(t)) / F0(t)[179]. For each cell, significant calcium transients were identified using amplitude and duration thresholds, such that the false-positive rate of significant transient identification was 1%[184]. A final ΔF/F including only the significant calcium transients (as opposed to the raw ΔF/F) was used for further analysis, except as noted. A speed threshold was calculated by generating a 100-point histogram of all instantaneous velocities greater than 0 and taking the value twice the center of the first bin (approximately 1% of max positive speed). The ΔF/F points when the mouse was moving below the speed threshold were excluded from the following analyses. The mean ΔF/F (significant transients only, as mentioned above) for a cell was calculated as a function of position along the track in 5 cm bins.

<u>Cell Alignment</u>

All imaging sessions for a given mouse were aligned pairwise using an established probabilistic modeling method[185] to identify common cell pairs between each pair of sessions. We first performed automatic pairwise rigid registration of the imaging sessions to identify common neuron pairs between pairs of imaging days. Manual registrations were applied to the imaging session pairs with failed automatic alignment, but common cell pairs were still identified using the same probabilistic modeling method. Pairwise registration results were combined to determine the common neurons across all 11 days of the spatial learning task. Due to the pairwise nature of the registration, a neuron could be aligned to different neurons on different days. Manual examination

bioRxiv preprint doi: https://doi.org/10.64898/2025.12.30.696887; this version posted February 6, 2026. The copyright holder for this preprint (which was not certified by peer review) is the author/funder. This article is a US Government work. It is not subject to copyright under 17 USC 105 and is also made available for use under a CC0 license.



was used to select the optimal alignment results that display the highest level of anatomical footprint consistency across all imaging days and to exclude incorrect alignments.

## Data Analysis:

### Stellate/Pyramidal Cell Classification

Cells were classified as stellate cells or pyramidal cells based on the bimodal distribution of their area similar to a previously described method[68]. Because previous studies used long-axis diameter of the cells instead of area, we first validated this method using histology slices of cells expressing GCaMP6f that were stained for reelin (a stellate cell marker) and calbindin (a pyramidal cell marker). Cells that expressed both GCaMP6f and either reelin or calbindin, but not both, were manually traced and their area was calculated. The valley between the peaks of the cell area was 151 $\mu m^2$. To increase the confidence of cell classification, cells with areas smaller than 131 $\mu m^2$ and larger than 171 $\mu m^2$ (151 ± 20 $\mu m^2$) were classified as pyramidal and stellate cells, respectively. We calculated the true positive rate of stellate and pyramidal cells as the number of cells identified as stellate or pyramidal cells, respectively, divided by the total number of cells identified as stellate or pyramidal cells (stellate cells: 99% true positive rate, pyramidal cells: 90% true positive rate).

For the imaging FOV, cells were manually traced from motion corrected maximal projections of each FOV on the FE imaging day. The valley between the two peaks of cell area was 158 $\mu m^2$. To increase the confidence of cell classification, cells with areas smaller than 138 $\mu m^2$ and larger than 178 $\mu m^2$ (158 ± 20 $\mu m^2$) were classified as pyramidal and stellate cells, respectively.

### Matching Manually Identified Cells to Active Cells

To identify common cells as stellate or pyramidal cells, the ROIs of the active cells on the FE day were overlaid with the manually traced ROIs. An active cell and a manually drawn cell were determined to be the same cell if the centroid distance between the two ROIs was less than 4 µm and the area of the intersect of the ROIs divided by the area of the union of the ROIs (IoU) was less than 0.4. In the event that a given cell matched with multiple cells, the cell pair with the smallest centroid distance was used. All common cells that matched a manual cell on the FE day were assigned that identity across all days. Because not every active cell matched with a manual cell and not every manual cell was identified as stellate or pyramidal, the population of common cells identified as stellate or pyramidal cells is only a subset of the total common cell population.

### Spatial Field Identification:

To calculate significant spatial firing fields, regions of the track with significantly consistent activity, mean $\Delta F/F$ was compared to shuffles of the original $\Delta F/F$ as described previously[68]. Mean $\Delta F/F$ for a cell was spatially binned along the track in 5 cm bins and averaged across laps. A gaussian window of 3 spatial bins was applied to the averaged $\Delta F/F$ trace to smooth the data. Spatial fields were identified by comparing the amplitude of the original smoothed $\Delta F/F$

bioRxiv preprint doi: https://doi.org/10.64898/2025.12.30.696887; this version posted February 6, 2026. The copyright holder for this preprint (which was not certified by peer review) is the author/funder. This article is a US Government work. It is not subject to copyright under 17 USC 105 and is also made available for use under a CC0 license.



with a random ΔF/F distribution created by 1000 bootstrapped shuffled responses. Each bootstrapped shuffled response was generated by rotating the ΔF/F trace of the whole session so that for every time point of the recording, its track position was preserved but its calcium response was changed. The ΔF/F trace was rotated by starting the trace from random sample numbers chosen from the interval 0.05 × Nsamples to 0.95 × Nsamples, where Nsamples was the number of samples in the ΔF/F trace. A shuffled mean ΔF/F was calculated for each rotation. For each 5 cm bin, the pvalue equaled the percentage of shuffled mean ΔF/F that was greater than or equal to the real mean ΔF/F. Therefore, 1 - pvalue equaled the percentage of shuffled mean ΔF/F lower than the real mean ΔF/F. A spatial field was defined as a region of at least 3 consecutive 5 cm bins (except that the fields at the beginning and end of the track could have 2 bins) that had a mean ΔF/F higher than at least 80% of 1000 shuffles at the corresponding bins (1 - pvalue ≤ 0.2). Additionally, at least 20% of laps were required to have a significant calcium transient in the spatial field.

## Cue Cell Classification

To identify cue cells, cue scores of each cell on a specific day session were calculated based on a previously published method[36]. The activity of the cell was first shifted to best match a cue template, which contained ones and zeros representing track areas with and without cues, respectively. A cue zone was identified for each cue by including the cue itself and the surrounding region expanded by half of the cue width on both sides. The correlations between shifted activity and cue template within individual cue zones were calculated and were further averaged as the cue score of the cell. 200 shuffled cue scores were generated for each cell using the same calculation but by randomizing the cue location in the cue template. Shuffled cue scores from all cells across all FOVs and imaging days for a given environment were pooled to generate an overall cue score distribution. A cell was identified as a cue cell if its cue score on the day was above the 90th percentile of the shuffle cue score distribution. While previous studies used a 95th percentile threshold[36,49,68], a 90th percentile threshold was used here to better identify cells with cue-matched activity. Cue scores of cells were calculated toward left cues, right cues, and cues on both sides of the track. A cue cell could be identified based on any cue type. Cue cell identification was performed each day to exclude cue cells from the putative grid cell population (see "Grid Cell Classification"). Common cells were identified as true cue cells if they were identified as cue cells of any type (left, right, or both sides) on more than half of imaging days (≥ 6).

## Grid Cell Classification

Grid cells were identified within each recording day based on the following criteria. (1) Grid cells need to have at least two spatial fields on the track. (2) Each grid cell must have more than L/(5w) transitions between in-field and out-of-field periods, where L represents track length and w represents mean field width of the grid cell's response. (3) The widest field of the response must be smaller than 5w. (4) At least 30% of the bins must be assigned to either in-field or out-of-field periods. (5) The ratio between in-field and out-of-field mean ΔF/F needs to be ≥ 2[68,92,93]. (6)

bioRxiv preprint doi: https://doi.org/10.64898/2025.12.30.696887; this version posted February 6, 2026. The copyright holder for this preprint (which was not certified by peer review) is the author/funder. This article is a US Government work. It is not subject to copyright under 17 USC 105 and is also made available for use under a CC0 license.



The cell must not be identified as a cue cell on that day. Common cells were identified as true grid cells if they were identified as grid cells on more than half of imaging days (≥ 6). All cells that were not identified as grid cells were considered non-grid cells. Non-grid cells are distinct from unclassified cells (see "Unclassified Cell Identification")

Speed Score/Speed Cell Classification

Speed score was calculated as previously described[37,38]. First, spatial position for the full session was smoothed using a 0.8s gaussian filter. Velocity was calculated using the smoothed positions as the difference in position divided by the difference in time. Time points with velocity less than zero or greater than 100 cm/s were ignored in the rest of the calculation. A histogram of speed was then used to determine the largest 10 cm/s speed range (0-10 to 90-100) that the mouse occupied for at least 30 s, and the mean velocity of time points with speed in the range was used as a new upper speed limit. A new lower limit of 6 cm/s was also used. If the new upper limit was lower than 6 cm/s, the upper limit was adjusted using the averaged velocity of the next 10 cm/s speed bin. Time points with velocity outside these new limits were ignored in the rest of the calculation. The temporal ΔF/F trace was smoothed with a 0.4s gaussian filter. Raw speed score is defined as the Pearson correlation between velocity and ΔF/F for time points with velocity in the final range. For calculating positive and negative speed score components, classification was performed daily, so a given cell could contribute to the positive speed score component on one day and the negative speed score component on another. Absolute speed score is the absolute value of the raw speed score.

1000 shuffled speed scores were generated for each cell using the same calculation but the temporal ΔF/F trace was rotated as described above (see Spatial Field Identification). Shuffled speed scores from all cells across all FOVs and imaging days were pooled to generate an overall speed score distribution. A cell was identified as a speed cell if its speed score on the day was above the 99th percentile (positive speed cell) or below the 1$^{st}$ percentile (negative speed cell) of the shuffle cue score distribution. Speed cell identification was performed on each day. Common cells were identified as true speed cells if they were identified as speed cells of either type (positive or negative) on more than half of imaging days (≥ 6).

Unclassified Cell Identification:

Unclassified cells were all common cells that were not identified as cue, grid, or speed cells. Note that cells could not be classified as both a cue cell and a grid cell, but cells could be classified as a speed cell and either a cue cell or a grid cell. Therefore, the combined number of cue, grid, speed, and unclassified cells could be greater than the total number of common cells.

Within-Day Consistency

The within-day consistency for a given cell in a particular imaging session was calculated as previously described[98]. The spatially binned mean ΔF/F for each lap along the track was

bioRxiv preprint doi: https://doi.org/10.64898/2025.12.30.696887; this version posted February 6, 2026. The copyright holder for this preprint (which was not certified by peer review) is the author/funder. This article is a US Government work. It is not subject to copyright under 17 USC 105 and is also made available for use under a CC0 license.



correlated with that of every other lap. The average of these correlations is the within-day consistency value for a given cell.

Cross-Day Consistency

The activity matrix correlation (cross-day consistency) for a given set of cells in a particular imaging session was calculated as previously described[49]. The spatially binned mean ΔF/F for each cell was averaged across all laps along the track, generating a 1D array. The calculated array for each cell was concatenated to generate a single matrix (size = total number of cells by number of spatial bins) such that the activity of a given tracked cell was in the same row of the activity matrix for all days. The day-to-day activity matrix correlation was calculated on a per cell basis as the 1D Pearson correlation between the generated 1D arrays for a given cell on two consecutive days.

Inter-Mouse Consistency

To calculate inter-mouse consistency, a spatial map for each mouse on a given day or set of days was calculated. The spatially binned mean ΔF/F for all common cells from both FOV from a given mouse were averaged to generate a spatial map for each day. The daily maps (NE days 7-10) were averaged together to generate the late-learning spatial map for each mouse. Inter-mouse consistency between a pair of mice was defined as the correlation between the daily or late-learning maps of those mice. Within-group and between-group comparisons were those between two mice of the same or different sex/genotype, respectively, as described in the text. To calculate the difference between WT and PS19 mice for the sex versus molecular cell type heatmap and to calculate the values used for the general linear models predicting behavior, the correlations of each mouse with every other mouse were averaged to generate a single value per mouse per day or per set of days.

Position Decoding

Position decoding was performed by separating the imaging data into template data (odd laps) and testing data (even laps) as described previously[49,98]. For this analysis, the raw ΔF/F (not only significant transients) was used because performing the decoding analysis with the significant transient ΔF/F resulted in high decoding accuracy at the ends of the track. Because we were interested in decoding as a function of track position, we used raw ΔF/F rather than excluding the edge of the track. The spatially binned (5 cm bins) mean ΔF/F for the template data laps were averaged across laps and concatenated for all cells to generate a template matrix (size = total number of cells by number of spatial bins). The ΔF/F of all cells at each time were then correlated to each spatial bin of the averaged template data matrix. The decoded position for a given time point was the spatial position of the template bin that gave the highest correlation. Decoding accuracy was the percentage of correctly decoded track positions, which was within 2 spatial bins from the spatial bin of the real position. The first and last spatial bin of the track were considered adjacent for this calculation. The decoding was performed with 100 random samples of 30 cells for each FOV. All cells or all cells identified as grid cells or non-grid cells on a given day, were

bioRxiv preprint doi: https://doi.org/10.64898/2025.12.30.696887; this version posted February 6, 2026. The copyright holder for this preprint (which was not certified by peer review) is the author/funder. This article is a US Government work. It is not subject to copyright under 17 USC 105 and is also made available for use under a CC0 license.



used for this analysis due to the small number of common cells in some FOV. 30 cells were chosen for the analysis to maximize the number of FOV with sufficient cells (>~90% of FOV had 30 cells for each cell category. All Cells: 412/412 FOV had 30 cells, Grid Cells: 366/412 FOV had 30 cells, Non-Grid Cells: 403/412 FOV had 30 cells). This analysis could not be separately conducted on stellate or pyramidal cells because many FOV had too few cells in a particular cell category.

<u>Spatial Activity Cue Anchoring and Decoding Cue Anchoring</u>

The spatially binned mean ΔF/F for all common cells from a given FOV were averaged to generate a spatial map for each day (spatial activity level). This calculation was performed per FOV to account for the noise in this measurement. Spatial position decoding was defined as the decoding accuracy as described above averaged for test bins at each spatial position. This resulted in a 1D array representing position decoding as a function of track position (spatial decoding).

For spatial activity level and spatial decoding, in-cue regions were defined as the average of all spatial bins within an expanded cue region (the cue itself and the surrounding region expanded by half of the cue width on both sides). Out-cue regions were defined as the average of all spatial bins not part of the in-cue region. The full track decoding was the average spatial decoding across the full track. Spatial activity cue anchoring and decoding cue anchoring were defined as the in-cue/out-cue (I/O) ratio, or the in-cue value divided by the out-cue value.

<u>General Linear Model</u>

*Model Inputs*

As described in the text, the predictor variables included behavioral variables (mean velocity), general activity variables (activity level, spatial selectivity, raw speed score, and absolute speed score), map consistency variables (within-day consistency, cross-day consistency, and inter-mouse consistency), and global representation variables (spatial activity cue anchoring and decoding cue anchoring). For activity variables calculated by FOV (spatial activity cue anchoring and decoding cue anchoring), the average activity value of the two FOVs for the given cell type was used as the input for a given day. For activity variables calculated by mouse (inter-mouse consistency, as described above), the daily activity value was used directly. For activity variables calculated by cell (all others), the average activity of all cells for a given cell type from both imaging FOV was used as the input for a given day. As described above, decoding cue anchoring was calculated using all cells rather than common cells and could not be calculated for stellate and pyramidal cells. Because all other activity variables were calculated using common cells, the model types will be referred to as all-common, grid, non-grid, stellate, and pyramidal even though decoding cue anchoring was calculated using all cells. Decoding cue anchoring was not used as a predictor variable when comparing the stellate and pyramidal models to the all-common-cell model. Sex and genotype were included as confounding variables. Mouse identity and session number were not included in the model. All predictor variables were standardized using z-score normalization.

bioRxiv preprint doi: https://doi.org/10.64898/2025.12.30.696887; this version posted February 6, 2026. The copyright holder for this preprint (which was not certified by peer review) is the author/funder. This article is a US Government work. It is not subject to copyright under 17 USC 105 and is also made available for use under a CC0 license.



All modeling was performed using a general linear model (GLM)[101] using the MATLAB stepwiseglm and fitglm functions. Because success on each lap was binary and all mice experienced 19 laps that were used for behavioral analysis each NE day, the GLM always used a binomial distribution with a binomial size of 19.

*Variable Selection*

Variable selection was performed by iteratively removing predictor variables from a full model using the MATLAB stepwiseglm function to eliminate predictor variables that did not provide significant contributions to the model. This was performed only for the all-common-cell data. The initial model formula included all linear terms with no interactions, so all predictor and confounding variables were included. The input for the upper bound of the model was also all linear terms with no interactions to prevent nonlinear or interaction terms being added. The input for the lower bound of the model included just the confounding variable terms to prevent these variables from being removed. The maximum number of steps was set so that at least one predictor term would always remain.

This procedure was performed using leave-one-out cross-validation (LOOCV)[186] across mice. Each iteration, 20 mice were used for training and the one left-out mouse was then used for testing. A total of 21 iterations were performed so that each mouse served as the left-out mouse once, and 21 sets of variables were obtained from the iterative removal process. A final variable set was selected using only those variables that were present in all 21 post-removal variable sets. The same procedure was repeated but excluding sex and genotype to determine whether the confounding variables affected variable selection.

To test the validity of this iterative removal process, the same procedure was performed when the success rate was shuffled across mice and days to remove the correlation between activity and behavior. For both the real data and the shuffled data, the predictions for the test mouse from all 21 iterations were combined to generate a final combined prediction. An $R^2$ goodness of fit (variance explained) was calculated between the predicted behavior and the real or shuffle behavior. A total of 1,000 shuffles of success rate were performed to generate a distribution of variance explained in the absence of any connection between the predictors and success rate. The p-value of the iterative removal method was defined as $(R^2_{greater} + R^2_{equal}/2)/(nShuffle + 1)$, where $R^2_{greater}$ is the number of shuffle $R^2$ values greater than the real data $R^2$ value, $R^2_{equal}$ is the number of shuffle $R^2$ values equal to the real data $R^2$ value, and nShuffle is the number of shuffles performed[102].

*Final Model Training and Evaluation*

To evaluate the selection of the final variables determined above and the predictive ability of these variables, a final model was trained on only the final variable data using the MATLAB fitglm function. LOOCV was again performed for this analysis and those described below in the same manner as above. The final model coefficients were the average coefficients of the 21 LOOCV iteration models.

bioRxiv preprint doi: https://doi.org/10.64898/2025.12.30.696887; this version posted February 6, 2026. The copyright holder for this preprint (which was not certified by peer review) is the author/funder. This article is a US Government work. It is not subject to copyright under 17 USC 105 and is also made available for use under a CC0 license.



To evaluate the final variable selection, this final model was compared to sub-models trained with each of the final variables removed one at a time and to expansion-models with each of the variables not in the final model added one at a time[187]. The training $R^2$ goodness of fit is calculated for the final model, each sub-model, and each expansion-model by averaging the training predictions of each mouse across the 20 LOOCV iteration that it was a training mouse, combining these averaged training predictions across mice, and comparing to the true behavior.

To directly evaluate the final model fit, a testing $R^2$ goodness of fit was calculated by combining the predictions for the test mouse from all 21 iterations and comparing to the true behavior. To evaluate the ability of the model to predict learning behavior, the prediction time courses of individual mice or groups were compared to their true behavior. Individual mouse prediction time courses were the test prediction on the LOOCV iteration when that mouse was the test mouse. The prediction time course for each group was calculated as the average of the test predictions across all mice in that group. The true behavior time course for each group was the average success rate across all mice in that group.

*Dominance Analysis*

To evaluate the relative contribution of each of the final variables to the final model prediction, dominance analysis was performed[103]. We determined the effect of removing each variable from the full model and from all possible sub-models to determine the average contribution of each variable[103]. LOOCV is again used, with each mouse being used as the test mouse once. For each LOOCV iteration, the full model and every possible sub-model are trained using the MATLAB fitglm function. In other words, a GLM is trained using every possible combination of the variables (activity level, spatial selectivity, absolute speed score, within-day consistency, decoding cue anchoring, and spatial activity cue anchoring, sex, and genotype).

To evaluate the contribution of a given variable, the training $R^2$ goodness of fit of all sub-models with this variable are compared to the smaller sub-models where this variable is removed. For example, if a full model has variables A, B, and C, the contribution of the variable A is made by comparing the model ABC to BC, AB to B, AC to C, and A to the coefficient 1. A tier is defined as all sub-models with a specific number of variables (i.e. models with only two variables). The comparisons made for all sub-models in a tier are averaged together to generate a final tier $R^2$ value. For example, tier 2 of the 3 variable model has the possible sub-models AB and AC when evaluating the contribution of the variable A. Therefore, the tier 2 $R^2$ value for the variable A would be the average of the comparisons for AB to B and AC to C.

A full model with N variables will have N tiers of $R^2$ values for each variable. The final variance explained for a variable is the average of the $R^2$ values for all N tiers. The LOOCV iterations are combined by averaging the final variance explained for each variable across iterations. The sum of the variance explained across all variables is roughly equal to variance explained by the full model[103], and can therefore be thought of as the relative contribution to the final variance explained.

bioRxiv preprint doi: https://doi.org/10.64898/2025.12.30.696887; this version posted February 6, 2026. The copyright holder for this preprint (which was not certified by peer review) is the author/funder. This article is a US Government work. It is not subject to copyright under 17 USC 105 and is also made available for use under a CC0 license.



*Model Input Subsampling*

Activity data was subsampled to match the number of cells that contributed to the activity in each mouse across cell types (all-common vs. grid vs. non-grid or all-common vs. stellate vs. pyramidal). The minimum number of cells across cell types was determined for each mouse. For each subsample iteration (N = 200 iterations), that number of cells minus 1 were randomly selected across the two FOV for that mouse and the activity parameters of only those cells were calculated. Because decoding cue anchoring already used 30 cells per FOV, the original values of this variable were used. The above procedure ("Final Model Training and Evaluation") was used to generate an $R^2$ variance explained for the test predictions of each subsample iteration for each cell type. Power analysis to determine the power of the difference between groups (the probability of obtaining a significant result if a true difference exists) if the number of subsamples was varied was performed using the MATLAB sampsizepwr function assuming a two-sample pooled t-test. No additional exclusions were made comparing the all-common, grid, and non-grid models. As mentioned above, decoding cue anchoring was not used for the comparison of the all-common, stellate, and pyramidal models. In addition, because several mice had very few stellate or pyramidal cells, mice with fewer than 3 cells were excluded from the analysis.

*Genotype Classification:*

To classify mouse genotype based on the behavior predictions of MEC neuronal activity, the above procedure ("Final Model Training and Evaluation") was used to generate test predictions for each cell type. Because these results were not compared across cell types, no subsampling was performed. Instead all common cells or all grid, non-grid, stellate, or pyramidal cells among the common cell population were used (except for decoding cue anchoring, which used all cells, all grid cells, or all non-grid cells). As above, decoding cue anchoring was not used for the stellate or pyramidal cell models. In addition, sex and genotype were not included to avoid biasing the classification of genotype. Mice with fewer than 3 cells of a given cell type were excluded from the analysis of that cell type.

The average test prediction across late learning (days 7-10) of each mouse was classified as WT or PS19 based on whether it was closer to the average true behavior of WT or PS19 mice of that sex. In the rare event of a tie, classification was considered half correct (including a half contribution to the classification accuracy below). Each mouse was excluded from the average true behavior calculations for its own test prediction, except for in the pyramidal cell model, which used all mice to calculate the average true behavior due to the smaller number of mice included in the calculation. The classification accuracy of a given model for a given sex was the percentage of mice of both genotypes combined that were correctly classified. An identical shuffle procedure was used to that described above (200 shuffles, see "Variable Selection") to generate a shuffle distribution of genotype classification accuracy. The percentile of the classification of real predictions relative to the shuffles was reported.

**Activity Heatmaps**

bioRxiv preprint doi: https://doi.org/10.64898/2025.12.30.696887; this version posted February 6, 2026. The copyright holder for this preprint (which was not certified by peer review) is the author/funder. This article is a US Government work. It is not subject to copyright under 17 USC 105 and is also made available for use under a CC0 license.



The 2-by-2 heatmaps to visualize the interaction between molecular cell identity (stellate vs. pyramidal) and by sex for all activity parameters were generated by taking the difference between the average WT and PS19 values (or PS19 and WT when high activity values are considered a deficit, as noted in the figures) and the significance corresponds to an unpaired t-test. The 1-by-3 heatmaps for behavior are generated using the same method. The 2-by-2 heatmap for pTau overlap represents the stellate or pyramidal cell overlap with pTau. The 2-by-2 heatmap for pTau intensity represents the mean intensity of each sex/cell type group.

The averaged tau accumulation and activity heatmaps were generated using all tau accumulation or activity data heatmaps (those in **Figs. 6A, B**). To standardize the scale of the heatmaps, each tau accumulation or activity heatmap was normalized by the maximum of the absolute value of the heatmap values, so the values of all the heatmaps ranged between -1 and 1. These standardized heatmaps were averaged to generate the final average heatmaps.

## General data analysis and statistics

Image processing was performed using previously published MATLAB (MathWorks, version R2015aSP1) codes as cited above and Suite2p (v0.11.1)[183]. Data analysis was performed using ImageJ (Fiji, version 1.53q), MATLAB (MathWorks, versions R2015aSP1 and 2020a), and GraphPad Prism (version 9.3.1). Linear correlations and the corresponding r and p values were calculated using Pearson's linear correlation. Significance values for comparing between group means were calculated using unpaired Student's t test, paired Student's t test, or ANOVA tests (two-way ordinary or repeated measures ANOVA; sphericity is assumed for all tests), or Linear Mixed-Effects model (REML, random intercepts for subjects, missing values were excluded, categorical predictors were effects-coded, significance was tested via ANOVA with Satterthwaite's method) as noted. Where noted, multiple pairwise comparisons were performed following ANOVA tests using unpaired Student's t test. P values were adjusted for multiple comparisons using the Bonferroni–Holm method as noted. For the survival curve analysis, groups were compared using a log-rank (Mantel-Cox) test. For fitting success rate to one-phase association exponential functions, an extra sum-of-squares F test was used to select between model fits as described above. For this test, a p-value of 1 indicates the simpler model fit the data better than the more complex model, and so the F test was not performed. Significance values for averaged activity heatmap used one-sample Student's t-test, comparing the normalized activity values within each square to 0. Two-tailed tests were used for all analyses. P values less than 0.05 were considered significant (* $< 0.05$, ** $<0.01$, *** $<0.001$). All line plots show mean and standard error. In all violin plots, horizontal line represents mean, white circle represents median, and whiskers represent interquartile range. Violins represent the kernel density estimation of the data. Detailed statistical information for all figures can be found in **Table S1**.
