# Supplementary material for: Aberrant medial entorhinal cortex dynamics link tau pathology to spatial memory impairment
Source: bioRxiv. 2025 Dec 31:2025.12.30.696887. Preprint. [Version 1] doi: 10.64898/2025.12.30.696887 (PMC12776407; doi:10.64898/2025.12.30.696887)
Supplement: Supplementary file 1 [file NIHPP2025.12.30.696887v1-supplement-2.pdf]

bioRxiv preprint doi: https://doi.org/10.64898/2025.12.30.696887; this version posted December 31, 2025. The copyright holder for this preprint (which was not certified by peer review) is the author/funder. This article is a US Government work. It is not subject to copyright under 17 USC 105 and is also made available for use under a CC0 license.

Supplementary Information for

Aberrant medial entorhinal cortex dynamics link tau pathology to spatial memory impairment

Taylor J. Malone[1], Kyle Cekada[1,2], Jean Tyan[1,3], Lujia Chen[1], Garret Wang[1,4], Yi Gu[1,5*]

Correspondence to: yi.gu@nih.gov

bioRxiv preprint doi: https://doi.org/10.64898/2025.12.30.696887; this version posted December 31, 2025. The copyright holder for this preprint (which was not certified by peer review) is the author/funder. This article is a US Government work. It is not subject to copyright under 17 USC 105 and is also made available for use under a CC0 license.

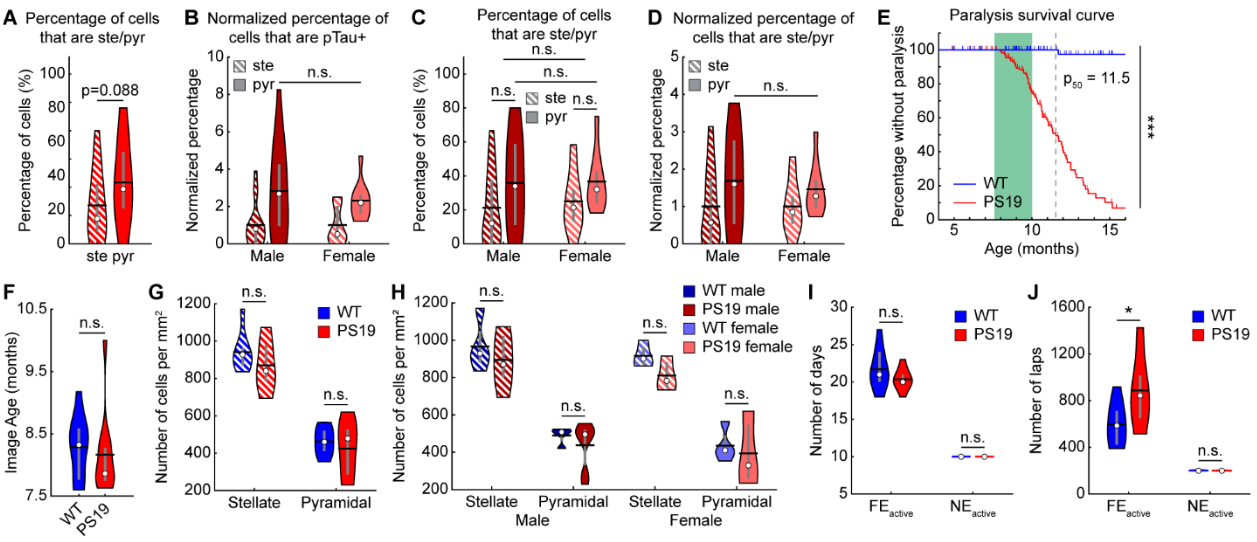

Figure S1: Pyramidal cells in PS19 mice show increased tau pathology. Related to **Figure 1**

A. Percentage of pTau+ cells overlapping with stellate (ste) and pyramidal (pyr) cells in all PS19 mice.

B. Percentage of stellate and pyramidal cells overlapping with pTau+ cells in PS19 mice split by sex and normalized by stellate level for each sex.

C, D. Percentage of pTau+ cells overlapping with stellate and pyramidal cells in PS19 mice split by sex. **C.** Raw overlap percentage. **D.** Normalized by stellate level for each sex.

A-D. Paired/Unpaired Student's t-test.

E. Survival curve representing cumulative onset of hindlimb paralysis in WT and PS19 mice. Dashed line represents median PS19 value (p_{50}). Green box represents age range of imaged mice. Ticks represent censored data points. Log-rank test.

F. Mouse age at the start of imaging.

G, H. Number of stellate and pyramidal cells per area in all mice (**G**) or for each sex (**H**).

I, J. Number of training or imaging days (**I**) or laps (**J**) experienced in the familiar enviorment during active learning (training and imaging) or in the novel enviornment during imaging.

F-J. Unparied Student's t-test.

$*p \leq 0.05$, $**p \leq 0.01$, $***p \leq 0.001$. In violin plots, horizontal line represents mean, white circle represents median, and whiskers represent interquartile range. Statistical details can be found in **Table S1**.

bioRxiv preprint doi: https://doi.org/10.64898/2025.12.30.696887; this version posted December 31, 2025. The copyright holder for this preprint (which was not certified by peer review) is the author/funder. This article is a US Government work. It is not subject to copyright under 17 USC 105 and is also made available for use under a CC0 license.

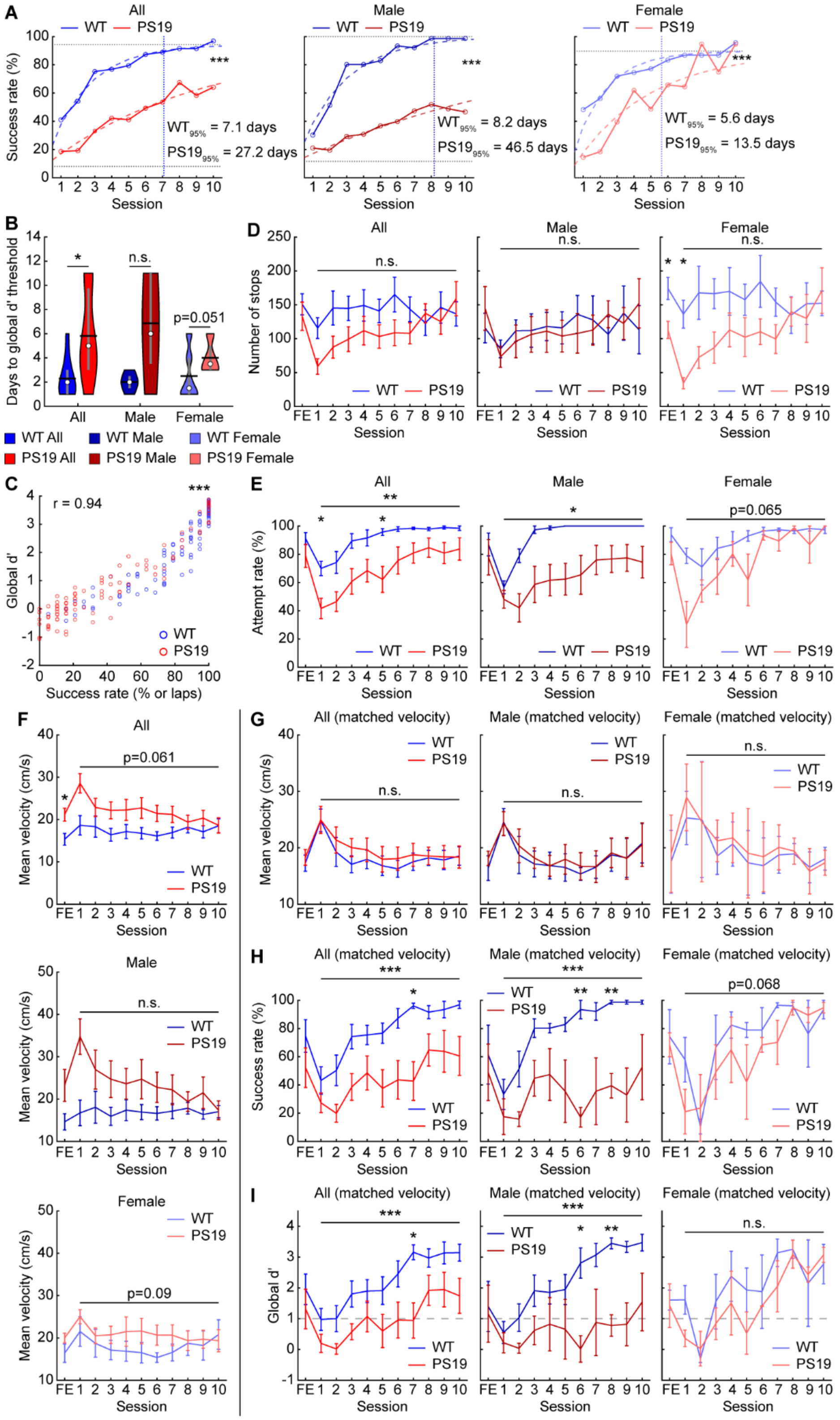

Figure S2: Male PS19 mice show behavioral deficits in an active spatial learning task. Related to **Figure 1**
A. Percentage of laps that mice successfully stopped to receive water reward across learning in all mice (left), male mice (middle), and female mice (right). $WT_{95\%}$ and $PS19_{95\%}$: number of days to reach 95% of plateau value.

bioRxiv preprint doi: https://doi.org/10.64898/2025.12.30.696887; this version posted December 31, 2025. The copyright holder for this preprint (which was not certified by peer review) is the author/funder. This article is a US Government work. It is not subject to copyright under 17 USC 105 and is also made available for use under a CC0 license.

Solid lines and circles: mean success rate of each group from **Fig. 1N**. Dashed colored curves: fit of one-phase association exponential. Horizontal dotted lines: mutual baseline and plateau values used for exponential fit. Vertical dotted line: number of days for WT mice to reach 95% of plateau value. Extra sum-of-squares F test. All mice: $WT_K = 0.42$, $PS19_K = 0.11$. Male mice: $WT_K = 0.37$, $PS19_K = 0.064$. Female mice: $WT_K = 0.53$, $PS19_K = 0.22$.

B. Number of days for global reward zone discrimination (global d') to reach the learning threshold of 1. Unpaired t-test.

C. Correlation between success rate and global d' for all mice and sessions. Pearson correlation was calculated for both genotypes combined.

D. Number of stops per session across learning in all mice (left), male mice (middle), and female mice (right).

E. Percentage of laps that mice attempted to receive water reward across learning in all mice (left), male mice (middle), and female mice (right). An attempt was defined as 0.5 seconds (decreased from 1s) with speed below 5 cm/s (increased from 1 cm/s) within a 100 cm zone centered on the true 50 cm reward zone.

F. Mean session velocity across learning in all mice (top), male mice (middle), and female mice (bottom).

G-I. Mean session velocity (**G**), success rate (**H**), and global d' (**I**) across learning in velocity matched mice for all mice (left), male mice (middle), and female mice (right). Velocity was matched by selecting pairs of mice on each day with similar velocity (within 5 cm/s).

D-I. Horizontal gray bars indicate p values for the group difference (**D-F:** two-way repeated measures ANOVA, **G-I:** ordinary two-way ANOVA). Individual time points are compared using Student's t-test with Bonferroni-Holm correction.

$*p \leq 0.05$, $**p \leq 0.01$, $***p \leq 0.001$. In violin plots, horizontal line represents mean, white circle represents median, and whiskers represent interquartile range. Error bars in line plots represent mean ± sem. Statistical details can be found in **Table S1**.

bioRxiv preprint doi: https://doi.org/10.64898/2025.12.30.696887; this version posted December 31, 2025. The copyright holder for this preprint (which was not certified by peer review) is the author/funder. This article is a US Government work. It is not subject to copyright under 17 USC 105 and is also made available for use under a CC0 license.

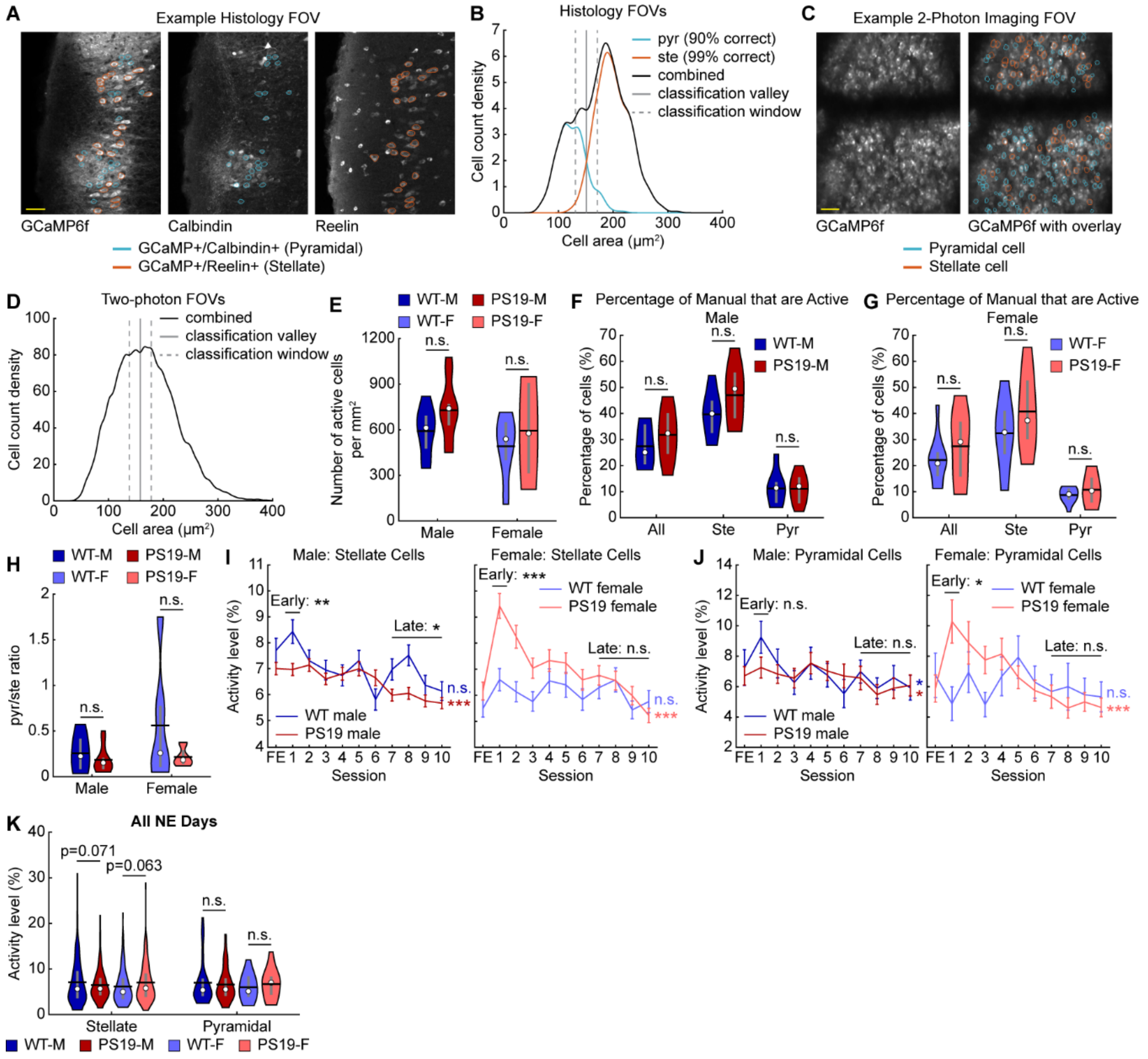

Figure S3: MEC cells of PS19 mice show hyper- or hypoactivity depending on molecular identity and sex. Related to **Figure 2**.

A. Example histology FOV showing the overlap of reelin (stellate cell marker) and calbindin (pyramidal cell marker) with GCaMP6f. Cells were manually outlined if they had both GCaMP6f and reelin or calbindin staining. Used to validate differentiation of stellate and pyramidal cells based on cell area. Scale bar: 50µm

B. Histology validation using area to differentiate stellate (ste) and pyramidal (pyr) cells. Colored lines indicate the true size distribution of stellate and pyramidal cells. The true positve rate of stellate and pyramidal cell idenification segementation (gray lines) using the combined area distribution (black line) is indicated.

C. Example calcium imaging FOV with no overlay (left) and with an overlay of manually outlined cells identified as pyramidal or stellate cells (right). Scale bar: 50µm

D. The size distribution of manually outlined cells from the FE day of calcium imaging with the segmentation lines marked as in **B** (158 +/- 20µm).

E. The number of active cells per area on the FE day split by sex. WT-M: WT Male, PS19-M: PS19 Male, WT-F: WT Female, PS19-F: PS19 Female

F, G. The percentage of all manually outlined cells and manually outlined stellate and pyramidal cells in male (**F**) or female (**G**) mice that are active on the FE day.

bioRxiv preprint doi: https://doi.org/10.64898/2025.12.30.696887; this version posted December 31, 2025. The copyright holder for this preprint (which was not certified by peer review) is the author/funder. This article is a US Government work. It is not subject to copyright under 17 USC 105 and is also made available for use under a CC0 license.

H. The ratio between the fraction of active cells that are identified as pyramidal cells and as stellate cells split by sex. A low value indicates stellate cell enrichment.

I, J. Activity level across learning in male (left) and female (right) mice in stellate (**I**) and pyramidal cells (**J**).

K. Activity level averaged across all NE days split by sex and morphological cell type.

*$p \leq 0.05$, **$p \leq 0.01$, ***$p \leq 0.001$. In violin plots, horizontal line represents mean, white circle represents median, and whiskers represent interquartile range. Violin plots use unpaired Student's t-test. Error bars in line plots represent mean ± sem. In line plots, horizontal gray bars indicate p values for the group difference (early learning: unpaired Student's t-test, late learning: two-way repeated measures ANOVA), and p-values to the right indicate significant Pearson correlation of the mean value with respect to time. Statistical details can be found in **Table S1**.

bioRxiv preprint doi: https://doi.org/10.64898/2025.12.30.696887; this version posted December 31, 2025. The copyright holder for this preprint (which was not certified by peer review) is the author/funder. This article is a US Government work. It is not subject to copyright under 17 USC 105 and is also made available for use under a CC0 license.

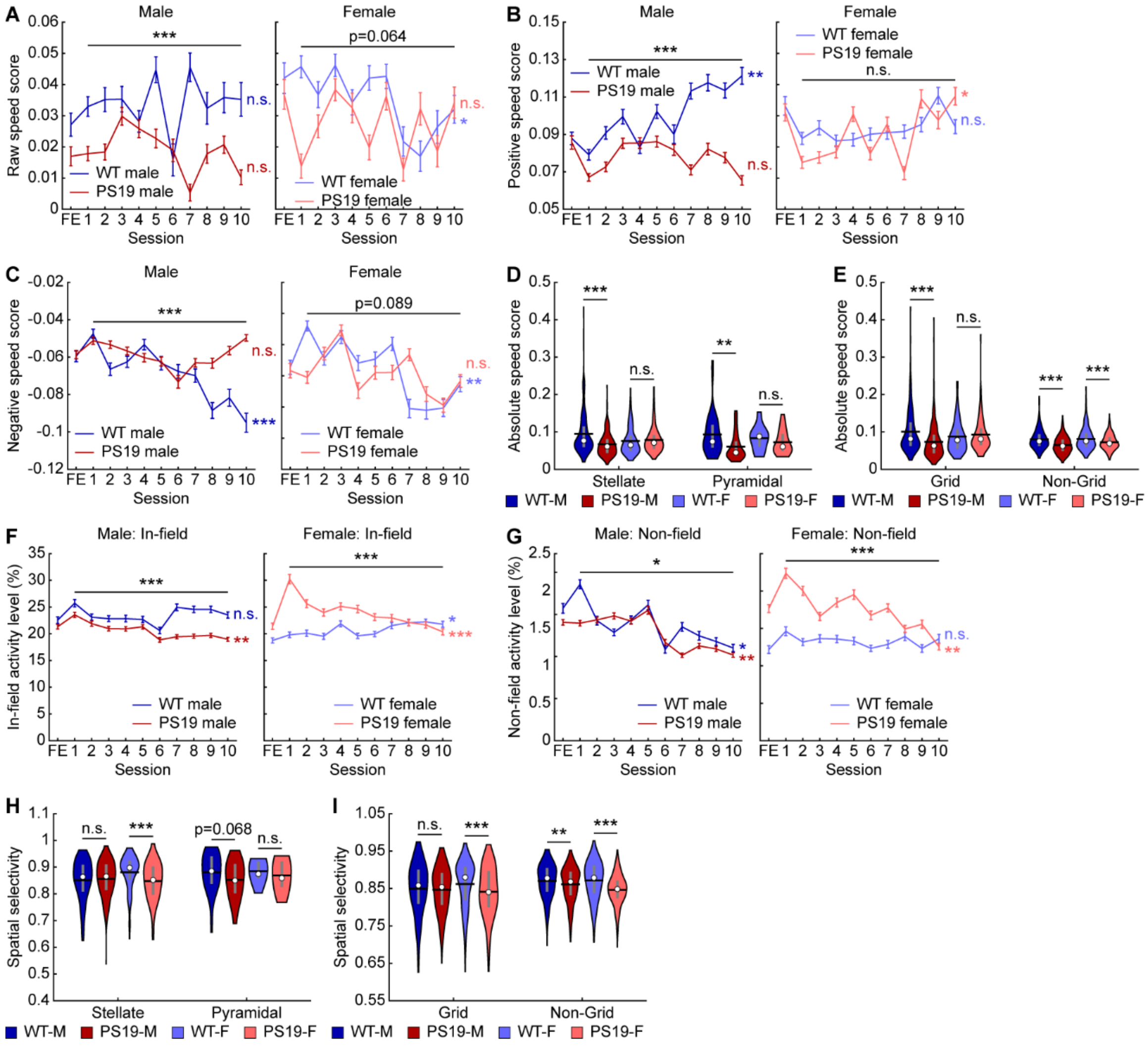

Figure S4: PS19 mice have altered speed modulation and spatial selectivity. Related to **Figure 2**.

A. Raw speed score across learning in male (left) and female (right) mice.

B, C. Positive (**B**) and negative (**C**) speed scores across learning in male (left) and female (right) mice.

D, E. Absolute speed score averaged across all NE days split by sex and morphological (**D**) and functional (**E**) cell type.

F, G. Activity level across learning in male (left) and female (right) mice within track regions with fields (In-field, **F**) and in other track locations (Non-field, **G**).

H, I. Spatial selectivity averaged across all NE days split by sex and morphological (**H**) and functional (**I**) cell type.

$*p \leq 0.05$, $**p \leq 0.01$, $***p \leq 0.001$. In violin plots, horizontal line represents mean, white circle represents median, and whiskers represent interquartile range. Violin plots use unpaired Student's t-test. Error bars in line plots represent mean ± sem. In line plots, horizontal gray bars indicate p values for the group difference (early learning: unpaired Student's t-test, all learning days: linear mixed-effects model), and p-values to the right indicate significant Pearson correlation of the mean value with respect to time. Statistical details can be found in **Table S1**.

bioRxiv preprint doi: https://doi.org/10.64898/2025.12.30.696887; this version posted December 31, 2025. The copyright holder for this preprint (which was not certified by peer review) is the author/funder. This article is a US Government work. It is not subject to copyright under 17 USC 105 and is also made available for use under a CC0 license.

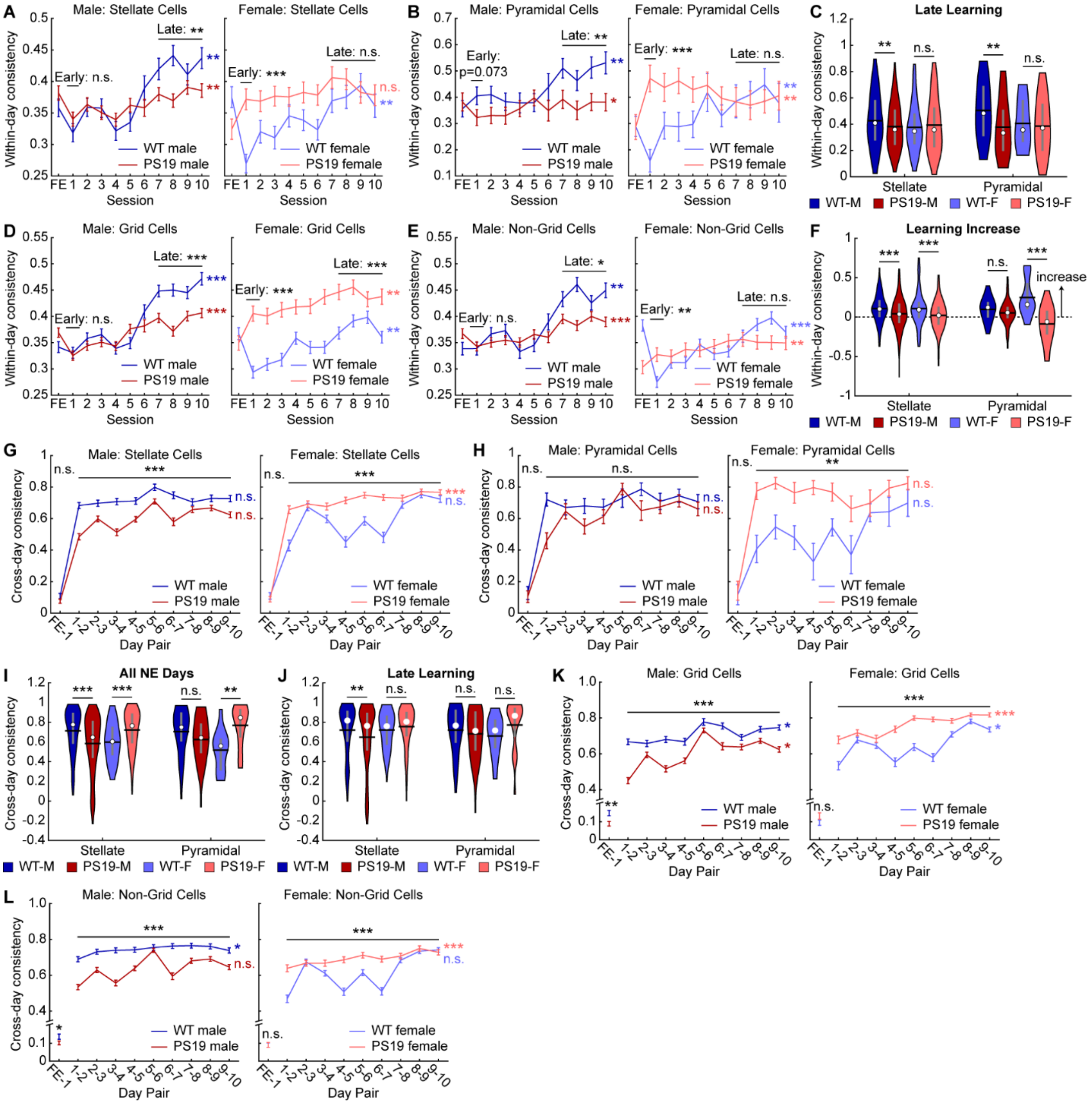

Figure S5: Male PS19 mice have impaired MEC within-day and cross-day activity consistency. Related to **Figure 3**.

A, B. Within-day activity consistency across learning in male (left) and female (right) mice in stellate (**A**) and pyramidal cells (**B**).

C. Within-day activity consistency averaged across late learning (days 7-10) split by sex and morphological cell type. WT-M: WT Male, PS19-M: PS19 Male, WT-F: WT Female, PS19-F: PS19 Female

D, E. Within-day activity consistency across learning in male (left) and female (right) mice in grid (**D**) and non-grid cells (**E**).

F. The increase in within-day activity consistency between early learning (day 1) and late learning split by sex and morphological cell type.

G, H. Cross-day activity consistency across learning in male (left) and female (right) mice in stellate (**G**) and pyramidal cells (**H**).

bioRxiv preprint doi: https://doi.org/10.64898/2025.12.30.696887; this version posted December 31, 2025. The copyright holder for this preprint (which was not certified by peer review) is the author/funder. This article is a US Government work. It is not subject to copyright under 17 USC 105 and is also made available for use under a CC0 license.

I, J. Cross-day activity consistency averaged across all learning days (**I**) and late learning (**J**) split by sex and morphological cell type.

K, L. Cross-day activity consistency across learning in male (left) and female (right) mice in grid (**K**) and non-grid cells (**L**).

*$p \leq 0.05$, **$p \leq 0.01$, ***$p \leq 0.001$. In violin plots, horizontal line represents mean, white circle represents median, and whiskers represent interquartile range. Violin plots use unpaired Student's t-test. Error bars in line plots represent mean ± sem. In line plots, horizontal gray bars indicate p values for the group difference (early learning/ FE-1: unpaired Student's t-test, late learning/all learning days: two-way repeated measures ANOVA or linear mixed-effects model), and p-values to the right indicate significant Pearson correlation of the mean value with respect to time. Statistical details can be found in **Table S1**.

bioRxiv preprint doi: https://doi.org/10.64898/2025.12.30.696887; this version posted December 31, 2025. The copyright holder for this preprint (which was not certified by peer review) is the author/funder. This article is a US Government work. It is not subject to copyright under 17 USC 105 and is also made available for use under a CC0 license.

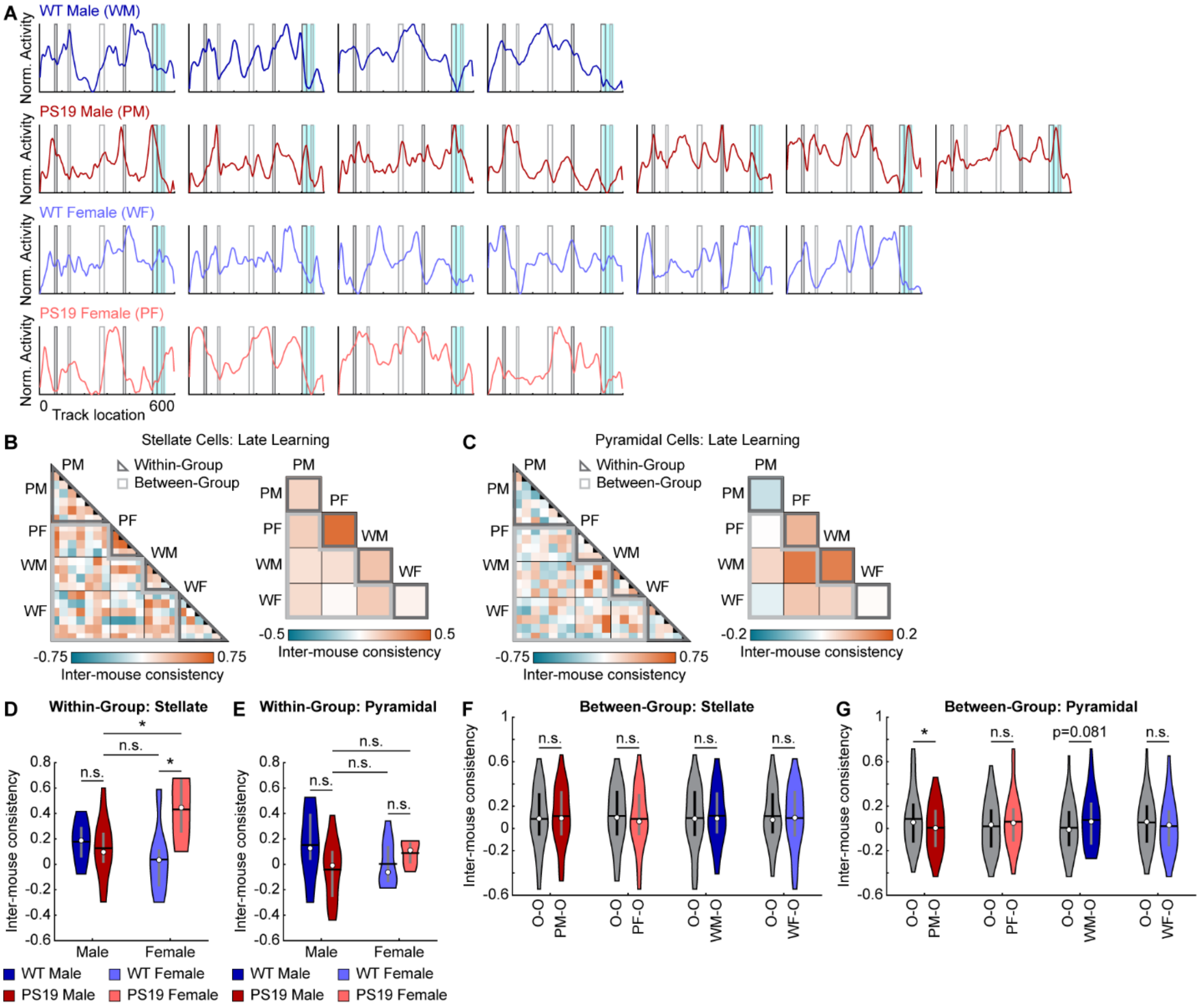

Figure S6: Male PS19 mice do not develop a cognitive map shared by other mouse groups. Related to **Figure 4**.
A. Late-learning spatial activity maps as calculated in **Fig. 4A** for individual mice. From top to bottom: WT male, PS19 male, WT female, PS19 female. Black and grey rectangles represent left and right cues, respectively. Blue rectangle represents reward zone.
B, C. Inter-mouse consistency for stellate (**B**) and pyramidal cells (**C**). Left: Pairwise inter-mouse consistency sorted by genotype/sex group. Right: Average inter-mouse consistency for each group-to-group block.
D, E. Pooled inter-mouse consistency for pairwise within-group comparisons for stellate (**D**) and pyramidal (**E**) cells. Unpaired Student's t-test with Bonferroni-Holm correction.
F, G. Pooled inter-mouse consistency for pairwise between-group comparisons for stellate (**F**) and pyramidal (**G**) cells with each genotype/sex group serving as the reference group compared to the other genotype/sex groups (O) as indicated. Unpaired Student's t-test.
$*p \leq 0.05$, $**p \leq 0.01$, $***p \leq 0.001$. In violin plots, horizontal line represents mean, white circle represents median, and whiskers represent interquartile range. Statistical details can be found in **Table S1**.

bioRxiv preprint doi: https://doi.org/10.64898/2025.12.30.696887; this version posted December 31, 2025. The copyright holder for this preprint (which was not certified by peer review) is the author/funder. This article is a US Government work. It is not subject to copyright under 17 USC 105 and is also made available for use under a CC0 license.

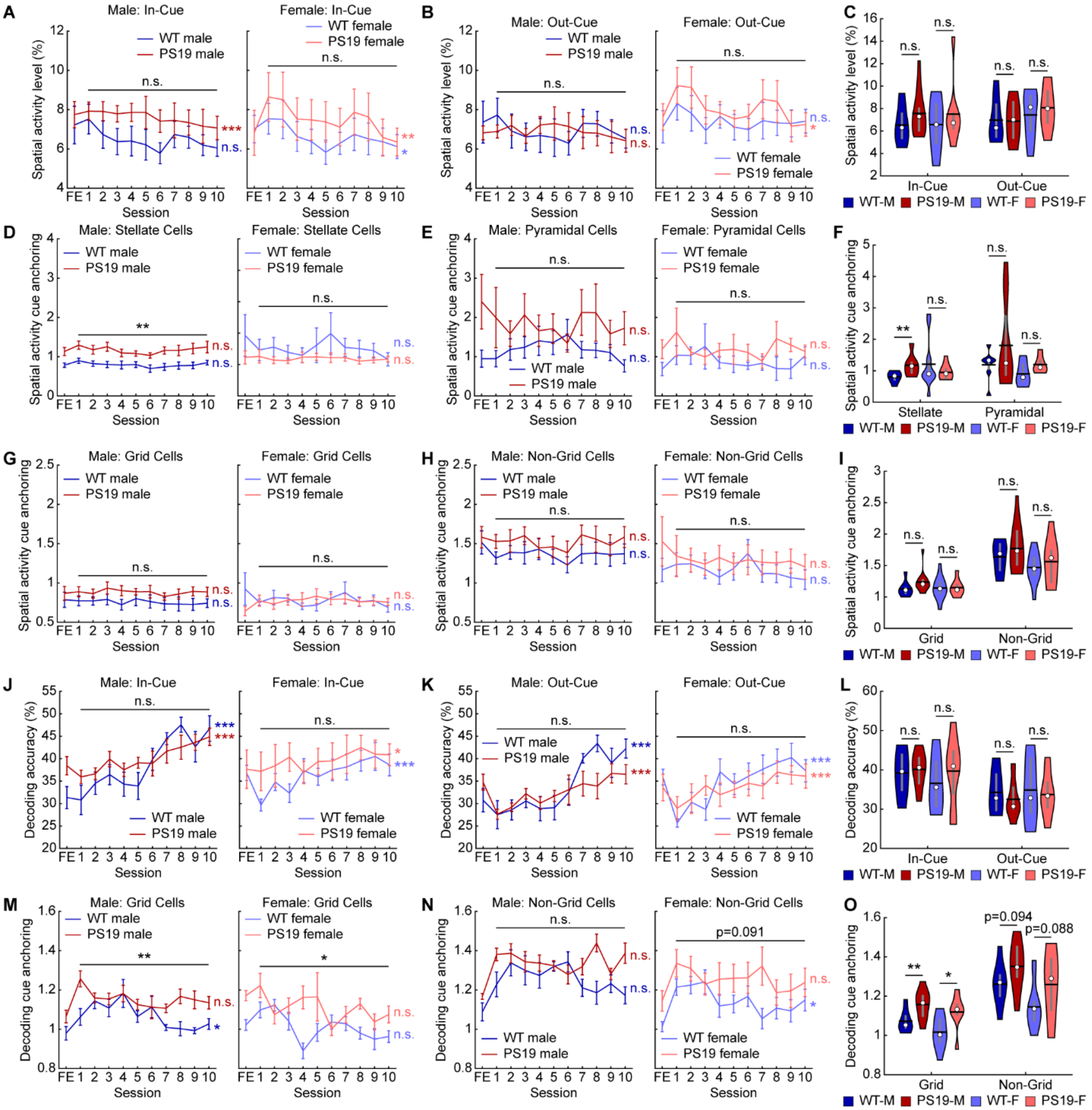

Figure S7: Spatial activity of male PS19 mice is anchored to visual cues. Related to **Figure 5**.

A, B. Spatial activity level within in-cue (**A**) and out-cue (**B**) regions of the track across learning in male (left) and female (right) mice.

C. Spatial activity level within in-cue and out-cue regions of the track averaged across all learning days. WT-M: WT Male, PS19-M: PS19 Male, WT-F: WT Female, PS19-F: PS19 Female

D, E, G, H. Spatial activity cue anchoring across learning in male (left) and female (right) mice in stellate (**D**), pyramidal (**E**), grid (**G**), and non-grid (**H**) cells.

F, I. Spatial activity cue anchoring averaged across all learning days split by morphological (**F**) and functional (**I**) cell type.

J, K. Decoding accuracy within in-cue (**J**) and out-cue (**K**) regions of the track across learning in male (left) and female (right) mice.

bioRxiv preprint doi: https://doi.org/10.64898/2025.12.30.696887; this version posted December 31, 2025. The copyright holder for this preprint (which was not certified by peer review) is the author/funder. This article is a US Government work. It is not subject to copyright under 17 USC 105 and is also made available for use under a CC0 license.

L. Decoding accuracy within in-cue and out-cue regions of the track averaged across all learning days.
M, N. Decoding cue anchoring across learning in male (left) and female (right) mice in grid (**M**) and non-grid (**N**) cells.
O. Decoding cue anchoring averaged across all learning days split by functional cell type.
*$p \leq 0.05$, **$p \leq 0.01$, ***$p \leq 0.001$. In violin plots, horizontal line represents mean, white circle represents median, and whiskers represent interquartile range. Violin plots use unpaired Student's t-test. Error bars in line plots represent mean ± sem. In line plots, horizontal gray bars indicate p values for the group difference (linear mixed-effects model), and p-values to the right indicate significant Pearson correlation of the mean value with respect to time. Statistical details can be found in **Table S1**.

bioRxiv preprint doi: https://doi.org/10.64898/2025.12.30.696887; this version posted December 31, 2025. The copyright holder for this preprint (which was not certified by peer review) is the author/funder. This article is a US Government work. It is not subject to copyright under 17 USC 105 and is also made available for use under a CC0 license.

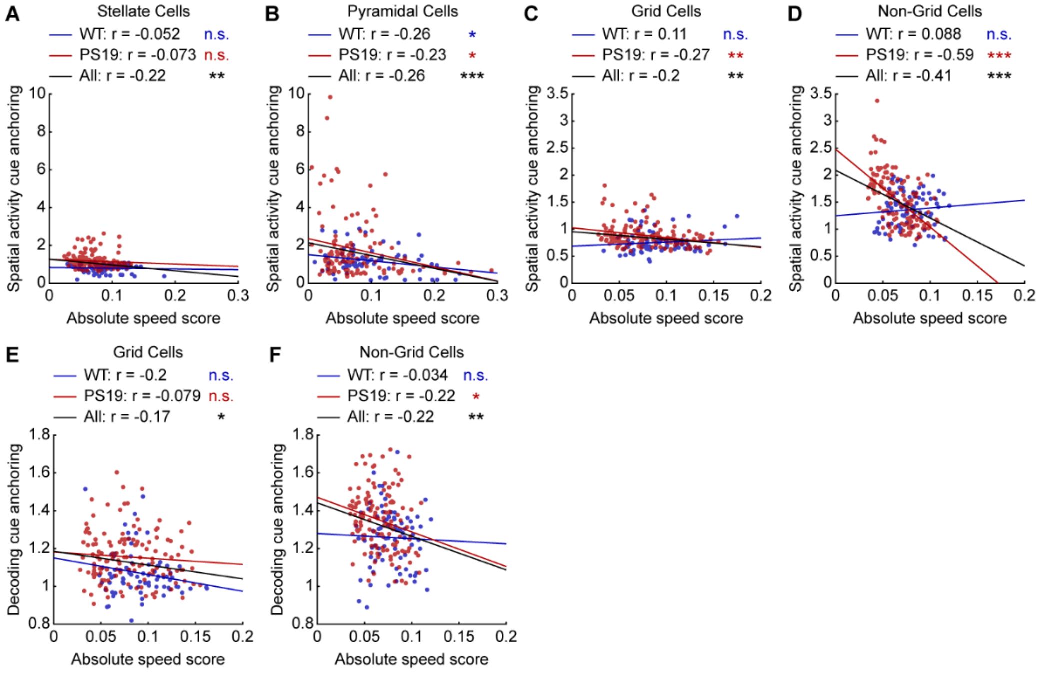

Figure S8: Speed modulation is correlated with cue anchoring in male PS19 mice. Related to **Figure 5**.
A-F. Correlation between absolute speed score and spatial activity cue anchoring (**A-D**) or decoding cue anchoring (**E, F**) in stellate (**A**), pyramidal (**B**), grid (**C, E**), and non-grid (**D, F**) cells in male mice. Each point represents the activity from one FOV from one session. Pearson correlation.
*$p \leq 0.05$, **$p \leq 0.01$, ***$p \leq 0.001$. Statistical details can be found in **Table S1**.

bioRxiv preprint doi: https://doi.org/10.64898/2025.12.30.696887; this version posted December 31, 2025. The copyright holder for this preprint (which was not certified by peer review) is the author/funder. This article is a US Government work. It is not subject to copyright under 17 USC 105 and is also made available for use under a CC0 license.

Activity Category	Activity Features	Learning Period	Male PS19 Compared to Male WT	Female PS19 Compared to Female WT	Figure
General Activity Deficits	Activity Level	Early Learning	Low	High	2D
		Late Learning	Low	Low	2D
	Absolute Speed Score	All NE Days	Low	No difference	2K
	Spatial Selectivity	All NE Days	No difference	Low	2O
Map Consistency	Within-Day Consistency	Late Learning	Low	No difference	3C
		Learning Increase	Low	Low	3E
	Cross-Day Consistency	All NE Days	Low	High	3I
		Late Learning	Low	High	3J
	Inter-Mouse Consistency	All NE Days	Low (compared to others)	High (compared to others)	4H, I
Global Representation	Spatial Activity Cue Anchoring	All NE Days	High	No difference	5C
	Decoding Cue Anchoring	All NE Days	High	No difference	5J

Under-Effect

Over-Effect

Figure S9: Summary table of activity under-effects and over-effects in PS19 mice relative to sex-matched WT mice.

bioRxiv preprint doi: https://doi.org/10.64898/2025.12.30.696887; this version posted December 31, 2025. The copyright holder for this preprint (which was not certified by peer review) is the author/funder. This article is a US Government work. It is not subject to copyright under 17 USC 105 and is also made available for use under a CC0 license.

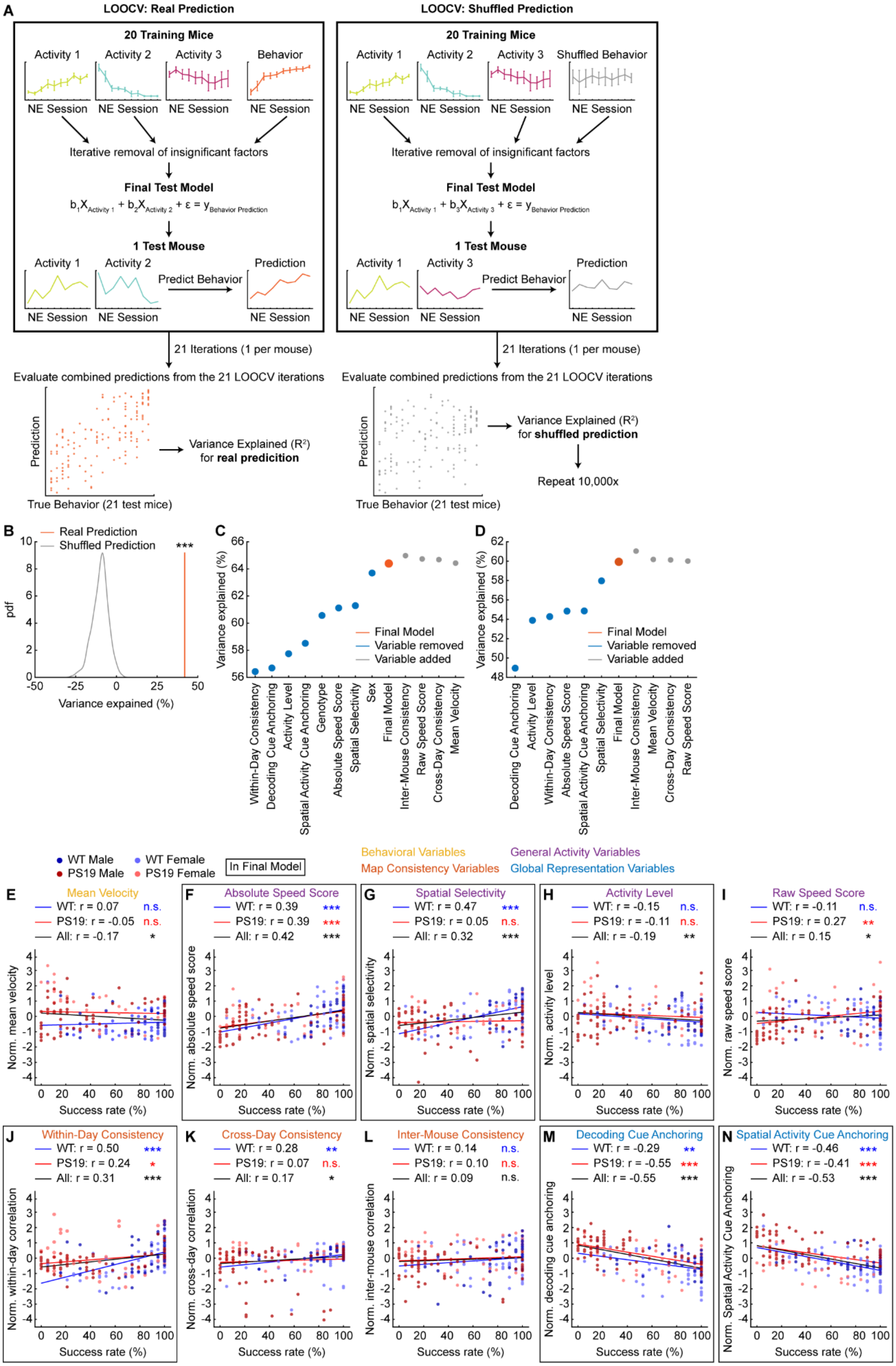

bioRxiv preprint doi: https://doi.org/10.64898/2025.12.30.696887; this version posted December 31, 2025. The copyright holder for this preprint (which was not certified by peer review) is the author/funder. This article is a US Government work. It is not subject to copyright under 17 USC 105 and is also made available for use under a CC0 license.

Figure S10: GLM model selection to predict mouse behavior using MEC activity. Related to **Figure 7**.
A. Schematic of final model selection method.
B. Evaluation of model selection method by comparing model predictions based on true behavior with shuffled behavior models.
C. Effect of removing variables from final model or adding excluded variables back to final model on percentage of behavioral variance explained (R^2).
D. Similar to **C** but excluding sex and genotype from all models.
E-N. Correlation between normalized predictor variables and success rate. Each point represents one session from one mouse. Dot colors indicate sex and genotype. Final model variables are outlined in black. Pearson correlations were calculated for all mice, all WT mice, and all PS19 mice. **E**: Mean velocity. **F**: Absolute speed score **G**: Spatial selectivity. **H**: Activity level. **I**: Raw speed score. **J**: Within-day consistency. **K**: Cross-day consistency. **L**: Inter-mouse consistency. **M**: Decoding cue anchoring. **N**: Spatial activity cue anchoring.
*$p \leq 0.05$, **$p \leq 0.01$, ***$p \leq 0.001$. Statistical details can be found in **Table S1**.

bioRxiv preprint doi: https://doi.org/10.64898/2025.12.30.696887; this version posted December 31, 2025. The copyright holder for this preprint (which was not certified by peer review) is the author/funder. This article is a US Government work. It is not subject to copyright under 17 USC 105 and is also made available for use under a CC0 license.

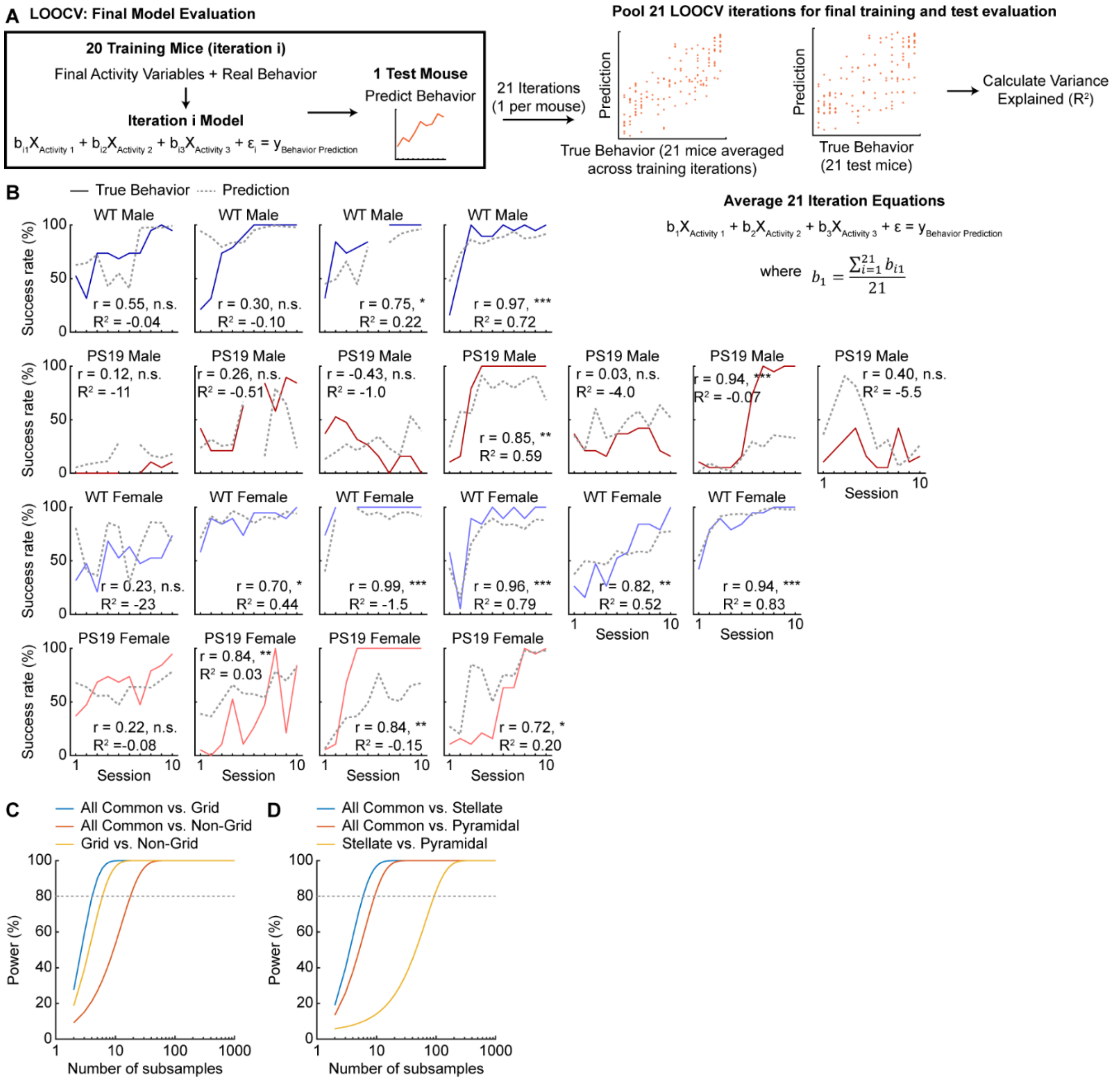

Figure S11: MEC activity changes are predictive of behavioral deficits in PS19 mice. Related to **Figure 7**.
A. Schematic of final model evaluation method.
B. Comparison of mean success rate (colored lines) and model predictions (dashed grey lines) across learning for individual mice (from top to bottom: WT male, PS19 male, WT female, PS19 female). Test predictions from single LOOCV iterations using the left-out mouse were used to calculate prediction.
C. Power analysis comparing the distributions of behavioral variance explained of the subsampled all-common-cell, grid cell, and non-grid cell activity data to determine the probability of obtaining a significant result if a true difference exists for different numbers of subsamples. Dashed line corresponds to 80% power (common vs. grid = 5 subsamples, common vs. non-grid = 18 subsamples, grid vs. non-grid = 7 subsamples).
D. Similar to **C**, but for all-common-cell, stellate cell, and pyramidal cell activity data (common vs. stellate = 6 subsamples, common vs. pyramidal = 10 subsamples, stellate vs. pyramidal = 93 subsamples).
$*p \leq 0.05$, $**p \leq 0.01$, $***p \leq 0.001$. Statistical details can be found in **Table S1**.
